# Supplementary material for: Early evaluation of the ‘STOP SEPSIS!’ WHO Global Maternal Sepsis Awareness Campaign implemented for healthcare providers in 46 low, middle and high-income countries
Source: BMJ Open. 2020 May 21;10(5):e036338. doi: 10.1136/bmjopen-2019-036338 (PMC7247401; doi:10.1136/bmjopen-2019-036338)
Supplement: Supplementary data [file bmjopen-2019-036338supp001.pdf]

Appendix 1. Global Maternal Sepsis Study – Pre- and Post-Campaign Surveys

GLOSS Pre-Campaign Survey

This online survey is part of the activities set forth for a global study on maternal morbidity and mortality. This study is being conducted in approximately 50 countries across the globe, including your own, and it is coordinated by the World Health Organization and the healthcare facility where you work.

As part of this study, we want to learn more from healthcare providers about how you identify and manage women with complications during pregnancy, childbirth, postpartum, or post- abortion. The survey includes a number of questions on your knowledge, attitudes, and practices around maternal and neonatal health. This is not a test; this is an opportunity to let us know your thoughts and experience on the topic as a healthcare provider in one of the hospitals participating in the study.

This survey is voluntary, and your answers will be kept confidential, and you can choose whether to leave some questions unanswered. General information about you, your position, and geographical location will be collected to help us categorize respondents only but will not be used to identify you in particular. You are free to provide this information at the end of the survey.

After the study, and only if you agree, a second online survey will be sent to you via email. For this reason, we will ask you to provide an email address so that we can ensure delivery of the second survey. You will be free to decide to participate in this second survey too. Results of these surveys will be published in a peer-reviewed journal without attributing responses to any specific person or institution.

The completion of this survey implies your consent to participate.

If you have any question about the survey, please contact Ms Vanessa Brizuela (brizuelav@who.int)

Brizuela V, *et al. BMJ Open* 2020; 10:e036338. doi: 10.1136/bmjopen-2019-036338

I. Knowledge and attitudes

The following questions will ask that you respond according to your current role, competences, and skills depending on your training and background. That is, according to these, you may be the person triaging, prescribing, diagnosing, treating. Bear this in mind when responding.

1. What are the main conditions causing death and disability among women during pregnancy and/or childbirth in your hospital? Check all that apply [abortion-related complications, chronic/pre-existing disease, embolism, haemorrhage, infection/sepsis, pre-eclampsia/eclampsia, other: please specify]
2. Case vignettes:

   Case A: A 25-year-old 32- week pregnant woman comes to your facility brought by a family member saying she is feeling unwell. Her companion reports that she seems a bit disoriented and feverish. Without any further diagnostic testing or triaging:

   a. What would you first think could be causing her to feel this way? Choose from the following list [abortion-related complications, embolism, haemorrhage, infection/sepsis, pre-eclampsia/eclampsia, other]
   b. What would be the first two things this woman should receive? [antibiotics, blood transfusion, body fluid culture, fetal monitoring, fluids, haematology/biochemistry laboratory, other antimicrobials (i.e. antimalarials, ART), other laboratory test, other medication, oxygen, physical exam, urine output measurement, other]
3. Case B: A recently pregnant woman comes to your facility complaining that she has abdominal pain and shortness of breath. Without any further diagnostic testing or triaging:
   a. What would you first think could be causing her to feel this way? Choose from the following list [abortion-related complications, embolism, haemorrhage, infection/sepsis, pre-eclampsia/eclampsia, other]
   b. What would be the first two things this woman should receive? [antibiotics, blood transfusion, body fluid culture, fetal monitoring, fluids, haematology/biochemistry laboratory, other antimicrobials (i.e. antimalarials, ART), other laboratory test, other medication, oxygen, physical exam, urine output measurement, other]
4. How confident do you feel that you are capable of making the right decision in a case like the one above? [very confident, somewhat confident, neutral, not too confident, not confident at all]
5. How would you qualify the availability of resources in the facility where you work to help you make the right decisions? [always available, somewhat available, neutral, not always available, not available at all].
6. How supported do you feel by the facility in which you work to make the right decision in a case like the one above? [very supported, somewhat supported, neutral, not very supported, unsupported].
7. How well does this statement describe your facility: “The facility where I work doesn’t let me handle cases like the one described above.” [very well, somewhat well, indifferent, somewhat incorrectly, completely incorrectly]

Brizuela V, *et al. BMJ Open* 2020; 10:e036338. doi: 10.1136/bmjopen-2019-036338

8. Of the following, which do you think are the greatest barriers in making a right, and timely decision in your facility? Check up to two options. [I'm afraid of making a mistake, I've never seen cases like these, my supervisor doesn't let me make them, not sure I know the correct signs, we don't have a way to triage/treat/manage cases like these in my hospital, other]
9. Does the hospital you work in have protocols in place for dealing with cases like the one described above? [yes/no/don't know]

DO NOT ALLOW GOING BACK AFTER THIS QUESTION

10. Have you ever heard of the term maternal sepsis? [yes/no]
11. If yes, how did you hear about this? Check all that apply [pre-service training, in-service training, public health campaign, colleagues, media (TV/radio/newspaper), other: please specify].
12. What two criteria best describe maternal sepsis? Check two options [abnormal white cell count, altered mental status, elevated heart rate, excessively rapid respiration, fever, infection, low blood pressure, organ dysfunction, other]
13. What supplies/commodities are essential to effectively identify sepsis among women during pregnancy, childbirth, postpartum or post-abortion? Check all that apply [blood culture, blood pressure apparatus, diagnostic imaging, laboratory (haematology/biochemistry), rapid test for infectious disease, serum lactate measurement, thermometer, urine output measurement, other]
14. What supplies/commodities are essential to effectively manage sepsis in women during pregnancy, childbirth, postpartum or post-abortion? Check all that apply [antibiotics, blood transfusions, fluids, intensive care/high-dependency unit, other antimicrobials (e.g. antimalarials, ART), oxygen, urine output measurement, other]

II. Context

15. How many women are affected by maternal sepsis in your facility every year? Give your best estimate (a whole number), given your experience in the facility [Text box]
16. How many neonates are affected by neonatal sepsis in the first week of life in your facility? Give your best estimate (a whole number), given your experience in the facility. [Text box]
17. How many deliveries occur every year, on average, in your facility? Give your best estimate (a whole number). [Text box]
18. Have you ever received specific training in how to manage women who present with signs of infection while pregnant, during childbirth, postpartum or post-abortion? [yes/no/can't remember]

III. Personal information

Remember! These data are collected for categorization purposes only. Your information is confidential and you will not be identified in any future publications on this study.

19. Age range
20. Gender: [male, female, other]

Brizuela V, *et al. BMJ Open* 2020; 10:e036338. doi: 10.1136/bmjopen-2019-036338

21. Qualification: [nurse, midwife, physician/medical doctor, resident/physician in training, community health worker, social worker, other: please specify].
22. Years of work experience in current setting: [years|months]
23. Total years of work experience (since completing your training): [number entry/2-digit max]
24. Place of work: [list of countries]
25. Location (of current or main place of work): [urban/rural]
26. Name of facility & address [Text box]
27. Facility type (of current or main place of work): [Clinic, Health centre, Maternity hospital, Regional/Provincial hospital, District hospital, Other hospital, other]
28. Facility management (of current or main place of work): [private, public, social insurance, NGO, other]
29. Did you participate in this year's World Sepsis Congress Spotlight on Maternal and Neonatal Sepsis (held on 12 September 2017)? [yes/no]

IV. Future contacts

30. The global maternal study and awareness campaign would like to contact you at a future date for a follow-up on this survey. If you agree to being contacted again, please provide us with your email address.

Your contact details will be securely stored by the WHO staff person working on the study for one year. You can contact us to modify or suppress your information at any time. To do so, please contact Ms Vanessa Brizuela (brizuelav@who.int).

- ☐ I agree.
- ☐ I do not agree

Please provide us with your email address [text box]

Thank you very much for participating in this survey! Your responses are extremely valuable to us in our efforts to improve the health of women and newborns.

GLOSS Post-Campaign Survey

This online survey is part of the activities set forth for a global study on maternal morbidity and mortality. This study is being conducted in approximately 50 countries across the globe, including your own, and it is coordinated by the World Health Organization and the healthcare facility where you work. As part of this study, we want to learn more from healthcare providers about how you identify and manage women with certain conditions during pregnancy, childbirth or postpartum or post- abortion.

A few months ago, you received a similar survey from us. At that point we asked that you provide us with an email address so that we could send you a follow-up questionnaire. We will ask again that you respond to a number of questions on your knowledge, attitudes, and practices around maternal and neonatal health. This is not a test; this is an opportunity to let us know your thoughts and experience on the topic as a healthcare provider in one of the hospitals participating in the study.

This survey is voluntary, and your answers will be kept confidential, and you can choose whether to leave some questions unanswered. General information about you, your position, and geographical location will be collected to help us categorize respondents only but will not be used to identify you in particular. You are free to provide this information at the end of the survey. Results of these surveys will be published in a peer-reviewed journal without attributing responses to any specific person or institution.

The completion of this survey implies your consent to participate.

If you have any question about the survey, please contact Ms Vanessa Brizuela (brizuelav@who.int)

Brizuela V, *et al. BMJ Open* 2020; 10:e036338. doi: 10.1136/bmjopen-2019-036338

I. General knowledge and attitudes

The following questions will ask that you respond according to your current role, competences, and skills depending on your training and background. According to these, you may be the person triaging, prescribing, diagnosing, treating. Bear this in mind when responding.

1. What are the main conditions causing death and disability among women during pregnancy and/or childbirth in your hospital? Check all that apply [abortion-related complications, chronic/pre-existing disease, embolism, haemorrhage, infection/sepsis, pre-eclampsia/eclampsia, other: please specify]
2. Case vignettes:

   Case A: A 25-year-old 32- week pregnant woman comes to your facility brought by a family member saying she is feeling unwell. Her companion reports that she seems a bit disoriented and feverish. Without any further diagnostic testing or triaging:

   a. What would you first think could be causing her to feel this way? Choose from the following list [abortion-related complications, embolism, haemorrhage, infection/sepsis, pre-eclampsia/eclampsia, other]
   b. What would be the first two things this woman should receive? [antibiotics, blood transfusion, body fluid culture, fetal monitoring, fluids, haematology/biochemistry laboratory, other antimicrobials (i.e. antimalarials), other laboratory test, other medication, oxygen, physical exam, urine output measurement, other]
3. Case B: A recently pregnant woman comes to your facility complaining that she has abdominal pain and shortness of breath. Without any further diagnostic testing or triaging:
   a. What would you first think could be causing her to feel this way? Choose from the following list [abortion-related complications, embolism, haemorrhage, infection/sepsis, pre-eclampsia/eclampsia, other]
   b. What would be the first two things this woman should receive? [antibiotics, blood transfusion, body fluid culture, fetal monitoring, fluids, haematology/biochemistry laboratory, other antimicrobials (i.e. antimalarials, ART), other laboratory test, other medication, oxygen, physical exam, urine output measurement, other]
4. How confident do you feel that you are capable of making the right decision in a case like the one above? [very confident, somewhat confident, indifferent/neutral, not too confident, not confident at all] n
5. How would you qualify the availability of resources in the facility where you work to help you make the right decisions? [always available, somewhat available, indifferent/neutral, not always available, not available at all].
6. How supported do you feel by the facility in which you work to make the right decision in a case like the one above? [very supported, somewhat supported, neutral, not very supported, unsupported].
7. How well does this statement describe your facility: "The facility where I work doesn't let me handle cases like the one described above." [very well, somewhat well, indifferent/neutral, somewhat incorrectly, completely incorrectly]

Brizuela V, *et al. BMJ Open* 2020; 10:e036338. doi: 10.1136/bmjopen-2019-036338

8. Of the following, which do you think are the greatest barriers in making a right, and timely decision in your facility? Check one option (the one most accurate to your situation). [not sure I know the correct signs, I'm afraid of making a mistake, my supervisor doesn't let me make them, we don't have a way to triage/treat/manage cases like these in my hospital, I've never seen cases like these, There are no barriers to making a right and timely decision in my facility, other]
9. Does the hospital you work in have protocols in place for dealing with cases like the one described above? [yes/no/don't know]

DO NOT ALLOW GOING BACK AFTER THIS QUESTION

10. Have you ever heard of the term maternal sepsis? [yes/no]
11. If yes, how did you hear about this? Check all that apply [pre-service training, in-service training, colleagues, public health campaign, media (TV/radio/newspaper), other: please specify].
12. What two criteria best describe maternal sepsis? Check two options [abnormal white cell count, altered mental status, elevated heart rate, excessively rapid respiration, fever, infection, low blood pressure, organ dysfunction, other]
13. What supplies/commodities are essential to effectively identify sepsis among women during pregnancy, childbirth, postpartum or post-abortion? Check all that apply [blood culture, blood pressure apparatus, diagnostic imaging, laboratory (haematology/biochemistry), rapid test for infectious disease, serum lactate measurement, thermometer, urine output measurement, other]
14. What supplies/commodities are essential to effectively manage sepsis in women during pregnancy, childbirth, postpartum or post-abortion? Check all that apply [antibiotics, blood transfusions, fluids, intensive care/high-dependency unit, other antimicrobials (e.g. antimalarials, ART), oxygen, urine output measurement, other]

II. Context

15. How many women are affected by maternal sepsis in your facility every year? Give your best estimate (a whole number), given your experience in the facility [Text box]
16. How many neonates are affected by neonatal sepsis in the first week of life in your facility? Give your best estimate (a whole number), given your experience in the facility. [Text box]
17. How many deliveries occur every year, on average, in your facility? Give your best estimate (a whole number). [Text box]
18. Have you ever received specific training in how to manage women who present with signs of infection while pregnant, during childbirth, postpartum or post-abortion? [yes/no/can't remember]

III. Campaign

19. Did you notice any materials in your facility that related to maternal and neonatal sepsis? [yes/no] [if no go to question 27]

Brizuela V, *et al. BMJ Open* 2020; 10:e036338. doi: 10.1136/bmjopen-2019-036338

20. If yes, where did you see them? Check all that apply [labour ward, antenatal ward, postnatal ward, emergency room/department, ICU/high dependency room, patient waiting area, other: please specify]
21. If yes, what materials did you see? Check all that apply [informational posters about the study, graphic materials with information about maternal sepsis, campaign website, social media, press releases, none of the above].
    a. Did you ever visit the website? [yes/no/N/A]
    b. Did you ever tweet/re-tweet a message about the campaign? [yes/no/I don't use Twitter]
22. What were the main messages of these materials? Check all that apply ["stop sepsis", "think sepsis", "right care, right now", "sepsis is life-threatening but when caught early and treated promptly it can be stopped", "just ask, could it be sepsis?", "a world free of sepsis", "together, we can stop maternal sepsis", "stop sepsis save lives", "surviving sepsis", none of the above]
23. Did you find the materials and messaging helpful? [yes/no]
    a. If yes, why? Check all that apply [they provided information about maternal sepsis, they invited me to act, they mentioned things I did not know, they helped explain the study to me, they were visually appealing, they were in a language I could understand, other]
    b. If no, why not? Check all that apply [they didn't provide any new information, I didn't know what to do, the message was confusing, they were difficult to understand, they were in a language I don't speak/read, they had too much text, they had too many images, the colours didn't work in our hospital, they were unappealing, other]
24. Did the materials help you identify cases of women with infection or sepsis? [yes/no]
25. Did the information provided in the materials motivate you to do something differently than before? [yes/no]
    a. If yes, in what way? Check all that apply [to suspect maternal sepsis among women presenting with specific signs, to act fast when I think a woman could have sepsis, to implement health measures to prevent sepsis, to talk about sepsis with others, to learn more about sepsis, other: please specify]
    b. If no, why not? Please explain [text box]
26. In your opinion, did the materials help to increase awareness about maternal sepsis in your facility? [yes/no]
27. Did you know that your facility participated in a global maternal sepsis study? [yes/no]
28. Did you know that your facility participated in an awareness campaign for the global maternal sepsis study? [yes/no]

IV. Personal information

Remember! These data are collected for categorization purposes only. Your information is confidential and you will not be identified in any future publications on this study.

29. Age range [18-25; 26-30; 31-40; 41-50; 51-60; 60+]
30. Gender: [male, female, other]
31. Qualification: [community health worker, midwife, nurse, physician/medical doctor, resident/physician in training, social worker, student, physical therapist, auxiliary nurse, other: please specify].

Brizuela V, *et al. BMJ Open* 2020; 10:e036338. doi: 10.1136/bmjopen-2019-036338

32. Years of work experience in current setting: [years | months]
33. Total years of work experience (since completing your training): [2-digit max]
34. Place of work: [list of countries]
35. Location (of current or main place of work): [urban/rural]
36. Name of facility and address [text entry]
37. Facility type (of current or main place of work): [Clinic, Health centre, Maternity hospital, Regional/Provincial hospital, District hospital, Other hospital, other]
38. Facility management (of current or main place of work): [private, public, social insurance, NGO, other]
39. Did you participate in the World Sepsis Congress Spotlight on Maternal and Neonatal Sepsis (held on 12 September 2017)? [yes/no]

V. Future contacts

We might be interested in contacting you in the future with more information about this study or to get further information about your responses to this survey. Please provide us with your email address below if you agree with this.

- ☐ I agree.
- ☐ I do not agree

Your contact details will be securely stored by the WHO staff person working on the Global Maternal and Neonatal Initiative for one year. You can contact us to modify or suppress your information at any time. To do so, please contact Ms Vanessa Brizuela (brizuelav@who.int)

40. Please type your email address below [text entry]

Thank you very much for participating in this survey! Your responses are extremely valuable to us in our efforts to improve the health of women and babies.

Please visit http://srhr.org/sepsis for more information about the study, the campaign, and maternal sepsis.

Brizuela V, *et al. BMJ Open* 2020; 10:e036338. doi: 10.1136/bmjopen-2019-036338

Appendix 2. Respondent perception of enabling environments for maternal sepsis identification and management at pre- and post-campaign and changes after campaign implementation, limiting to most favourable responses (N=2,188)

	Pre-campaign n/N (%)	Post-campaign n/N (%)	Pre-post cOR‡ [95% CI]¶	Percentage change %
Perception of enabling environment for maternal sepsis identification and management				
Very confident of making right decisions	390/1,155 (33.8)	395/1,033 (38.2)	1.21* [1.05-1.41]	13.2
Resources ***always*** available to make right decisions	443/1,155 (38.4)	386/1,033 (37.4)	0.96 [0.68-1.35]	-2.6
Very supported by facility in making right decisions	503/1,155 (43.6)	438/1,033 (42.4)	0.95 [0.67-1.37]	-2.6

cOR: crude odds ratio; CI: confidence interval; OR calculated clustering at the country level
Percentage change: [(% in post - % in pre)/% in pre]x100
‡Refers to odds ratio between pre- and post-campaign; ¶reference group: pre-campaign*; *p<0.05*

Responses were dichotomized as follows: a 1 was assigned to the most favourable response (i.e., they felt very confident *about being capable of making the right decision) and a 0 to a combination of all the remaining options (i.e.,* somewhat confident, neutral, not very confident *or* not confident at all*)*

Brizuela V, *et al. BMJ Open* 2020; 10:e036338. doi: 10.1136/bmjopen-2019-036338

Appendix 3. Changes in respondent knowledge and perception of enabling environments after campaign implementation among different sub-groups: [1] sample restricted to ≥1 response per facility at pre- and post-campaign; [2] sample restricted to ≥2 responses per facility at pre- and post-campaign; [3] sample restricted to countries with >30 responses at pre- or post-campaign

	**Full sample (N=2,188)**		Restricted sample ≥1 response per facility (N=1,872)		Restricted sample ≥2 responses per facility (N=1,645)		Restricted sample >30 responses per country (N=1,680)	
	**Pre-post OR‡ [95% CI]¶**	**% change**	Pre-post OR‡ [95% CI] ¶	% change	Pre-post OR‡ [95% CI] ¶	% change	Pre-post OR‡ [95% CI] ¶	% change
Knowledge on maternal sepsis								
Have not heard of maternal sepsis	**0.35* [0.18-0.68]**	**-63.4**	0.36* [0.17-0.77]	-61.82	0.45* [0.22-0.93]	-53.58	0.34* [0.15-0.79]	-63.29
Correctly identified the two criteria to define maternal sepsis	**0.67 [0.43-1.03]**	**-29.4**	0.63 [0.39-1.01]	-33.19	0.57* [0.34-0.93]	-38.76	0.57* [0.33-0.98]	-39.46
Correctly identified management of sepsis when maternal sepsis was suspected	**1.76 [0.73-4.21]**	**30.8**	1.58 [0.58-4.30]	24.05	1.53 [0.51-4.62]	21.70	1.70 [0.51-5.72]	24.89
Perception of enabling environment for maternal sepsis identification and management								
Confident of making right decisions	**1.44* [1.01-2.06]**	**7.3**	1.36 [0.97-1.90]	6.39	1.33 [0.94-1.88]	6.34	1.45 [0.95-2.22]	7.99
Resources available to make right decisions	**1.01 [0.68-1.49]**	**0.1**	1.12 [0.76-1.66]	2.32	1.12 [0.72-1.74]	2.23	0.92 [0.57-1.50]	-1.62
Supported by facility in making right decisions	**1.11 [0.80-1.54]**	**2.0**	1.21 [0.84-1.73]	3.44	1.19 [0.81-1.76]	3.45	0.99 [0.69-1.44]	-0.11

OR: odds ratio; CI: confidence interval; percentage change= [(% in post - % in pre)/% in pre]x100

‡Refers to odds ratio between pre- and post-campaign, OR calculated clustering at the country level; ¶reference group pre-campaign; **p<0.05*

Brizuela V, *et al. BMJ Open* 2020; 10:e036338. doi: 10.1136/bmjopen-2019-036338

Appendix 4a. Changes in respondent knowledge after campaign implementation according to respondent and facility characteristics (N= 2,188)

Respondent characteristics	Had not heard about maternal sepsis						Correctly identified the two criteria to define maternal sepsis						Correctly identified management of sepsis when maternal sepsis was suspected					
	Pre-campaign (N=1,144)		*Post-campaign (N=1,021)*		*OR [95% CI] ¶*	*% change*	*Pre-campaign (N=673)*		*Post-campaign (N= 647)*		*OR [95% CI] ¶*	*% change*	*Pre-campaign (N=251)*		*Post-campaign (N=239)*		*OR [95% CI] ¶*	*% change*
	n	%	N	%		%	n	%	n	%		%	n	%	n	%		%
Overall	*95*	*8*	*31*	*3*	*0.35* [0.23-0.52]*	*-63.4*	*109*	*16*	*74*	*11*	*0.67 [0.43-1.03]*	*-29.4*	*114*	*45*	*142*	*59*	*1.76 [0.73-4.21]*	*30.8*
Qualification	*1,141*		*1,013*				*670*		*645*				*250*		*235*			
Nurse/midwife	74	17	16	4	0.18* [0.10-0.33]	-79.2	14	6	8	3	0.48 [0.20-1.14]	-50.3	24	28	41	46	2.13 [0.53-8.47]	61.3
Physician	16	3	10	2	0.77 [0.26-2.31]	-22.6	72	21	55	19	0.87 [0.55-1.38]	-10.3	55	50	75	67	2.06* [1.09-3.91]	35.1
Resident	4	3	4	4	1.36 [0.13-14.31]	34.3	23	27	11	15	0.48 [0.11-2.11]	-44.4	34	63	25	76	1.84 [0.49-6.90]	20.3
Years of experience	*1,098*		*963*				*642*		*607*				*242*		*229*			
<10	20	4	14	3	0.79 [0.28-2.24]	-19.9	58	18	32	11	0.54 [0.28-1.02]	-41.3	67	48	72	58	1.50 [0.40-5.58]	21.2
10-20	36	10	8	3	0.22* [0.10-0.50]	-75.9	28	15	24	12	0.76 [0.44-1.33]	-21.0	31	47	44	66	2.16* [1.04-4.49]	39.8
>20	26	12	4	2	0.17* [0.08-0.38]	-81.0	22	16	16	16	0.95 [0.53-1.69]	-4.4	13	37	21	57	2.22 [0.72-6.87]	52.8
Region	*1,144*		*1,021*				*673*		*647*				*251*		*239*			
Africa	14	6	4	2	0.27* [0.15-0.49]	-71.6	18	15	19	15	1.04 [0.46-2.31]	3.1	30	53	46	69	1.97 [0.95-4.08]	30.5
Asia	11	7	9	5	0.83 [0.25-2.76]	-15.7	7	5	7	6	1.06 [0.42-2.65]	5.7	4	14	8	23	1.85 [0.37-9.21]	65.8
Eastern Mediterranean	58	44	13	8	0.16* [0.09-0.31]	-81.7	2	3	8	9	3.19 [1.04-9.78]	200.0	2	12	10	45	6.25 [0.88-44.14]	286.5
Europe‡	7	5	2	2	0.39 [0.07-2.23]	-59.7	27	38	12	27	0.63* [0.41-0.96]	-27.3	5	12	4	27	1.60 [0.77-3.33]	126.8
Latin America	5	1	3	1	0.72 [0.07-7.77]	-27.9	55	20	28	11	0.49 [0.22-1.08]	-45.8	73	12	74	74	1.87 [1.17-20.42]	529.3

Country implemented an expanded version of campaign	*1,144*		*1,021*				*673*		647				251		239			
Yes	74	11	13	3	0.23* [0.16-0.32]	-75.2	75	19	37	12	0.58 [0.32-1.05]	-37	92	53	105	76	2.77 [0.88-8.69]	42
No	21	5	18	3	0.71 [0.35-1.45]	-27.6	34	12	37	11	0.89 [0.52-1.53]	-10	22	28	37	38	1.50 [0.51-4.40]	32
Respondent worked in a level III facility	*1,144*		*1,021*				*673*		*647*				*251*		*239*			
Yes	23	3	16	3	0.91 [0.38-2.18]	-8.5	87	18	58	15	0.80 [0.45-1.41]	-17.1	104	51	108	65	1.77 [0.51-6.10]	27.0
No	72	20	15	4	0.15* [0.08-0.28]	-82.3	22	11	16	6	0.51 [0.25-1.03]	-46.3	10	21	34	47	3.31* [1.54-7.12]	123.6

‡Includes countries in Central Asia (Kazakhstan, Kyrgyzstan, and Tajikistan)

OR: odds ratio, OR calculated clustering at the country level; CI: confidence interval; Percentage change= [(% in post - % in pre)/% in pre]x100

¶Reference group: pre-campaign

Where n represents the frequency and N the denominator (i.e. n= the number of respondents with a specific characteristic who answered correctly, N= the total number of persons who answered that question)

Appendix 4b. Changes in respondent perception of enabling environments after campaign implementation according to respondent and facility characteristics

Respondent characteristic	*Confident of making right decisions*						*Resources available to make right decisions*						*Supported by facility in making right decisions*					
	Pre-campaign (N=1,155)		*Post-campaign (N=1,033)*		*OR [95% CI]¶*	*% change*	*Pre-campaign (N=1,155)*		*Post-campaign (N=1,033)*		*OR [95% CI]¶*	*% change*	*Pre-campaign (N=1,155)*		*Post-campaign (N=1,033)*		*OR [95% CI]¶*	*% change*
	n	*%*	*n*	*%*		*%*	*n*	*%*	*n*	*%*		*%*	*N*	*%*	*n*	*%*		*%*
Overall	*897*	*78*	*861*	*83*	*1.44* [1.01-2.06]*	*7.3*	*909*	*79*	*814*	*79*	*1.01 [0.68-1.49]*	*0.1*	*921*	*80*	*840*	*81*	*1.11 [0.80-1.54]*	*2.0*
Qualification	*1,151*		*1,025*				*1,151*		*1,025*				*1,151*		*1,025*			
Nurse/midwife	355	81	371	81	1.05 [0.56-1.96]	0.8	356	81	336	74	0.66 [0.34-1.28]	-8.9	367	83	347	76	0.63* [0.41-0.98]	-8.8
Physician	440	78	395	87	1.78* [1.23-2.57]	10.4	445	79	387	85	1.46* [1.17-1.82]	7.0	448	80	393	86	1.57* [1.12-2.22]	7.9
Resident	100	67	87	77	1.67 [0.97-2.88]	15.5	105	70	86	76	1.37 [0.77-2.42]	8.7	104	69	95	84	2.33* [1.24-4.38]	21.3
Years of experience	*1,107*		*970*				*1,107*		*970*				*1,107*		*970*			
<10	409	76	389	82	1.44 [0.99-2.10]	8.1	416	77	379	80	1.17 [0.80-1.73]	3.6	424	78	390	82	1.25 [0.86-1.83]	4.5
10-20	284	81	264	83	1.08 [0.73-1.59]	1.4	277	79	239	75	0.77 [0.52-1.13]	-5.9	280	80	248	78	0.85 [0.59-1.23]	-3.4
>20	179	82	160	92	2.43* [1.13-5.21]	11.5	187	86	153	88	1.17 [0.72-1.91]	2.0	188	87	158	91	1.52 [0.93-2.49]	4.8
Region	*1,155*		*1,033*				*1,155*		*1,033*				*1,155*		*1,033*			
Africa	189	84	198	88	1.31 [0.76-2.26]	3.8	158	71	157	69	0.95 [0.73-1.24]	-1.5	176	79	173	77	0.89 [0.63-1.26]	-2.6
Asia	128	74	145	85	2.04* [1.17-3.55]	15.3	134	77	150	88	2.18* [1.72-2.76]	13.9	137	79	151	89	2.09* [1.59-2.74]	12.2
Eastern Mediterranean	131	77	115	70	0.70* [0.64-0.77]	-9.0	109	64	84	51	0.59* [0.49-0.71]	-20.1	108	63	97	59	0.83 [0.61-1.14]	-6.9
Europe‡	97	71	77	79	1.59 [0.89-2.82]	12.1	112	82	83	86	1.32 [0.80-2.20]	4.7	112	82	88	91	2.18* [1.54-3.09]	11.0
Latin America	352	78	326	87	1.85* [1.14-3.01]	11.1	396	88	340	91	1.32 [0.68-2.57]	3.0	388	86	331	88	1.20 [0.59-2.44]	2.4

Country implemented an expanded version of campaign	*1,155*		*1,033*				*1,155*		*1,033*				*1,155*		*1,033*			
Yes	531	75	398	80	1.28 [0.76-2.15]	5.7	554	79	375	75	0.82 [0.45-1.48]	-4.6	567	80	391	78	0.87 [0.53-1.43]	-2.8
No	366	81	463	87	1.52* [1.00-2.29]	6.8	355	79	439	82	1.25 [0.83-1.89]	4.4	354	79	449	84	1.45* [1.03-2.05]	7.1
Respondent worked in a level III facility	*1,155*		*1,033*				*1,155*		*1,033*				*1,155*		*1,033*			
Yes	608	77	508	83	1.51* [1.10-2.05]	8.4	646	82	516	85	1.24 [0.85-1.79]	3.6	640	81	518	85	1.33 [0.95-1.87]	5.0
No	289	79	353	83	1.31 [0.77-2.22]	5.1	263	72	298	70	0.92 [0.62-1.36]	-2.5	281	77	322	76	0.94 [0.71-1.25]	-1.4

‡Includes countries in Central Asia (Kazakhstan, Kyrgyzstan, and Tajikistan)

OR: odds ratio, OR calculated clustering at the country level; CI: confidence interval; Percentage change= [(% in post - % in pre)/% in pre]x100

¶Reference group: pre-campaign

Where n represents the frequency and N the denominator (i.e. n= the number of respondents with a specific characteristic who answered correctly, N= the total number of persons who answered that question)
