# Supplementary material for: Early evaluation of the ‘STOP SEPSIS!’ WHO Global Maternal Sepsis Awareness Campaign implemented for healthcare providers in 46 low, middle and high-income countries
Source: BMJ Open. 2020 May 21;10(5):e036338. doi: 10.1136/bmjopen-2019-036338 (PMC7247401; doi:10.1136/bmjopen-2019-036338)
Supplement: Author's manuscript [file bmjopen-2019-036338.draft_revisions.pdf]

BMJ Open is committed to open peer review. As part of this commitment we make the peer review history of every article we publish publicly available.

When an article is published we post the peer reviewers' comments and the authors' responses online. We also post the versions of the paper that were used during peer review. These are the versions that the peer review comments apply to.

The versions of the paper that follow are the versions that were submitted during the peer review process. They are not the versions of record or the final published versions. They should not be cited or distributed as the published version of this manuscript.

BMJ Open is an open access journal and the full, final, typeset and author-corrected version of record of the manuscript is available on our site with no access controls, subscription charges or pay-per-view fees (http://bmjopen.bmj.com).

If you have any questions on BMJ Open's open peer review process please email info.bmjopen@bmj.com

BMJ Open

'STOP SEPSIS!' Evaluation of the WHO Global Maternal Sepsis Awareness Campaign

Journal:	*BMJ Open*
Manuscript ID	bmjopen-2019-036338
Article Type:	Original research
Date Submitted by the Author:	12-Dec-2019
Complete List of Authors:	Brizuela, Vanessa; WHO, UNDP/UNFPA/UNICEF/WHO/World Bank Special Programme of Research, Development and Research Training in Human Reproduction (HRP), Department of Reproductive Health and Research<br>Bonet, Mercedes; World Health Organization, UNDP/UNFPA/UNICEF/WHO/World Bank Special Programme of Research, Development and Research Training in Human Reproduction (HRP), Department of Reproductive Health and Research<br>Trigo Romero, Carla Lionela; University of Sao Paulo Faculty of Medicine of Ribeirao Preto, Department of Social Medicine<br>Abalos, E; Centro Rosarino de Estudios Perinatales<br>Baguiya, Adama; Research Institute of Health Sciences<br>Fawole, Adeniran O.; University of Ibadan College of Medicine<br>Knight, Marian; University of Oxford, National Perinatal Epidemiology Unit<br>Lumbiganon, Pisake; Khon Kaen University, Ob & Gyn<br>Minkauskienė, Meilė; Lithuanian University of Health Sciences, Department of Obstetrics and Gynaecology<br>Nabhan, Ashraf; Ain Shams University, Department of Obstetrics & Gynaecology<br>Osman, Nafissa; Hospital Central de Maputo<br>Qureshi , Zahida ; University of Nairobi Department of Obstetrics and Gynecology,<br>Souza, Joao Paulo; University of Sao Paulo Faculty of Medicine of Ribeirao Preto, Department of Social Medicine
Keywords:	Maternal medicine < OBSTETRICS, PERINATOLOGY, PUBLIC HEALTH

SCHOLARONE™
Manuscripts

I, the Submitting Author has the right to grant and does grant on behalf of all authors of the Work (as defined in the below author licence), an exclusive licence and/or a non-exclusive licence for contributions from authors who are: i) UK Crown employees; ii) where BMJ has agreed a CC-BY licence shall apply, and/or iii) in accordance with the terms applicable for US Federal Government officers or employees acting as part of their official duties; on a worldwide, perpetual, irrevocable, royalty-free basis to BMJ Publishing Group Ltd ("BMJ") its licensees and where the relevant Journal is co-owned by BMJ to the co-owners of the Journal, to publish the Work in this journal and any other BMJ products and to exploit all rights, as set out in our licence.

The Submitting Author accepts and understands that any supply made under these terms is made by BMJ to the Submitting Author unless you are acting as an employee on behalf of your employer or a postgraduate student of an affiliated institution which is paying any applicable article publishing charge ("APC") for Open Access articles. Where the Submitting Author wishes to make the Work available on an Open Access basis (and intends to pay the relevant APC), the terms of reuse of such Open Access shall be governed by a Creative Commons licence – details of these licences and which Creative Commons licence will apply to this Work are set out in our licence referred to above.

Other than as permitted in any relevant BMJ Author's Self Archiving Policies, I confirm this Work has not been accepted for publication elsewhere, is not being considered for publication elsewhere and does not duplicate material already published. I confirm all authors consent to publication of this Work and authorise the granting of this licence.

For peer review only

'STOP SEPSIS!' Evaluation of the WHO Global Maternal Sepsis Awareness Campaign

Authors and affiliations:

Vanessa Brizuela, UNDP/UNFPA/UNICEF/WHO/World Bank Special Programme of Research, Development and Research Training in Human Reproduction (HRP), Department of Reproductive Health and Research, World Health Organization, Avenue Appia 20, 1211 Geneva, Switzerland. Email: brizuelav@who.int. Telephone: +41 22 791 37 40.

Mercedes Bonet, UNDP/UNFPA/UNICEF/WHO/World Bank Special Programme of Research, Development and Research Training in Human Reproduction (HRP), Department of Reproductive Health and Research, World Health Organization, Geneva, Switzerland.

Carla Lionela Trigo Romero, Department of Social Medicine. Ribeirão Preto Medical School, Ribeirão Preto, Brazil.

Edgardo Abalos, Centro Rosarino de Estudios Perinatales (CREP), Rosario, Argentina.

Adama Baguiya, Research Institute for Health Sciences, Ouagadougou, Burkina Faso.

Adeniran Olubukola Fawole†, College of Medicine, University of Ibadan, Ibadan, Nigeria.

Marian Knight, National Perinatal Epidemiology Unit, Nuffield Department of Population Health, University of Oxford, United Kingdom.

Pisake Lumbiganon, Department of Obstetrics and Gynaecology, Faculty of Medicine, Khon Kaen University, Khon Kaen, Thailand.

Meilė Minkauskienė, Department of Obstetrics and Gynaecology, Lithuanian University of Health Sciences, Kaunas, Lithuania.

For peer review only - http://bmjopen.bmj.com/site/about/guidelines.xhtml

Ashraf Nabhan, Department of Obstetrics & Gynaecology, Ain Shams University, Cairo, Egypt.

Nafissa Bique Osman, Faculty of Medicine, Eduardo Mondlane University, Maputo, Mozambique.

Zahida Qureshi, Department of Obstetrics and Gynaecology, University of Nairobi, Nairobi, Kenya.

João Paulo Souza, Department of Social Medicine. Ribeirão Preto Medical School, Ribeirão Preto, Brazil.

On behalf of the WHO GLOSS Research Group

†Deceased January 2019

Correspondence to: Vanessa Brizuela. Email: brizuelav@who.int.

Key words: maternal sepsis, maternal infections, awareness campaign, evaluation, multi-country study

Word count: 3,655

For peer review only - http://bmjopen.bmj.com/site/about/guidelines.xhtml

Abstract (253 words)

Objective: To evaluate changes in awareness of maternal sepsis among healthcare providers resulting from the WHO global maternal sepsis study (GLOSS) awareness campaign.

Design: Independent sample pre-post intervention through online and paper-based surveys. Descriptive statistics were used for campaign recognition and exposure, and odds ratio and percentage change were calculated for differences in awareness, adjusting for confounders using multivariate logistic regression.

Setting and participants: Healthcare providers from 398 participating facilities in 37 low-, middle-, and high-income countries.

Intervention: An awareness campaign to accompany GLOSS launched three weeks prior to data collection and lasting the entire study period (28 November 2017 to 15 January 2018) and beyond.

Main outcome measures: Campaign recognition and exposure, and changes in awareness.

Results: A total of 2,188 surveys were analysed: 1,155 at baseline and 1,033 at post-intervention. Most survey respondents found the campaign materials helpful (94%), that they helped increase awareness (90%), and that they helped motivate to act differently (88%). There were significant changes with regards to: not having heard of maternal sepsis (-63.4% change, pre-post OR 0.35, 95% CI 0.18-0.68) and perception of confidence in making the right decisions with regards to maternal sepsis identification and management (7.3% change, pre-post OR 1.44, 95% CI 1.01-2.06).

Conclusions: Awareness raising campaigns can contribute to an increase in having heard of maternal sepsis and an increase in provider perception of confidence in making correct decisions. Offering the information to make accurate and timely decisions while promoting environments that enable self-confidence and support could improve maternal sepsis identification and management.

For peer review only - http://bmjopen.bmj.com/site/about/guidelines.xhtml

Strengths and limitations of this study

- To the best of our knowledge, this is the first evaluation to describe recognition and exposure as well as changes in awareness from a campaign implemented globally accompanying a research study.
- Awareness campaigns can increase recognition and knowledge of maternal sepsis and improve provider confidence in responding to maternal sepsis, and when accompanying a multi-country study, they can benefit project outcomes.
- This evaluation was a cost-effective, feasible way in which to assess campaign effectiveness among a varied and global population of healthcare providers.
- Our pre-post design methodology with no control group does not allow to discern the impact of the campaign alone.
- Survey dissemination method and anonymity did not allow matching responses at pre- and post-campaign; these methods were chosen to allow for responses from a large, unknown population and to encourage providers to respond honestly.

For peer review only

For peer review only - http://bmjopen.bmj.com/site/about/guidelines.xhtml

INTRODUCTION

The global health community has recently drawn attention to the importance of sepsis and its toll on global mortality and morbidity.[1–3] In 2017, the World Health Assembly approved a resolution on sepsis to improve the prevention, diagnosis, and management of sepsis.[4] With updates in 2017 and 2018, the Surviving Sepsis Campaign has been developing guidelines for management and recommended bundles of care for sepsis among adult populations since 2002.[5–7]

Infections and sepsis remain major causes of death and disability among women during pregnancy, childbirth, postpartum, and post-abortion.[8,9] To respond to this, the Global Maternal Sepsis Initiative was launched in 2016.[4,10] Building on the 2016 SEPSIS-3 definition,[11] the World Health Organization (WHO) led the development of a definition for maternal sepsis as "a life-threatening condition defined as organ dysfunction resulting from infection during pregnancy, childbirth, postpartum, and post-abortion."[12] And in 2017, WHO led the Global Maternal Sepsis Study and Awareness Campaign (GLOSS) to assess the burden of maternal infections and sepsis, to validate identification criteria for possible severe maternal infection and maternal sepsis, and to raise awareness on maternal sepsis among healthcare providers working in study participating facilities.[12]

These initiatives and calls to action all share a recommendation to increase awareness of sepsis among healthcare providers, policy-makers, and the public, in pursuit of reducing the global burden of sepsis.

Awareness raising has mostly been attempted through campaigns. These have been implemented to increase knowledge, improve attitudes, or change behaviours around different health issues.[13–15] Specific to sepsis, the UK Sepsis Trust heads a campaign on sepsis since 2012 and the Global Sepsis Alliance leads efforts aimed at raising sepsis awareness since 2010.[16,17] However, neither of these

For peer review only - http://bmjopen.bmj.com/site/about/guidelines.xhtml

two large campaigns have been specific to maternal sepsis and to our knowledge neither has been thoroughly evaluated to assess for impact in increasing awareness.

This evaluation looked at recognition and exposure to the GLOSS campaign materials and changes in provider awareness of maternal sepsis after campaign implementation. The latter included changes in knowledge on maternal sepsis and perception of enabling environments for identification and management of maternal sepsis.

METHODS

The GLOSS campaign was designed to accompany the Global Maternal Sepsis Study with the goal of raising awareness on maternal sepsis among healthcare providers working in participating facilities. Details regarding study protocol, including selection of countries and facilities was published elsewhere.[12] In short, GLOSS was a facility-based, one-week inception cohort study which enrolled pregnant or recently pregnant women with suspected or confirmed infection at 713 healthcare facilities in pre-specified geographical areas located in 52 low-, middle-, and high-income countries.[12]

The STOP SEPSIS! awareness campaign

The campaign launch was planned for before study implementation continuing throughout data collection and beyond. It was designed using existing frameworks for public information campaigns, social marketing, health communication, and behaviour change.[13,18–20] The development of the campaign included an overarching communication strategy using a multi-component approach delivering a simple and consistent message through visually-attractive media.[21]

The campaign had a soft launch with an online congress on 12 September 2017 and the full campaign rollout began on 06 November 2017, which included a website, printed materials, social media

For peer review only

messaging, press releases. While global coordination of the campaign was undertaken by WHO, implementation of the campaign was the remit of GLOSS country coordinators. **Box 1** describes the different actions and components that were necessary for the design and development of the *STOP SEPSIS! awareness campaign*.

Box 1. Actions and components for the *STOP SEPSIS! awareness campaign*

- *Select a campaign lead.* A campaign lead was selected to coordinate and assist with the development of the campaign strategy and execution, and evaluation plan at a global level. This person ensured execution of each of the steps, supported the communication company and the study country coordinators in the participating countries who interacted with the providers working in the participating facilities.
- *Agree on a budget to fund the campaign*. Funds were necessary to cover the costs of the campaign lead, the communication company, and support to countries for printing of materials. The cost of this campaign was USD 200,000.
- *Seek the assistance of health and media communication experts*. A communication company with expertise in global health was contracted to lead the design and development of the GLOSS campaign concept and look.
- *Decide on the minimum set of materials and activities to be developed and implemented*. With input from people in the field who would be targeted through the campaign, the decision to have posters, infographics, press release and other presentation templates, social media messaging, and a website was agreed upon. In addition, a global congress was conceived in collaboration with partners from the Global Sepsis Alliance.
- *Develop campaign messaging, image, and logo*. A main message, tagline, and logo were designed with assistance from the communication company, content experts in maternal sepsis, and country/regional coordinators for GLOSS.
- *Develop an evaluation plan*. Given the breadth and geographical extent of the campaign's target population an online survey was used to collect providers' knowledge, attitudes, practices at baseline and post-campaign, including additional measures of campaign recognition and exposure at post-campaign. Paper-based surveys were used on demand.
- *Support the printing and upkeep of materials*. The campaign lead coordinated translation of all materials into five UN official languages and three additional languages as per GLOSS country coordinators' request. Participating countries were provided with funds needed to print the posters and infographics. Campaign lead was also in charge of regular upkeep of the dedicated website which includes timely news stories.
- *Implement the campaign*. This included:
    - *WSC Spotlight Congress*. A free, online congress focusing specifically on maternal and neonatal sepsis offered in collaboration with the Global Sepsis Alliance (https://wscspotlight.org/). The 25 presentations given over four sessions were later made available as YouTube videos and podcasts for free, with subtitles in multiple languages.
    - *Website*. A dedicated website used both as a repository of campaign materials for free download and to disseminate news about the study (http://srhr.org/sepsis).
    - *Print materials*. Posters with information about the study and infographics on maternal sepsis prevention, and identification and management to be displayed in

For peer review only - http://bmjopen.bmj.com/site/about/guidelines.xhtml

different areas where women with suspected or confirmed infection could be found (e.g. labour ward, patient waiting area).
  - *Press releases*. Templates for announcing the objectives of the study and the campaign; countries/facilities were encouraged to engage local media for this purpose.
  - *Social media*. Campaign messaging disseminated and multiplied using social media through HRP's Twitter platform (@HRPresearch).
- *Expand the effect of the campaign*. Countries were encouraged to take ownership over the campaign and develop additional materials and organise activities prior to the start of study data collection.

Evaluation of the STOP SEPSIS! awareness campaign

We used an independent sample pre-post intervention design through online and paper-based surveys. Details regarding the definition used for awareness for this campaign, survey formulation and dissemination, including analysis of baseline data have been published elsewhere.[22] Briefly, a pre-campaign 32-question survey was developed to gather baseline information on healthcare providers' awareness of maternal sepsis through self-reported knowledge on maternal sepsis and perception of their work environments as enabling for the identification and management of maternal sepsis. Knowledge was assessed through questions relating to whether respondents had heard of maternal sepsis, correct identification of criteria that define maternal sepsis (infection plus organ dysfunction), and identification of correct initial management of maternal sepsis and infections (antibiotics and fluids) when maternal sepsis was suspected in the case vignette presented in the survey. Perception of enabling environments was assessed through self-reported confidence in making right decisions, reported availability of resources for correct identification and management, and feeling of support from their work environments in dealing with maternal sepsis, using a five-point Likert-scale. The same survey was administered at post-campaign to assess changes in knowledge and perception of their environments; 14 additional questions were included in the post-survey which considered respondents' recognition of and exposure to the campaign, such as knowledge about the study and the campaign, message recall, engagement with social media for the

For peer review only - http://bmjopen.bmj.com/site/about/guidelines.xhtml

campaign, and whether the campaign materials prompted changes in behaviour. See **Appendix 1** for a copy of the surveys.

Eligible respondents were healthcare providers working in GLOSS participating facilities in countries that received financial support for campaign implementation (N=46); we excluded all surveys from respondents that did not explicitly state that they were providers caring for women with infections in healthcare facilities (e.g., hospital administrators, physical therapists, or community health workers, or if the field was left blank) and from countries with less than two responses at either pre- or post-campaign (N=9). See **Figure 1** for a map of all the countries included in GLOSS and in this evaluation. The surveys were distributed using a snowballing technique and were available in eight languages: Arabic, English, French, Italian, Portuguese, Russian, Spanish, and Vietnamese. The surveys were available for over 30 days (pre-campaign between 29 September and 05 November 2017, post-campaign between 31 January and 11 March 2018). Weekly reminders were sent through the online tool and via email to non-respondents. Targeted outreach was undertaken in countries with fewer than two responses. The campaign was active between 06 November 2017 and 15 January 2018; however, countries were encouraged to continue to use the materials beyond GLOSS study implementation. Ethical approval for GLOSS and the awareness campaign was obtained from WHO's Ethics Review Committee (protocol ID A65787) and from local and facility ethics committees as necessary.

Data analysis

We used descriptive analysis to provide frequencies and percentages for the characteristics of the sample, knowledge and perceived enabling environments, and for all the questions relating to campaign recognition and exposure. The latter was assessed through post-campaign surveys only and complemented with self-reported accounts by GLOSS country coordinators. Text-based responses were codified into numerical values according to common emerging themes. All Likert-

For peer review only - http://bmjopen.bmj.com/site/about/guidelines.xhtml

scale answers were dichotomized assigning a 1 to the two most favourable responses (i.e., they felt *very* or *somewhat* confident about making the right decision) and 0 to the combination of remaining options (*neutral*, *not very confident* or *not confident at all*). While previously we assessed dichotomization using 1 to the single most favourable response (i.e., respondent felt *very confident*) and a 0 to the combination of remaining options (*somewhat confident*, *neutral*, *not very confident* or *not confident at all*)[22] we decided to include a more flexible definition of confidence, perception of availability of resources, and feeling of support to allow for a more robust denominator that would enable comparisons. See **Appendix 2** for results of the overall analysis using this second dichotomization not used in this evaluation.

To assess impact of the campaign we conducted several analyses. First, we calculated percentage change ([(% in post - % in pre)/% in pre]x100) and estimated odds ratios (ORs) to determine differences in respondent knowledge and perception of enabling environments after campaign implementation relative to baseline measure for the total sample and by respondent characteristics. Due to the methodology used for survey dissemination and anonymity of surveys, this was not a matched sample, paired response pre-post analysis. Sensitivity analyses were conducted restricting the population to facilities from which we received at least one survey response and at least two responses, and to countries with more than 30 responses per country at pre- and at post-campaign survey.

Second, we used multivariate logistic regression models to explore the association between respondents' and facilities' characteristics at pre- and post-campaign and change in components of awareness after campaign implementation. Based on analysis of baseline data and our assumptions on characteristics that would be associated with levels of awareness,[22] we included the following variables in the model: whether respondent was a physician, years of work experience, region where the respondent worked, whether the country had implemented an *expanded* version of the campaign,

For peer review only

For peer review only - http://bmjopen.bmj.com/site/about/guidelines.xhtml

and whether the facility was a level III facility. Countries were considered to have implemented an expanded version of the campaign if they had not only printed and displayed all posters and infographics, prepared and disseminated press releases, but if they had also organized other activities or developed other materials for the campaign. Since less than 20% of respondents participated in the World Sepsis Congress Spotlight we did not include this variable in our models. We looked at effect modification by examining interactions between the time of the survey (pre- or post-) and each of the characteristics included in the model.

We used Pearson's χ^2 test to compare proportions and Wald's test to assess for significant differences in the models including interaction terms. Logistic regression analysis was used to estimate odds ratios between pre- and post-, crude and adjusted, clustering at the geographical area level. Statistical significance is reported at $p<0.05$. Stata (version 14.2, College Station, TX) was used for the analyses.

Patient and public involvement

This research was done without patient or public involvement. While the development of the campaign was done with input from study regional and country coordinators, respondents to the surveys were not invited to comment on the study design or to contribute to the writing of this manuscript given their anonymity.

Role of the funding source

The funders of the study had no role in study design, data collection, data analysis, data interpretation, or writing of this original article.

RESULTS

A total of 2,188 surveys met our inclusion criteria. Of these, 1,155 from 192 facilities were received at baseline and 1,033 from 196 facilities at post-campaign. There were no significant differences in

For peer review only - http://bmjopen.bmj.com/site/about/guidelines.xhtml

176 sociodemographic characteristics between respondents at pre- and post-campaign surveys, except for a higher proportion of respondents working in a public facility at post-campaign and the higher proportion of respondents from countries where an expanded version of the campaign was implemented (**Table 1**). Responses came from the same overall countries at pre- and post-campaign. Because of the technique used for survey dissemination and because we did not know the total population of potentially exposed healthcare providers working in GLOSS participating facilities (provider turnover, rotation, and replacements is high), we were unable to calculate a response rate. However, since the campaign was implemented equally at the geographical area level, if providers remained within the study area they would have been exposed to the campaign. Results from the sensitivity analyses showed that overall findings in the sub-groups considered were consistent with the results from the complete sample (**Appendix 3**); for this reason, we used the entire sample for all subsequent analyses.

Table 1. Characteristics of respondents and the facilities in which they work at pre- and post-campaign survey (N=2,188)

Respondent characteristics	Pre-campaign (N=1,155)		Post-campaign (N= 1,033)	
	N	%	N	%
Age (in years)	*1,147*		*1,020*	
<31	354	31	301	30
31-40	389	34	407	40
>40	404	35	312	31
Sex	*1,153*		*1,022*	
Male	287	25	223	22
Female	866	75	799	78
Qualification	*1,151*		*1,025*	
Nurse/auxiliary nurse/midwife	440	38	456	44
Physician	561	49	456	44
Resident	150	13	113	11
Years of work experience	*1,107*		*970*	
<10	541	49	476	49
10-20	349	32	320	33
>20	217	20	174	18
Region	*1,155*		*1,033*	
Africa	224	19	226	22

For peer review only - http://bmjopen.bmj.com/site/about/guidelines.xhtml

Asia		173	15		170	16
Eastern Mediterranean		171	15		165	16
Europe‡		137	12		97	9
Latin America		450	39		375	36
Level of the facility in which respondent works	*1,153*			*1,033*		
I		127	11		166	16
II		236	20		258	25
III		790	69		609	59
*Respondent worked in a public facility**	*1,154*			*1,033*		
Yes		937	81		928	90
No		217	19		105	10
*Country implemented an expanded version of campaign**	*1,155*			*1,033*		
Yes		705	39		533	52
No		450	61		500	48

‡Includes countries in Central Asia (Kazakhstan, Kyrgyzstan, and Tajikistan), in line with WHO regions

**p<0.05*

We first present the results relating to campaign recognition and exposure and then results relating to changes in knowledge and perception of respondents' work environments.

Campaign recognition and exposure

Campaign recognition and exposure were high among most of the post-campaign survey respondents. Seventy-six percent of respondents stated they noticed the materials in their facilities; among those, 94% reported finding the materials helpful, 90% that the materials helped increase awareness on maternal sepsis and 88% that the materials motivated them to do something differently. Only 8% of respondents had used Twitter to amplify the message of the campaign (**Figure 2**). Among respondents that stated that the information provided in the materials motivated them to do something differently than before, 83% stated that it motivated them to suspect maternal sepsis and 77% to act fast. (**Figure 3**). Among respondents stating that the materials had not motivated them to

For peer review only

For peer review only - http://bmjopen.bmj.com/site/about/guidelines.xhtml

do anything differently, 45% said it was because they already knew about maternal sepsis identification and management while 12% stated they had not seen the campaign materials.

Country coordinators shared anecdotal experiences of increased awareness in their facilities and the implementation of changes in practice and policies because of the study and the campaign. These accounts speak of a broader engagement with maternal sepsis identification and management. See **Box 2** for some examples.

For peer review only

Box 2. Accounts from the field*

Implementation of the campaign changed the way the city's providers acted. First, it helped in bridging the gap between academics and providers, which, in turn, helped motivate the entire staff around the study. The campaign helped us all feel more committed with the study. And, most importantly, it helped shed light on a problem (maternal sepsis) that we hadn't made public before. (Cali, Colombia)

In (our) facility there was already a protocol for sepsis early recognition, but the campaign, as well as the study made it come alive again. Sepsis was on everyone's eyes and mouths. The teams were very permeable to knowledge and eager to recognize and treat sepsis immediately. (Campinas, Brazil)

Participation in the campaign allowed me to see that we can find cases of maternal sepsis in the most diverse locations in a facility. And that invariably the most complex cases were those resulting from a condition that was neglected or treated incorrectly/untimely. (Maputo, Mozambique)

Despite having some protocols in place, during the campaign and study we realized that these were not sufficient to detect women with infection. This campaign was very important and helped us find a lot of cases that might have been missed otherwise (...) We are planning on improving reporting mechanisms of any suspected cases and supportive supervision and surveillance as a result of this study. (Ulaanbaatar, Mongolia)

As a result of our participation in GLOSS, we actually committed as a Program in our 2017 Maternal Death Review Forum to eliminate maternal sepsis as a cause of maternal death. (Manila, Philippines)

(Since implementing the GLOSS awareness campaign at a national level, we noticed that) we have prioritized the identification and suspicion of maternal and neonatal sepsis in all level I facilities, in specialized hospital care, and in the public health agenda. (Mexico City, Mexico)

* These reports first appeared in a blog post on the Merck for Mothers website in April 2018: https://www.msdformothers.com/blog/assessing-addressing-maternal-sepsis.html and in a news story on WHO/HRP's website in September 2018: https://www.who.int/reproductivehealth/maternal-sepsis-mexico/en/

For peer review only - http://bmjopen.bmj.com/site/about/guidelines.xhtml

Knowledge on maternal sepsis and perception of enabling environments

At pre-campaign survey, 92% of respondents (1,049/1,144) had heard of maternal sepsis. However, only 16% (109/673) of respondents were able to correctly identify the definition criteria of maternal sepsis and 45% (114/251) identified the correct management for maternal sepsis. In addition, at pre-campaign, most survey respondents stated that their work environments were enabling for maternal sepsis identification and management: 78% (897/1,155) stated that they felt confident of making right decisions, 79% (909/1,155) that they perceived resources were available, and 80% (921/1,155) that they felt supported by their facilities. See **Table 2** for overall results.

After campaign implementation there was a significant decrease in respondents who stated not having heard of maternal sepsis (-63.4% change; OR 0.35, 95% CI 0.18-0.68). There was also a significant increase in perceived confidence in making right decisions with regards to maternal sepsis identification and management (7.3% change; OR 1.44, 95% CI 1.01-2.06), although this level was quite high at pre-campaign (78%). There was a slight increase in respondents' ability to identify the correct management when maternal sepsis was suspected after the implementation of the campaign, but this was not statistically significant (30.8% change; OR 1.76, 95% CI 0.73-4.21). See **Appendix 4a and 4b** for these results according to respondent and facility characteristics.

Table 2. Respondent knowledge on maternal sepsis and perception of enabling environments for maternal sepsis identification and management at pre- and post-campaign and changes after campaign implementation (N=2,188)

	Pre-campaign n/N (%)	Post-campaign n/N (%)	Pre-post cOR‡ [95% CI]¶	Percentage change %
Knowledge on maternal sepsis				
Had not heard of maternal sepsis[A]	95/1,144 (8.3)	31/1,021 (3.0)	0.35* [0.18-0.68]	-63.4
Correctly identified the two criteria to define maternal sepsis[B]	109/673 (16.2)	74/647 (11. 4)	0.67 [0.43-1.17]	-29.4
Correctly identified management of sepsis when maternal sepsis was suspected[C]	114/251 (45.4)	142/239 (59. 4)	1.76 [0.73-4.21]	30.8

For peer review only - http://bmjopen.bmj.com/site/about/guidelines.xhtml

Perception of enabling environment for maternal sepsis identification and management				
Confident of making right decisions	897/1,155 (77.7)	861/1,033 (83.4)	1.44* [1.01-2.06]	7.3
Resources available to make right decisions	909/1,155 (78.7)	814/1,033 (78.8)	1.01 [0.68-1.49]	0.1
Supported by facility in making right decisions	921/1,155 (79.7)	840/1,033 (81. 3)	1.11 [0.80-1.54]	2.0

cOR: crude odds ratio; CI: confidence interval
Percentage change: [(% in post - % in pre)/% in pre]x100
‡Refers to odds ratio between pre- and post-campaign; OR calculated clustering at the geographical area level
¶reference group: pre-campaign; **p<0.05*

(A)Responded NO to the question "have you ever heard of the term maternal sepsis?"
(B) Answered INFECTION and ORGAN DYSFUNCTION to the question: "what two criteria best describe maternal sepsis?"
(C) Answered FLUIDS and ANTIBIOTICS to the question: "what would be the first two things a woman should receive," when the respondent answered INFECTION/SEPSIS to the question: "what would you first think could be causing her to feel this way?"

223

224 After controlling for respondent and facility characteristics, being a physician, having less than 10
225 years of experience, and working in a level III facility were associated with decreased odds of not
226 having heard of maternal sepsis at pre-campaign (**Table 3**). Respondents from facilities that had
227 implemented an expanded version of the campaign were more likely to have heard of maternal sepsis
228 and identify the correct management of maternal sepsis at post-campaign. Respondents with less
229 than 10 years of experience were more likely to have heard of maternal sepsis at pre-campaign, but
230 there were no differences across providers with different years of experience after the campaign.

231 Physicians were more likely to respond that they felt confident in making the right decisions at post-
232 campaign, while being a physician and having more than 20 years of experience had a significant
233 interaction with time of the survey with regards to perception of availability of resources and support
234 from their facilities. At pre- and post-campaign, respondents with 20 years or more of experience
235 were more likely to perceive availability of resources for making right decisions and to feel supported
236 by their facilities and these differences between groups were significant after the campaign (**Table**

For peer review only - http://bmjopen.bmj.com/site/about/guidelines.xhtml

4). No differences in the perception of enabling environments were seen among respondents from facilities that had implemented an expanded version of the campaign.

For peer review only

For peer review only - http://bmjopen.bmj.com/site/about/guidelines.xhtml

Table 3. Knowledge on maternal sepsis adjusted for respondents' characteristics (N=2,188)

	Had not heard about maternal sepsis					Correctly identified the two criteria to define maternal sepsis					Correctly identified management of sepsis when maternal sepsis was suspected				
	Pre-campaign		Post-campaign		Wald's test	Pre-campaign		Post-campaign		Wald's test	Pre-campaign		Post-campaign		Wald's test
Respondent characteristics	aOR	95% CI	aOR	95% CI	*p*	aOR	95% CI	aOR	95% CI	*p*	aOR	95% CI	aOR	95% CI	*p*
Physician	0.29*	0.10-0.85	0.58	0.17-1.93	0.108	1.78	0.80-3.95	3.85*	1.54-9.60	0.175	2.07*	1.24-3.45	2.81*	1.08-7.30	0.583
Years of work experience															
<10	0.50*	0.26-0.96	1.57	0.77-3.18	0.035	1.08	0.60-1.96	0.86	0.55-1.34	0.352	0.86	0.53-1.40	0.61	0.30-1.23	0.775
10-20	1 (ref)		1 (ref)			1 (ref)		1 (ref)			1 (ref)		1 (ref)		
>20	1.21	0.61-2.38	0.73	0.19-2.87		1.17	0.59-2.30	1.50*	1.02-2.21		1.59	0.60-4.22	0.9	0.30-2.71	
Country implemented an expanded version of campaign	1.54	0.46-5.12	0.21*	0.06-0.78	0.004	1.18	0.53-2.65	0.86	0.31-2.34	0.281	2.78*	1.01-7.59	8.02*	2.03-31.73	0.437
Respondent worked in a level III facility	0.45*	0.21-0.96	1.79	0.59-5.42	0.006	1.63	0.77-3.45	2.80*	1.32-5.94	0.314	2.10	0.85-5.16	1.14	0.43-3.02	0.406

‡Includes countries in Central Asia (Kazakhstan, Kyrgyzstan, and Tajikistan), in line with WHO regions

Countries were considered to have implemented an expanded version of the campaign if they had not only printed and displayed all posters and infographics, prepared and disseminated press releases, but if they had also organized other activities or developed other materials for the campaign.

Adjusting for whether respondent was a physician, years of work experience, region, whether the country implemented an expanded version of the campaign, and whether respondent worked in a level III facility, clustering at the geographical area level

*aOR: adjusted odds ratio; CI: confidence interval; Wald's test used to assess for differences in the models including interaction terms; *p<0.05*

For peer review only

For peer review only - http://bmjopen.bmj.com/site/about/guidelines.xhtml

Table 4. Perception of enabling environments for maternal sepsis identification and management adjusted for respondents' characteristics (N=2,188)

	Confident of making right decisions					Resources available to make right decisions					Supported by facility in making right decision				
	Pre-campaign		Post-campaign		Wald's test	Pre-campaign		Post-campaign		Wald's test	Pre-campaign		Post-campaign		Wald's test
Respondent characteristics	aOR	95% CI	aOR	95% CI	*p*	aOR	95% CI	aOR	95% CI	*p*	aOR	95% CI	aOR	95% CI	*p*
Physician	1.02	0.64-1.64	1.69*	1.19-2.40	0.246	0.81	0.55-1.20	1.39	0.99-1.95	0.021	0.78	0.55-1.09	1.26	0.83-1.92	0.017
Years of work experience															
<10	0.74	0.51-1.09	0.88	0.63-1.22	0.025	0.63*	0.41-0.97	0.82	0.53-1.27	0.014	0.70	0.46-1.06	0.95	0.69-1.30	0.038
10-20	1 (ref)		1 (ref)			1 (ref)		1 (ref)			1 (ref)		1 (ref)		
>20	1.20	0.76-1.91	2.54*	1.38-4.64		1.66*	1.08-2.54	2.48*	1.49-4.13		1.60*	1.07-2.40	2.78*	1.84-4.20	
Country implemented an expanded version of campaign	0.65	0.36-1.16	0.91	0.56-1.48	0.605	1.00	0.52-1.94	1.57	0.85-2.88	0.241	1.32	0.86-2.03	1.37	0.90-2.07	0.092
Respondent worked in a level III facility	0.90	0.55-1.47	0.64*	0.41-1.00	0.581	1.39	0.86-2.23	1.27	0.79-2.04	0.255	0.88	0.53-1.46	1.18	0.67-2.07	0.116

[‡]Includes countries in Central Asia (Kazakhstan, Kyrgyzstan, and Tajikistan), in line with WHO regions

Countries were considered to have implemented an expanded version of the campaign if they had not only printed and displayed all posters and infographics, prepared and disseminated press releases, but if they had also organized other activities or developed other materials for the campaign.

Adjusting for whether respondent was a physician, years of work experience, region, whether the country implemented an expanded version of the campaign, and whether respondent worked in a level III facility, clustering at the geographical area level

*aOR: adjusted odds ratio; CI: confidence interval; Wald's test used to assess for differences in the models including interaction terms; *p<0.05*

For peer review only

For peer review only - http://bmjopen.bmj.com/site/about/guidelines.xhtml

DISCUSSION

To the best of our knowledge, this is the first study to assess impact of an awareness campaign aimed at healthcare providers and implemented at a global level where pre-campaign and post-campaign data were collected in addition to measures relating to campaign recognition and exposure. Most healthcare providers stated that the campaign helped increase awareness of maternal sepsis and motivated them to do something differently, particularly to suspect maternal sepsis and act faster. Reports from the field also support this finding that exposure to the campaign increased sensitization to maternal infections and sepsis. Moreover, most survey respondents had heard of maternal sepsis even before campaign implementation; after the campaign this increased significantly. Although most respondents perceived their enabling environments in a positive way before campaign implementation, there was an increase in respondent confidence to make the right decisions regarding maternal sepsis identification and management after campaign implementation.

The *STOP SEPSIS! awareness campaign* implementation was effective with regards to respondents' recognition of and exposure to the campaign; other campaign evaluations have used these measures to positively assess short-term impact of campaigns.[23–25] Furthermore, consistent and repeat exposure to campaign messaging have shown to increase awareness;[13] while exposure was only measured over the course of this evaluation period corresponding to the intended implementation period of the campaign, the fact that most respondents stated the campaign raised awareness is a promising trend in the right direction.

Overall knowledge about maternal sepsis increased from pre- to post-campaign implementation among respondents to our survey with regards to having heard about maternal sepsis. Our finding that overall knowledge increased is supported by existing literature that suggests that campaigns can increase knowledge on a specific topic among healthcare providers[14,26,27] as well as among the general population.[23,28,29] The fact that there was a slight increase in identifying the correct

For peer review only - http://bmjopen.bmj.com/site/about/guidelines.xhtml

management of maternal sepsis is important. Research has shown that knowing what is needed to manage maternal sepsis correctly and early management of maternal sepsis are critical to implementing any changes in providers' behaviour and improving maternal health outcomes.[6,30,31] The GLOSS awareness campaign was associated with reducing differences among groups of healthcare providers depending on their qualifications or years of experience. This speaks to the importance of including healthcare providers with different qualifications and years of experience in awareness-raising efforts.

We found there were overall increases in respondent confidence in making right decisions about maternal sepsis identification and management, but no significant changes with regards to overall respondent perception of availability of necessary resources and feeling supported by their facilities. Evidence shows that confidence can not only affect clinical performance;[32] but also that high levels of confidence among healthcare providers can have a positive impact on patients' perception of experience of care.[33] However, the change in perception of availability of resources and support limited to physicians and more experienced providers raises a broader question on actions that facilities need to take to empower all healthcare workers in feeling that they have the necessary resources and feel supported to provide quality care. This is especially important if we consider that a more restrictive definition of enabling environments results in much lower overall levels of perceived confidence, perception of availability of resources, and feeling of support. These findings are a call to hospital administrators and policy-makers to foster enabling environments and secure availability and access to life-saving resources.

Sepsis awareness is gaining traction on global agendas;[4,10,16] this is supported by evidence from two studies looking at internet searches on sepsis,[34,35] meaning increases resulting from this campaign could be responding to natural trends or other factors. It is also possible that awareness was raised by having participated in the research study and not necessarily because of the campaign;

For peer review only - http://bmjopen.bmj.com/site/about/guidelines.xhtml

disentangling the effect of the campaign from that of the implementation of the research study was impossible. Understanding whether any of these changes are sustained over time would provide us with further information on the lasting effects of the campaign.

Literature shows that while a campaign can help in raising awareness, it is insufficient in allowing for changes in behaviour.[13,19] While behaviour change is important in impacting population-level health, it is one of many components needed to make significant improvements; evidence from this study, similar to others, highlight the need for health systems improvements such as availability of critical resources and support to improve maternal outcomes.[36] Assessing the impact that increased awareness resulting from a campaign has on behaviour change would provide us with supporting evidence that campaigns can help in improving health outcomes.

This study has some limitations. First, we used a pre-post design methodology with no control group which does not allow to discern the impact of the campaign alone. Second, the method used to disseminate the survey and the fact that surveys were anonymous made it impossible to match responses at pre- and post-campaign. Surveys were anonymous to encourage providers to respond and remove potential response bias. However, it is to note that characteristics of participants at pre- and post-campaign were similar. Third, because implementation of the campaign was left up to country coordinators, campaign fidelity was only assessed through healthcare provider self-report at post-campaign surveys. Fourth, this evaluation was restricted to the duration of the study follow-up period, limiting our knowledge of lasting impact of the campaign, which was beyond the goal of this activity. However, our findings suggest that campaigns can have at least short-term effects on provider's knowledge and confidence. The positive perception of the campaign materials is encouraging. And fifth, since baseline data was collected after the soft launch of the campaign, the effect of the campaign may have been minimized because awareness had already been increased

For peer review only - http://bmjopen.bmj.com/site/about/guidelines.xhtml

314 through exposure to the online congress as well as other global activities on sepsis conducted by
315 other groups. However, we know that less than 20% of respondents participated in the congress.

316 Our findings have implications for both practice and research. On the one hand, there appear to be
317 benefits to coupling large multi-country studies with awareness campaigns. A campaign targeting
318 healthcare providers can promote their engagement with research studies being conducted,
319 potentially improving study outcomes. There is also evidence that including an awareness campaign
320 creates an environment prime to implementing changes to clinical practice as per research study
321 protocol. On the other hand, there is a clear need for additional research to identify lasting effects of
322 awareness campaigns, especially as global initiatives focus on increasing awareness on maternal
323 health issues.

324 A campaign designed to raise awareness among healthcare providers working in facilities
325 participating in a global research study was associated with an increase in having heard of maternal
326 sepsis, as well as increased provider perception of confidence in making correct decisions. Offering
327 healthcare providers with the information to make accurate and timely decisions while promoting
328 environments that enable self-confidence and support could improve maternal sepsis identification
329 and management, which can ultimately have an impact on maternal health outcomes.

330

331 **FIGURES**

332 **Figure 1**. Countries participating in GLOSS (N=52)

333 **Figure 2**. Measures of campaign exposure in percentages (N=1,033)

For peer review only - http://bmjopen.bmj.com/site/about/guidelines.xhtml

Figure 3. Responses when answering YES to the question "Did the information provided in the materials motivate you to do something differently than before?" (N=668). (Respondents were able to check as many response options as needed)

APPENDICES

Appendix 1. Global Maternal Sepsis Study – Pre- and Post- Campaign Surveys

Appendix 2. Respondent perception of enabling environments for maternal sepsis identification and management at pre- and post-campaign and changes after campaign implementation, limiting to most favourable responses (N=2,188)

Appendix 3. Changes in respondent knowledge and perception of enabling environments after campaign implementation among different sub-groups

Appendix 4. Changes in respondent knowledge and perception of enabling environments after campaign implementation according to respondent and facility characteristics

SUPPLEMENTARY FILES

Supplementary file 1. Published protocol for the Global Maternal Sepsis Study and Awareness Campaign

Supplementary file 2. Strobe checklist

Acknowledgments: The authors wish to thank Khalid Yunis for his contributions to the development of the campaign, Soe Soe Thwin and Cristina Cuesta for their assistance in the planning and review of statistical analyses, and all the respondents to the online and paper-based survey. The authors

For peer review only

would also like to acknowledge the contribution and lifelong achievements of co-author Bukola Fawole, who passed away prior to the publication of this article.

Contributors: The evaluation plan and first draft of this original article was conceived by VB. MB provided substantial contributions to the data planning and analysis and first versions of this manuscript. The idea for the inclusion of an awareness campaign was first conceived by JPS. CLTR, JPS, and EA provided additional feedback on first draft and data analysis. All authors read and approved the final version. VB, MB, and CLTR had full access to all the data and VB has final responsibility for the decision to submit for publication.

Funding: This work was supported by the UNDP/UNFPA/UNICEF/WHO/World Bank Special Programme of Research, Development and Research Training in Human Reproduction (HRP), Department of Reproductive Health and Research, World Health Organization, Geneva, Switzerland (project A65787), Merck Sharp & Dohme Corp., a wholly owned subsidiary of Merck and Co., Inc. (Kenilworth, NJ USA), through its Merck for Mothers program, and the United States Agency for International Development (USAID) Grant number GHA-G-00-09-00003. The views of the funding bodies have not influenced the content of this manuscript.

Competing interests: All authors have completed the ICMJE uniform disclosure form at www.icmje.org/coi_disclosure.pdf and declare: support from WHO/RHR, Merck for Mothers, and USAID for the submitted work; VB, MB, and JPS were employed by WHO at the time of the study; no other relationships or activities that could appear to have influenced the submitted work.

For peer review only

Ethical approval: Ethical approval for the entire study, including the awareness campaign, was obtained from WHO's Ethics Review Committee in Geneva, Switzerland (protocol ID A65787) for a multi-site, multi-country project. A legend was included in the survey stating that response to the survey implied consent to participate; respondents could recuse themselves at any point during the survey, including skipping and not answering certain questions.

Data sharing: All enquiries regarding the data and analyses can be made to the corresponding author.

Transparency: The lead author (VB) affirms that the manuscript is an honest, accurate, and transparent account of the study being reported; that no important aspects of the study have been omitted; and that any discrepancies from the study as planned have been explained.

Disclosure: This article represents the views of the named authors only and does not represent the views of the World Health Organization

World Health Organization Global Maternal Sepsis Study Research Group (in alphabetical order)

AFGHANISTAN: Mohammad Iqbal Aman, Bashir Noormal. ARGENTINA: Virginia Díaz, Marisa Espinoza, Julia Pasquale. BELGIUM: Charlotte Leroy, Kristien Roelens, Griet Vandenberghe. BENIN: M. Christian Urlyss Agossou, Sourou Goufodji Keke, Christiane Tshabu Aguemon. BOLIVIA: Patricia Soledad Apaza Peralta, Víctor Conde Altamirano, Rosalinda Hernández Muñoz. BRAZIL: José Guilherme Cecatti, Carolina Ribeiro do Valle. BURKINA FASO: Vincent Batiene, Kadari Cisse, Henri Gautier Ouedraogo. CAMBODIA: Cheang Kannitha, Lam Phirun, Tung Rathavy. CAMEROON: Elie Simo, Pierre-Marie Tebeu, Emah Irene Yakana. COLOMBIA: Javier Carvajal, María Fernanda Escobar,

For peer review only

For peer review only - http://bmjopen.bmj.com/site/about/guidelines.xhtml

Paula Fernández. DENMARK: Lotte Berdiin Colmorn, Jens Langhoff-Roos. ECUADOR: Wilson Mereci, Paola Vélez. EGYPT: Yasser Salah Eldin, Alaa Sultan. ETHIOPIA: Abdulfetah Abdulkadir Abdosh, Alula M. Teklu, Dawit Worku. GHANA: Richard Adanu, Philip Govule, Charles Noora Lwanga. GUATEMALA: William Enrique Arriaga Romero, María Guadalupe Flores Aceituno. HONDURAS: Carolina Bustillo, Rigoberto Castro, Bredy Lara. INDIA: Vijay Kumar, Vanita Suri, Sonia Trikha. ITALY: Irene Cetin, Serena Donati, Carlo Personeni. KAZAKHSTAN: Guldana Baimussanova, Saule Kabylova, Balgyn Sagyndykova. KENYA: George Gwako, Alfred Osoti, Zahida Qureshi. KYRGYZSTAN: Raisa Asylbasheva, Aigul Boobekova, Damira Seksenbaeva. LEBANON: Faysal El Kak, Saad Eddine Itani, Sabina Abou Malham. LITHUANIA: Meilė Minkauskienė, Diana Ramašauskaitė. MALAWI: Owen Chikhwaza, Luis Gadama, Eddie Malunga. MALI: Haoua Dembele, Hamadoun Sangho, Fanta Eliane Zerbo. MEXICO: Filiberto Dávila Serapio, Nazarea Herrera Maldonado, Juan I. Islas Castañeda. REPUBLIC OF MOLDOVA: Tatiana Cauaus, Ala Curteanu, Victor Petrov. MONGOLIA: Yadamsuren Buyanjargal, Seded Khishgee, Bat-Erdene Lkhagvasuren. MOROCCO: Bouchra Assarag, Amina Essolbi, Rachid Moulki. MOZAMBIQUE: Zara Jaze, Arlete Mariano, Nafissa Osman. MYANMAR: Hla Mya Thway Einda, Thae Maung Maung, Khaing Nwe Tin. NEPAL: Tara Gurung, Amir Babu Shrestha, Sangeeta Shrestha. NETHERLANDS: Kitty Bloemenkamp, Marcus J. Rijken, Thomas Van Den Akker. NICARAGUA: María Esther Estrada, Néstor J. Pavón Gómez. NIGERIA: Olubukola Adesina, Chris Aimakhu, Bukola Fawole. PAKISTAN: Rizwana Chaudhri, Saima Hamid, M. Adnan Khan. PERU: María del Pilar Huatuco Hernández, Nelly M. Zavaleta Pimentel. PHILIPPINES: Maria Lu Andal, Zenaida Dy Recidoro, Carolina Paula Martin. ROMANIA: Mihaela Budianu, Lucian Pușcașiu. SENEGAL: Léopold Diouf, Dembo Guirassy, Philippe Marc Moreira. SLOVAKIA: Miroslav Borovsky, Ladislav Kovac, Alexandra Kristufkova. SOUTH AFRICA: Sylvia Cebekhulu, Laura Cornelissen, Priya Soma-Pillay. SPAIN: Vicenç Cararach, Marta López, María José Vidal Benedé. SRI LANKA: Hemali Jayakody, Kapila Jayaratne, Dhammica Rowel. SUDAN: Mohamed Elsheikh, Wisal Nabag, Sara Omer. TAJIKISTAN: Victoria Tsoy, Urunbish Uzakova, Dilrabo Yunusova. THAILAND: Thitiporn Siriwachirachai,

For peer review only

For peer review only - http://bmjopen.bmj.com/site/about/guidelines.xhtml

Thumwadee Tangsiriwatthana. UNITED KINGDOM: Catherine Dunlop, Marian Knight, David Lissauer. URUGUAY: Aquilino M. Pérez, Jhon Roman, Gerardo Vitureira. VIET NAM: Dinh Anh Tuan, Luong Ngoc Truong, Nghiem Thi Xuan Hanh. ZIMBABWE: Mugove Madziyire, Thulani Magwali, Stephen Munjanja. Regional Coordinators: Edgardo Abalos, Adama Baguiya, Mónica Chamillard, Bukola Fawole, Marian Knight, Seni Kouanda, Pisake Lumbiganon, Ashraf Nabhan, Ruta Nadisauskiene. Technical Advisory Group: Linda Bartlett; Fernando Bellissimo-Rodrigues; Shevin T. Jacob; Sadia Shakoor; Khalid Yunis. Data management and analysis: Liana Campodónico, Cristina Cuesta, Hugo Gamerro, Daniel Giordano. WHO coordinating Unit: Fernando Althabe, Mercedes Bonet, Vanessa Brizuela, A. Metin Gülmezoglu, João Paulo Souza.

For peer review only

For peer review only - http://bmjopen.bmj.com/site/about/guidelines.xhtml

References

1 Reinhart K, Daniels R, Kissoon N, *et al.* Recognizing Sepsis as a Global Health Priority — A WHO Resolution. *N Engl J Med* 2017;**377**:414–7. doi:10.1056/NEJMp1707170

2 Rudd KE, Kissoon N, Limmathurotsakul D, *et al.* The global burden of sepsis: barriers and potential solutions. *Crit Care* 2018;**22**. doi:10.1186/s13054-018-2157-z

3 Jawad I, Lukšić I, Rafnsson SB. Assessing available information on the burden of sepsis: global estimates of incidence, prevalence and mortality. *J Glob Health* 2012;**2**. doi:10.7189/jogh.02.010404

4 WHO. Improving the prevention, diagnosis and management of sepsis. World Health Assembly: 2017. http://apps.who.int/gb/ebwha/pdf_files/WHA70/A70_R7-en.pdf?ua=1

5 Surviving Sepsis Campaign | History. http://www.survivingsepsis.org/About-SSC/Pages/History.aspx (accessed 20 Aug 2017).

6 Rhodes A, Evans LE, Alhazzani W, *et al.* Surviving Sepsis Campaign: International Guidelines for Management of Sepsis and Septic Shock: 2016. *Intensive Care Med* 2017;**43**:304–77. doi:10.1007/s00134-017-4683-6

7 Levy MM, Evans LE, Rhodes A. The Surviving Sepsis Campaign Bundle: 2018 update. *Intensive Care Med* 2018;**44**:925–8. doi:10.1007/s00134-018-5085-0

8 Say L, Chou D, Gemmill A, *et al.* Global causes of maternal death: a WHO systematic analysis. *Lancet Glob Health* 2014;**2**:e323–e333.

9 Kassebaum NJ, Barber RM, Bhutta ZA, *et al.* Global, regional, and national levels of maternal mortality, 1990–2015: a systematic analysis for the Global Burden of Disease Study 2015. *The Lancet* 2016;**388**:1775–812. doi:10.1016/S0140-6736(16)31470-2

10 The Global Maternal and Neonatal Sepsis Initiative: a call for collaboration and action by 2030. *Lancet Glob Health* 2017;**0**. doi:10.1016/S2214-109X(17)30020-7

11 Singer M, Deutschman CS, Seymour CW, *et al.* The Third International Consensus Definitions for Sepsis and Septic Shock (Sepsis-3). *JAMA* 2016;**315**:801. doi:10.1001/jama.2016.0287

12 Bonet M, Souza JP, Abalos E, *et al.* The global maternal sepsis study and awareness campaign (GLOSS): study protocol. *Reprod Health* 2018;**15**:16. doi:10.1186/s12978-017-0437-8

13 Randolph W, Viswanath K. Lessons Learned from Public Health Mass Media Campaigns: Marketing Health in a Crowded Media World. *Annu Rev Public Health* 2004;**25**:419–37.

14 Pittet D, Hugonnet S, Harbarth S, *et al.* Effectiveness of a hospital-wide programme to improve compliance with hand hygiene. *The Lancet* 2000;**356**:1307–12. doi:10.1016/S0140-6736(00)02814-2

15 Glanz K, Rimer BK, Lewis, editors. *Health Behavior and Health Education: Theory, Research, and Practice*. 3rd ed. San Francisco: : Jossey-Bass 2002.

For peer review only - http://bmjopen.bmj.com/site/about/guidelines.xhtml

For peer review only

16 Global Sepsis Alliance. https://www.global-sepsis-alliance.org/ (accessed 27 Feb 2019).

17 UK Sepsis Trust. Sepsis Trust. https://sepsistrust.org/ (accessed 27 Feb 2019).

18 Weiss JA, Tschirhart M. Public Information Campaigns as Policy Instruments. *J Policy Anal Manage* 1994;**13**:82. doi:10.2307/3325092

19 Wakefield MA, Loken B, Hornik RC. Use of mass media campaigns to change health behaviour. *The Lancet* 2010;**376**:1261–71. doi:10.1016/S0140-6736(10)60809-4

20 *Making Health Communication Programs Work*. National Cancer Institute 2004. https://www.cancer.gov/publications/health-communication/pink-book.pdf (accessed 11 Jan 2017).

21 O'Connor M, McGowan K, Jolivet RR. An awareness-raising framework for global health networks: lessons learned from a qualitative case study in respectful maternity care. *Reprod Health* 2019;**16**. doi:10.1186/s12978-018-0662-9

22 Brizuela V, Bonet M, Souza JP, *et al.* Factors influencing awareness of healthcare providers on maternal sepsis: a mixed-methods approach. *BMC Public Health* 2019;**19**:683. doi:10.1186/s12889-019-6920-0

23 Kaufman MR, Harman JJ, Smelyanskaya M, *et al.* "Love me, parents!": impact evaluation of a national social and behavioral change communication campaign on maternal health outcomes in Tanzania. *BMC Pregnancy Childbirth* 2017;**17**:305. doi:10.1186/s12884-017-1470-x

24 Evans WD, Uhrig J, Davis K, *et al.* Efficacy methods to evaluate health communication and marketing campaigns. *J Health Commun* 2009;**14**:315–30. doi:10.1080/10810730902872234

25 Bazzo S, Black D, Mitchell K, *et al.* 'Too Young To Drink'. An international communication campaign to raise public awareness of fetal alcohol spectrum disorders. *Public Health* 2017;**142**:111–5. doi:10.1016/j.puhe.2016.08.001

26 Al-Dorzi HM, Cherfan A, Al-Harbi S, *et al.* Knowledge of thromboprophylaxis guidelines pre- and post-didactic lectures during a venous thromboembolism awareness day at a tertiary-care hospital. *Ann Thorac Med* 2013;**8**:165–9. doi:10.4103/1817-1737.114298

27 Pattinson RC, Bergh A-M, Makin J, *et al.* Obstetrics knowledge and skills training as a catalyst for change. *S Afr Med J* 2018;**108**:748. doi:10.7196/SAMJ.2018.v108i9.13073

28 Vallone D, Cantrell J, Bennett M, *et al.* Evidence of the Impact of the truth FinishIt Campaign. *Nicotine Tob Res* 2018;**20**:543–51. doi:10.1093/ntr/ntx119

29 Alaqeel A, Kamalmaz H, Abou Al-Shaar H, *et al.* Evaluating the initial impact of the Riyadh Epilepsy Awareness Campaign. *Epilepsy Behav* 2015;**52**:251–5. doi:10.1016/j.yebeh.2015.09.010

30 Robson W, Beavis S, Spittle N. An audit of ward nurses' knowledge of sepsis. *Nurs Crit Care* 2007;**12**:86–92. doi:10.1111/j.1478-5153.2007.00210.x

For peer review only

31 Gaieski D, Mikkelsen M, Band R, *et al.* Impact of time to antibiotics on survival in patients with severe sepsis or septic shock in whom early goal-directed therapy was initiated in the emergency department*. *Crit Care Med* 2010;**38**:1045–53. doi:10.1097/CCM.0b013e3181cc4824

32 Cheng ER, Nelson CC, Leung-Strle P, *et al.* Nutrition provider confidence in the Massachusetts Childhood Obesity Research Demonstration (MA-CORD) study. *Prev Med Rep* 2019;**13**:289–92. doi:10.1016/j.pmedr.2019.01.010

33 Owens KM, Keller S. Exploring workforce confidence and patient experiences: A quantitative analysis. *Patient Exp J* 2018;**5**:10.

34 Jabaley CS, Blum JM, Groff RF, *et al.* Global trends in the awareness of sepsis: insights from search engine data between 2012 and 2017. *Crit Care Lond Engl* 2018;**22**:7. doi:10.1186/s13054-017-1914-8

35 Savelkoel J, Claushuis TAM, van Engelen TSR, *et al.* Global impact of World Sepsis Day on digital awareness of sepsis: an evaluation using Google Trends. *Crit Care* 2018;**22**. doi:10.1186/s13054-018-1981-5

36 van Den Broek, Nynke, Ameh C, Madaj B, *et al.* Effects of emergency obstetric care training on maternal and perinatal outcomes: a stepped wedge cluster randomised trial in South Africa. *BMJ Glob Health* Published Online First: 2019. doi:10.1136/ bmjgh-2019-001670

For peer review only

For peer review only - http://bmjopen.bmj.com/site/about/guidelines.xhtml

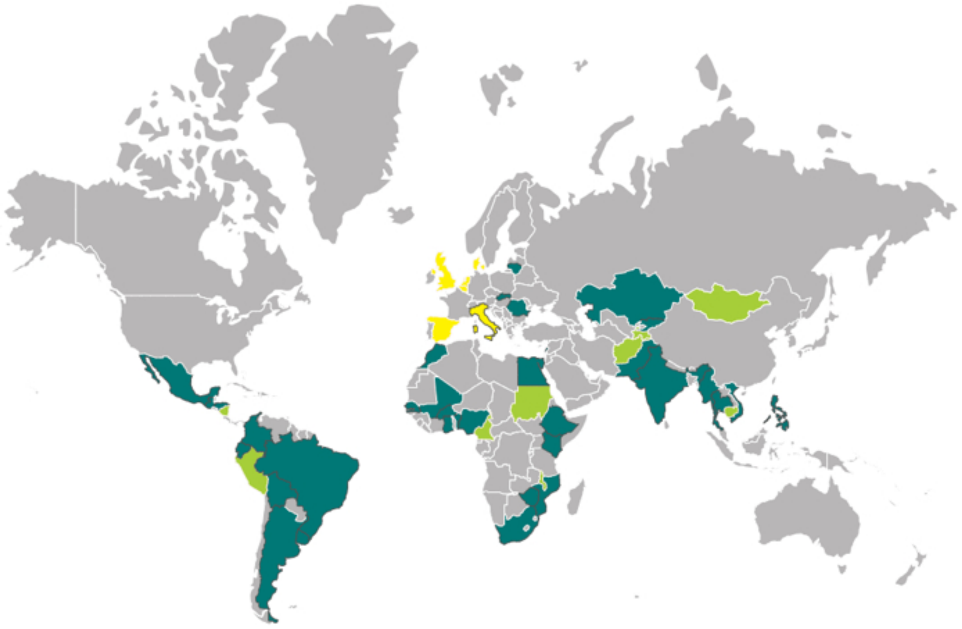

Figure 1. Countries participating in GLOSS (N=52). Key: teal: countries included in the GLOSS STOP SEPSIS! awareness campaign evaluation (N=37); green: countries eligible for the evaluation but from which 1 or less responses received for the evaluation (N=9); yellow: countries that participated in GLOSS but did not implement the awareness campaign (N=6)

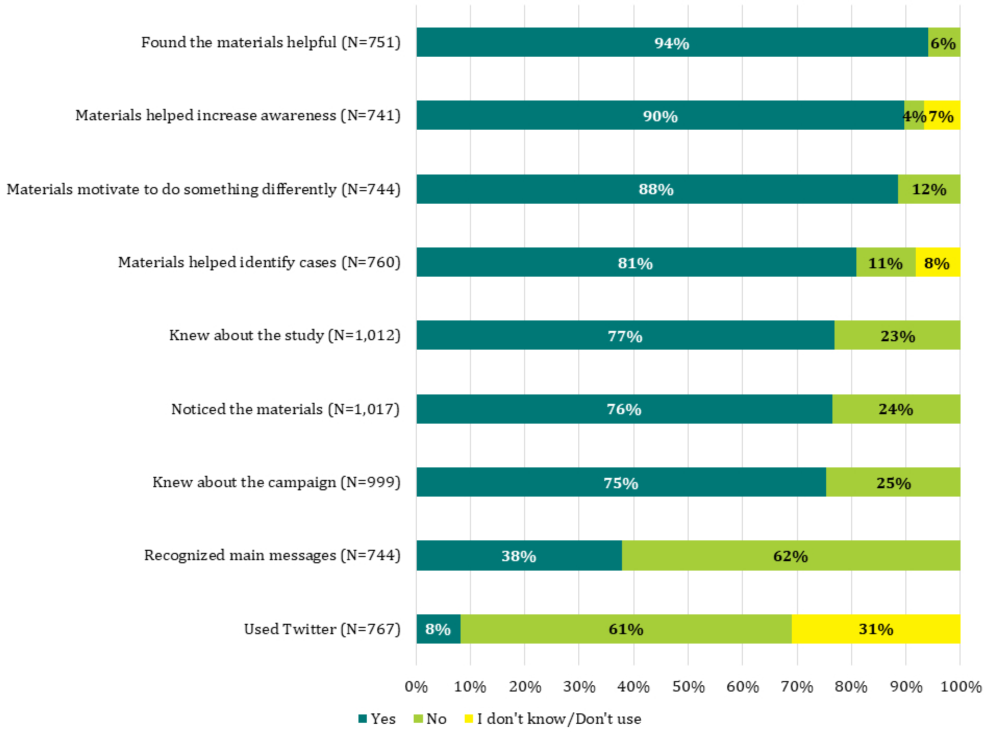

Figure 2. Measures of campaign exposure in percentages (N=1,033)

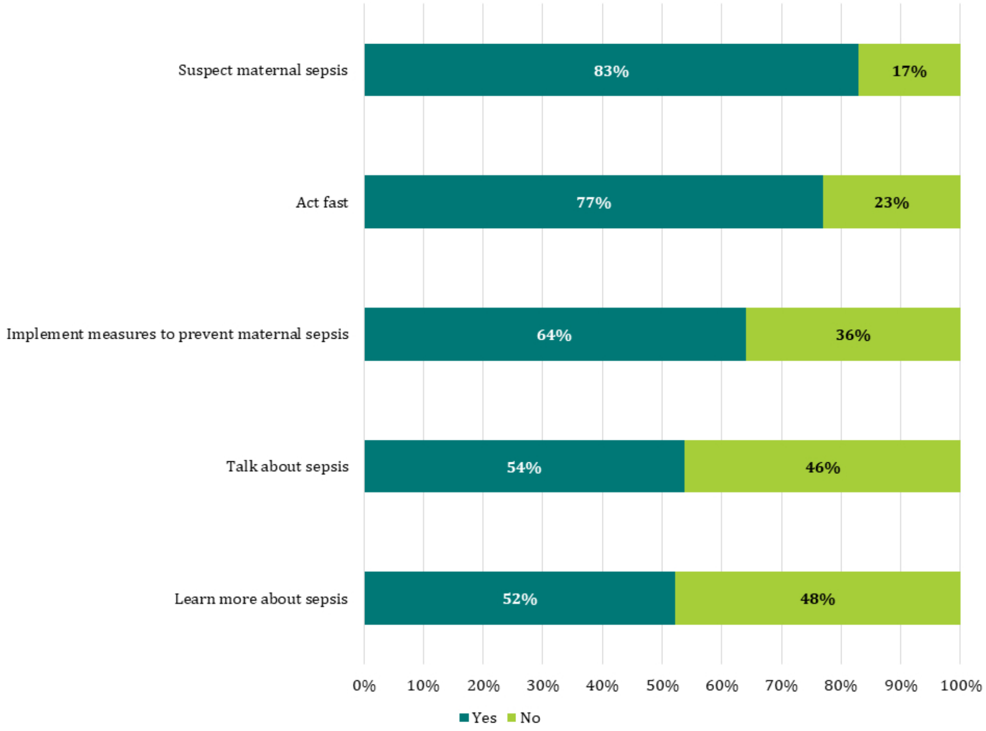

Figure 3. Responses when answering YES to the question "Did the information provided in the materials motivate you to do something differently than before?" (N=668). (Respondents were able to check as many response options as needed)

Appendix 1. Global Maternal Sepsis Study – Pre- and Post-Campaign Surveys

GLOSS Pre-Campaign Survey

This online survey is part of the activities set forth for a global study on maternal morbidity and mortality. This study is being conducted in approximately 50 countries across the globe, including your own, and it is coordinated by the World Health Organization and the healthcare facility where you work.

As part of this study, we want to learn more from healthcare providers about how you identify and manage women with complications during pregnancy, childbirth, postpartum, or post- abortion. The survey includes a number of questions on your knowledge, attitudes, and practices around maternal and neonatal health. This is not a test; this is an opportunity to let us know your thoughts and experience on the topic as a healthcare provider in one of the hospitals participating in the study.

This survey is voluntary, and your answers will be kept confidential, and you can choose whether to leave some questions unanswered. General information about you, your position, and geographical location will be collected to help us categorize respondents only but will not be used to identify you in particular. You are free to provide this information at the end of the survey.

After the study, and only if you agree, a second online survey will be sent to you via email. For this reason, we will ask you to provide an email address so that we can ensure delivery of the second survey. You will be free to decide to participate in this second survey too. Results of these surveys will be published in a peer-reviewed journal without attributing responses to any specific person or institution.

The completion of this survey implies your consent to participate.

If you have any question about the survey, please contact Ms Vanessa Brizuela (brizuelav@who.int)

For peer review only

For peer review only - http://bmjopen.bmj.com/site/about/guidelines.xhtml

I. Knowledge and attitudes

The following questions will ask that you respond according to your current role, competences, and skills depending on your training and background. That is, according to these, you may be the person triaging, prescribing, diagnosing, treating. Bear this in mind when responding.

1. What are the main conditions causing death and disability among women during pregnancy and/or childbirth in your hospital? Check all that apply [abortion-related complications, chronic/pre-existing disease, embolism, haemorrhage, infection/sepsis, pre-eclampsia/eclampsia, other: please specify]
2. Case vignettes:

   Case A: A 25-year-old 32- week pregnant woman comes to your facility brought by a family member saying she is feeling unwell. Her companion reports that she seems a bit disoriented and feverish. Without any further diagnostic testing or triaging:

   a. What would you first think could be causing her to feel this way? Choose from the following list [abortion-related complications, embolism, haemorrhage, infection/sepsis, pre-eclampsia/eclampsia, other]
   b. What would be the first two things this woman should receive? [antibiotics, blood transfusion, body fluid culture, fetal monitoring, fluids, haematology/biochemistry laboratory, other antimicrobials (i.e. antimalarials, ART), other laboratory test, other medication, oxygen, physical exam, urine output measurement, other]
3. Case B: A recently pregnant woman comes to your facility complaining that she has abdominal pain and shortness of breath. Without any further diagnostic testing or triaging:
   a. What would you first think could be causing her to feel this way? Choose from the following list [abortion-related complications, embolism, haemorrhage, infection/sepsis, pre-eclampsia/eclampsia, other]
   b. What would be the first two things this woman should receive? [antibiotics, blood transfusion, body fluid culture, fetal monitoring, fluids, haematology/biochemistry laboratory, other antimicrobials (i.e. antimalarials, ART), other laboratory test, other medication, oxygen, physical exam, urine output measurement, other]
4. How confident do you feel that you are capable of making the right decision in a case like the one above? [very confident, somewhat confident, neutral, not too confident, not confident at all]
5. How would you qualify the availability of resources in the facility where you work to help you make the right decisions? [always available, somewhat available, neutral, not always available, not available at all].
6. How supported do you feel by the facility in which you work to make the right decision in a case like the one above? [very supported, somewhat supported, neutral, not very supported, unsupported].
7. How well does this statement describe your facility: "The facility where I work doesn't let me handle cases like the one described above." [very well, somewhat well, indifferent, somewhat incorrectly, completely incorrectly]

For peer review only - http://bmjopen.bmj.com/site/about/guidelines.xhtml

8. Of the following, which do you think are the greatest barriers in making a right, and timely decision in your facility? Check up to two options. [I'm afraid of making a mistake, I've never seen cases like these, my supervisor doesn't let me make them, not sure I know the correct signs, we don't have a way to triage/treat/manage cases like these in my hospital, other]
9. Does the hospital you work in have protocols in place for dealing with cases like the one described above? [yes/no/don't know]

DO NOT ALLOW GOING BACK AFTER THIS QUESTION

10. Have you ever heard of the term maternal sepsis? [yes/no]
11. If yes, how did you hear about this? Check all that apply [pre-service training, in-service training, public health campaign, colleagues, media (TV/radio/newspaper), other: please specify].
12. What two criteria best describe maternal sepsis? Check two options [abnormal white cell count, altered mental status, elevated heart rate, excessively rapid respiration, fever, infection, low blood pressure, organ dysfunction, other]
13. What supplies/commodities are essential to effectively identify sepsis among women during pregnancy, childbirth, postpartum or post-abortion? Check all that apply [blood culture, blood pressure apparatus, diagnostic imaging, laboratory (haematology/biochemistry), rapid test for infectious disease, serum lactate measurement, thermometer, urine output measurement, other]
14. What supplies/commodities are essential to effectively manage sepsis in women during pregnancy, childbirth, postpartum or post-abortion? Check all that apply [antibiotics, blood transfusions, fluids, intensive care/high-dependency unit, other antimicrobials (e.g. antimalarials, ART), oxygen, urine output measurement, other]

II. Context

15. How many women are affected by maternal sepsis in your facility every year? Give your best estimate (a whole number), given your experience in the facility [Text box]
16. How many neonates are affected by neonatal sepsis in the first week of life in your facility? Give your best estimate (a whole number), given your experience in the facility. [Text box]
17. How many deliveries occur every year, on average, in your facility? Give your best estimate (a whole number). [Text box]
18. Have you ever received specific training in how to manage women who present with signs of infection while pregnant, during childbirth, postpartum or post-abortion? [yes/no/can't remember]

III. Personal information

Remember! These data are collected for categorization purposes only. Your information is confidential and you will not be identified in any future publications on this study.

19. Age range
20. Gender: [male, female, other]

For peer review only - http://bmjopen.bmj.com/site/about/guidelines.xhtml

21. Qualification: [nurse, midwife, physician/medical doctor, resident/physician in training, community health worker, social worker, other: please specify].
22. Years of work experience in current setting: [years|months]
23. Total years of work experience (since completing your training): [number entry/2-digit max]
24. Place of work: [list of countries]
25. Location (of current or main place of work): [urban/rural]
26. Name of facility & address [Text box]
27. Facility type (of current or main place of work): [Clinic, Health centre, Maternity hospital, Regional/Provincial hospital, District hospital, Other hospital, other]
28. Facility management (of current or main place of work): [private, public, social insurance, NGO, other]
29. Did you participate in this year's World Sepsis Congress Spotlight on Maternal and Neonatal Sepsis (held on 12 September 2017)? [yes/no]

IV. Future contacts

30. The global maternal study and awareness campaign would like to contact you at a future date for a follow-up on this survey. If you agree to being contacted again, please provide us with your email address.

Your contact details will be securely stored by the WHO staff person working on the study for one year. You can contact us to modify or suppress your information at any time. To do so, please contact Ms Vanessa Brizuela (brizuelav@who.int).

- ☐ I agree.
- ☐ I do not agree

Please provide us with your email address [text box]

Thank you very much for participating in this survey! Your responses are extremely valuable to us in our efforts to improve the health of women and newborns.

For peer review only - http://bmjopen.bmj.com/site/about/guidelines.xhtml

GLOSS Post-Campaign Survey

This online survey is part of the activities set forth for a global study on maternal morbidity and mortality. This study is being conducted in approximately 50 countries across the globe, including your own, and it is coordinated by the World Health Organization and the healthcare facility where you work. As part of this study, we want to learn more from healthcare providers about how you identify and manage women with certain conditions during pregnancy, childbirth or postpartum or post- abortion.

A few months ago, you received a similar survey from us. At that point we asked that you provide us with an email address so that we could send you a follow-up questionnaire. We will ask again that you respond to a number of questions on your knowledge, attitudes, and practices around maternal and neonatal health. This is not a test; this is an opportunity to let us know your thoughts and experience on the topic as a healthcare provider in one of the hospitals participating in the study.

This survey is voluntary, and your answers will be kept confidential, and you can choose whether to leave some questions unanswered. General information about you, your position, and geographical location will be collected to help us categorize respondents only but will not be used to identify you in particular. You are free to provide this information at the end of the survey. Results of these surveys will be published in a peer-reviewed journal without attributing responses to any specific person or institution.

The completion of this survey implies your consent to participate.

If you have any question about the survey, please contact Ms Vanessa Brizuela (brizuelav@who.int)

For peer review only - http://bmjopen.bmj.com/site/about/guidelines.xhtml

I. General knowledge and attitudes

The following questions will ask that you respond according to your current role, competences, and skills depending on your training and background. According to these, you may be the person triaging, prescribing, diagnosing, treating. Bear this in mind when responding.

1. What are the main conditions causing death and disability among women during pregnancy and/or childbirth in your hospital? Check all that apply [abortion-related complications, chronic/pre-existing disease, embolism, haemorrhage, infection/sepsis, pre-eclampsia/eclampsia, other: please specify]
2. Case vignettes:

   Case A: A 25-year-old 32- week pregnant woman comes to your facility brought by a family member saying she is feeling unwell. Her companion reports that she seems a bit disoriented and feverish. Without any further diagnostic testing or triaging:

   a. What would you first think could be causing her to feel this way? Choose from the following list [abortion-related complications, embolism, haemorrhage, infection/sepsis, pre-eclampsia/eclampsia, other]
   b. What would be the first two things this woman should receive? [antibiotics, blood transfusion, body fluid culture, fetal monitoring, fluids, haematology/biochemistry laboratory, other antimicrobials (i.e. antimalarials), other laboratory test, other medication, oxygen, physical exam, urine output measurement, other]
3. Case B: A recently pregnant woman comes to your facility complaining that she has abdominal pain and shortness of breath. Without any further diagnostic testing or triaging:
   a. What would you first think could be causing her to feel this way? Choose from the following list [abortion-related complications, embolism, haemorrhage, infection/sepsis, pre-eclampsia/eclampsia, other]
   b. What would be the first two things this woman should receive? [antibiotics, blood transfusion, body fluid culture, fetal monitoring, fluids, haematology/biochemistry laboratory, other antimicrobials (i.e. antimalarials, ART), other laboratory test, other medication, oxygen, physical exam, urine output measurement, other]
4. How confident do you feel that you are capable of making the right decision in a case like the one above? [very confident, somewhat confident, indifferent/neutral, not too confident, not confident at all] n
5. How would you qualify the availability of resources in the facility where you work to help you make the right decisions? [always available, somewhat available, indifferent/neutral, not always available, not available at all].
6. How supported do you feel by the facility in which you work to make the right decision in a case like the one above? [very supported, somewhat supported, neutral, not very supported, unsupported].
7. How well does this statement describe your facility: "The facility where I work doesn't let me handle cases like the one described above." [very well, somewhat well, indifferent/neutral, somewhat incorrectly, completely incorrectly]

For peer review only - http://bmjopen.bmj.com/site/about/guidelines.xhtml

8. Of the following, which do you think are the greatest barriers in making a right, and timely decision in your facility? Check one option (the one most accurate to your situation). [not sure I know the correct signs, I'm afraid of making a mistake, my supervisor doesn't let me make them, we don't have a way to triage/treat/manage cases like these in my hospital, I've never seen cases like these, There are no barriers to making a right and timely decision in my facility, other]
9. Does the hospital you work in have protocols in place for dealing with cases like the one described above? [yes/no/don't know]

DO NOT ALLOW GOING BACK AFTER THIS QUESTION

10. Have you ever heard of the term maternal sepsis? [yes/no]
11. If yes, how did you hear about this? Check all that apply [pre-service training, in-service training, colleagues, public health campaign, media (TV/radio/newspaper), other: please specify].
12. What two criteria best describe maternal sepsis? Check two options [abnormal white cell count, altered mental status, elevated heart rate, excessively rapid respiration, fever, infection, low blood pressure, organ dysfunction, other]
13. What supplies/commodities are essential to effectively identify sepsis among women during pregnancy, childbirth, postpartum or post-abortion? Check all that apply [blood culture, blood pressure apparatus, diagnostic imaging, laboratory (haematology/biochemistry), rapid test for infectious disease, serum lactate measurement, thermometer, urine output measurement, other]
14. What supplies/commodities are essential to effectively manage sepsis in women during pregnancy, childbirth, postpartum or post-abortion? Check all that apply [antibiotics, blood transfusions, fluids, intensive care/high-dependency unit, other antimicrobials (e.g. antimalarials, ART), oxygen, urine output measurement, other]

II. Context

15. How many women are affected by maternal sepsis in your facility every year? Give your best estimate (a whole number), given your experience in the facility [Text box]
16. How many neonates are affected by neonatal sepsis in the first week of life in your facility? Give your best estimate (a whole number), given your experience in the facility. [Text box]
17. How many deliveries occur every year, on average, in your facility? Give your best estimate (a whole number). [Text box]
18. Have you ever received specific training in how to manage women who present with signs of infection while pregnant, during childbirth, postpartum or post-abortion? [yes/no/can't remember]

III. Campaign

19. Did you notice any materials in your facility that related to maternal and neonatal sepsis? [yes/no] [if no go to question 27]

For peer review only - http://bmjopen.bmj.com/site/about/guidelines.xhtml

20. If yes, where did you see them? Check all that apply [labour ward, antenatal ward, postnatal ward, emergency room/department, ICU/high dependency room, patient waiting area, other: please specify]
21. If yes, what materials did you see? Check all that apply [informational posters about the study, graphic materials with information about maternal sepsis, campaign website, social media, press releases, none of the above].
    a. Did you ever visit the website? [yes/no/N/A]
    b. Did you ever tweet/re-tweet a message about the campaign? [yes/no/I don't use Twitter]
22. What were the main messages of these materials? Check all that apply ["stop sepsis", "think sepsis", "right care, right now", "sepsis is life-threatening but when caught early and treated promptly it can be stopped", "just ask, could it be sepsis?", "a world free of sepsis", "together, we can stop maternal sepsis", "stop sepsis save lives", "surviving sepsis", none of the above]
23. Did you find the materials and messaging helpful? [yes/no]
    a. If yes, why? Check all that apply [they provided information about maternal sepsis, they invited me to act, they mentioned things I did not know, they helped explain the study to me, they were visually appealing, they were in a language I could understand, other]
    b. If no, why not? Check all that apply [they didn't provide any new information, I didn't know what to do, the message was confusing, they were difficult to understand, they were in a language I don't speak/read, they had too much text, they had too many images, the colours didn't work in our hospital, they were unappealing, other]
24. Did the materials help you identify cases of women with infection or sepsis? [yes/no]
25. Did the information provided in the materials motivate you to do something differently than before? [yes/no]
    a. If yes, in what way? Check all that apply [to suspect maternal sepsis among women presenting with specific signs, to act fast when I think a woman could have sepsis, to implement health measures to prevent sepsis, to talk about sepsis with others, to learn more about sepsis, other: please specify]
    b. If no, why not? Please explain [text box]
26. In your opinion, did the materials help to increase awareness about maternal sepsis in your facility? [yes/no]
27. Did you know that your facility participated in a global maternal sepsis study? [yes/no]
28. Did you know that your facility participated in an awareness campaign for the global maternal sepsis study? [yes/no]

IV. Personal information

Remember! These data are collected for categorization purposes only. Your information is confidential and you will not be identified in any future publications on this study.

29. Age range [18-25; 26-30; 31-40; 41-50; 51-60; 60+]
30. Gender: [male, female, other]
31. Qualification: [community health worker, midwife, nurse, physician/medical doctor, resident/physician in training, social worker, student, physical therapist, auxiliary nurse, other: please specify].

For peer review only - http://bmjopen.bmj.com/site/about/guidelines.xhtml

32. Years of work experience in current setting: [years | months]
33. Total years of work experience (since completing your training): [2-digit max]
34. Place of work: [list of countries]
35. Location (of current or main place of work): [urban/rural]
36. Name of facility and address [text entry]
37. Facility type (of current or main place of work): [Clinic, Health centre, Maternity hospital, Regional/Provincial hospital, District hospital, Other hospital, other]
38. Facility management (of current or main place of work): [private, public, social insurance, NGO, other]
39. Did you participate in the World Sepsis Congress Spotlight on Maternal and Neonatal Sepsis (held on 12 September 2017)? [yes/no]

V. Future contacts

We might be interested in contacting you in the future with more information about this study or to get further information about your responses to this survey. Please provide us with your email address below if you agree with this.

- ☐ I agree.
- ☐ I do not agree

Your contact details will be securely stored by the WHO staff person working on the Global Maternal and Neonatal Initiative for one year. You can contact us to modify or suppress your information at any time. To do so, please contact Ms Vanessa Brizuela (brizuelav@who.int)

40. Please type your email address below [text entry]

Thank you very much for participating in this survey! Your responses are extremely valuable to us in our efforts to improve the health of women and babies.

Please visit http://srhr.org/sepsis for more information about the study, the campaign, and maternal sepsis.

For peer review only

For peer review only - http://bmjopen.bmj.com/site/about/guidelines.xhtml

Appendix 2. Respondent perception of enabling environments for maternal sepsis identification and management at pre- and post-campaign and changes after campaign implementation, limiting to most favourable responses (N=2,188)

	Pre-campaign n/N (%)	Post-campaign n/N (%)	Pre-post cOR‡ [95% CI]¶	Percentage change %
Perception of enabling environment for maternal sepsis identification and management				
Very confident of making right decisions	390/1,155 (33.8)	395/1,033 (38.2)	1.21* [1.05-1.41]	13.2
Resources ***always*** available to make right decisions	443/1,155 (38.4)	386/1,033 (37.4)	0.96 [0.68-1.35]	-2.6
Very supported by facility in making right decisions	503/1,155 (43.6)	438/1,033 (42.4)	0.95 [0.67-1.37]	-2.6

cOR: crude odds ratio; CI: confidence interval; OR calculated clustering at the country level
Percentage change: [(% in post - % in pre)/% in pre]x100
‡Refers to odds ratio between pre- and post-campaign; ¶reference group: pre-campaign; **p<0.05*

Responses were dichotomized as follows: a 1 was assigned to the most favourable response (i.e., they felt very confident *about being capable of making the right decision) and a 0 to a combination of all the remaining options (i.e.,* somewhat confident, neutral, not very confident *or* not confident at all*)*

For peer review only - http://bmjopen.bmj.com/site/about/guidelines.xhtml

Appendix 3. Changes in respondent knowledge and perception of enabling environments after campaign implementation among different sub-groups: [1] sample restricted to ≥1 response per facility at pre- and post-campaign; [2] sample restricted to ≥2 responses per facility at pre- and post-campaign; [3] sample restricted to countries with >30 responses at pre- or post-campaign

	**Full sample (N=2,188)**		Restricted sample ≥1 response per facility (N=1,872)		Restricted sample ≥2 responses per facility (N=1,645)		Restricted sample >30 responses per country (N=1,680)	
	**Pre-post OR‡ [95% CI]¶**	**% change**	Pre-post OR‡ [95% CI] ¶	% change	Pre-post OR‡ [95% CI] ¶	% change	Pre-post OR‡ [95% CI] ¶	% change
Knowledge on maternal sepsis								
Have not heard of maternal sepsis	**0.35* [0.18-0.68]**	**-63.4**	0.36* [0.17-0.77]	-61.82	0.45* [0.22-0.93]	-53.58	0.34* [0.15-0.79]	-63.29
Correctly identified the two criteria to define maternal sepsis	**0.67 [0.43-1.03]**	**-29.4**	0.63 [0.39-1.01]	-33.19	0.57* [0.34-0.93]	-38.76	0.57* [0.33-0.98]	-39.46
Correctly identified management of sepsis when maternal sepsis was suspected	**1.76 [0.73-4.21]**	**30.8**	1.58 [0.58-4.30]	24.05	1.53 [0.51-4.62]	21.70	1.70 [0.51-5.72]	24.89
Perception of enabling environment for maternal sepsis identification and management								
Confident of making right decisions	**1.44* [1.01-2.06]**	**7.3**	1.36 [0.97-1.90]	6.39	1.33 [0.94-1.88]	6.34	1.45 [0.95-2.22]	7.99
Resources available to make right decisions	**1.01 [0.68-1.49]**	**0.1**	1.12 [0.76-1.66]	2.32	1.12 [0.72-1.74]	2.23	0.92 [0.57-1.50]	-1.62
Supported by facility in making right decisions	**1.11 [0.80-1.54]**	**2.0**	1.21 [0.84-1.73]	3.44	1.19 [0.81-1.76]	3.45	0.99 [0.69-1.44]	-0.11

OR: odds ratio; CI: confidence interval; percentage change= [(% in post - % in pre)/% in pre]x100

‡Refers to odds ratio between pre- and post-campaign, OR calculated clustering at the country level; ¶reference group pre-campaign; **p<0.05*

For peer review only - http://bmjopen.bmj.com/site/about/guidelines.xhtml

Appendix 4a. Changes in respondent knowledge after campaign implementation according to respondent and facility characteristics (N= 2,188)

	**Had not heard about maternal sepsis**						**Correctly identified the two criteria to define maternal sepsis**						**Correctly identified management of sepsis when maternal sepsis was suspected**					
Respondent characteristics	*Pre-campaign (N=1,144)*		*Post-campaign (N=1,021)*		*OR [95% CI]¶*	*% change*	*Pre-campaign (N=673)*		*Post-campaign (N= 647)*		*OR [95% CI]¶*	*% change*	*Pre-campaign (N=251)*		*Post-campaign (N=239)*		*OR [95% CI]¶*	*% change*
	n	%	N	%		%	n	%	n	%		%	n	%	n	%		%
Overall	*95*	*8*	*31*	*3*	*0.35* [0.23-0.52]*	*-63.4*	*109*	*16*	*74*	*11*	*0.67 [0.43-1.03]*	*-29.4*	*114*	*45*	*142*	*59*	*1.76 [0.73-4.21]*	*30.8*
Qualification	*1,141*		*1,013*				*670*		*645*				*250*		*235*			
Nurse/midwife	74	17	16	4	0.18* [0.10-0.33]	-79.2	14	6	8	3	0.48 [0.20-1.14]	-50.3	24	28	41	46	2.13 [0.53-8.47]	61.3
Physician	16	3	10	2	0.77 [0.26-2.31]	-22.6	72	21	55	19	0.87 [0.55-1.38]	-10.3	55	50	75	67	2.06* [1.09-3.91]	35.1
Resident	4	3	4	4	1.36 [0.13-14.31]	34.3	23	27	11	15	0.48 [0.11-2.11]	-44.4	34	63	25	76	1.84 [0.49-6.90]	20.3
Years of experience	*1,098*		*963*				*642*		*607*				*242*		*229*			
<10	20	4	14	3	0.79 [0.28-2.24]	-19.9	58	18	32	11	0.54 [0.28-1.02]	-41.3	67	48	72	58	1.50 [0.40-5.58]	21.2
10-20	36	10	8	3	0.22* [0.10-0.50]	-75.9	28	15	24	12	0.76 [0.44-1.33]	-21.0	31	47	44	66	2.16* [1.04-4.49]	39.8
>20	26	12	4	2	0.17* [0.08-0.38]	-81.0	22	16	16	16	0.95 [0.53-1.69]	-4.4	13	37	21	57	2.22 [0.72-6.87]	52.8
Region	*1,144*		*1,021*				*673*		*647*				*251*		*239*			
Africa	14	6	4	2	0.27* [0.15-0.49]	-71.6	18	15	19	15	1.04 [0.46-2.31]	3.1	30	53	46	69	1.97 [0.95-4.08]	30.5
Asia	11	7	9	5	0.83 [0.25-2.76]	-15.7	7	5	7	6	1.06 [0.42-2.65]	5.7	4	14	8	23	1.85 [0.37-9.21]	65.8
Eastern Mediterranean	58	44	13	8	0.16* [0.09-0.31]	-81.7	2	3	8	9	3.19 [1.04-9.78]	200.0	2	12	10	45	6.25 [0.88-44.14]	286.5
Europe‡	7	5	2	2	0.39 [0.07-2.23]	-59.7	27	38	12	27	0.63* [0.41-0.96]	-27.3	5	12	4	27	1.60 [0.77-3.33]	126.8
Latin America	5	1	3	1	0.72 [0.07-7.77]	-27.9	55	20	28	11	0.49 [0.22-1.08]	-45.8	73	12	74	74	1.87 [1.17-20.42]	529.3

For peer review only

For peer review only - http://bmjopen.bmj.com/site/about/guidelines.xhtml

Country implemented an expanded version of campaign	*1,144*		*1,021*				*673*		647				251		239			
Yes	74	11	13	3	0.23* [0.16-0.32]	-75.2	75	19	37	12	0.58 [0.32-1.05]	-37	92	53	105	76	2.77 [0.88-8.69]	42
No	21	5	18	3	0.71 [0.35-1.45]	-27.6	34	12	37	11	0.89 [0.52-1.53]	-10	22	28	37	38	1.50 [0.51-4.40]	32
Respondent worked in a level III facility	*1,144*		*1,021*				*673*		*647*				*251*		*239*			
Yes	23	3	16	3	0.91 [0.38-2.18]	-8.5	87	18	58	15	0.80 [0.45-1.41]	-17.1	104	51	108	65	1.77 [0.51-6.10]	27.0
No	72	20	15	4	0.15* [0.08-0.28]	-82.3	22	11	16	6	0.51 [0.25-1.03]	-46.3	10	21	34	47	3.31* [1.54-7.12]	123.6

‡Includes countries in Central Asia (Kazakhstan, Kyrgyzstan, and Tajikistan)

OR: odds ratio, OR calculated clustering at the country level; CI: confidence interval; Percentage change= [(% in post - % in pre)/% in pre]x100

¶Reference group: pre-campaign

Where n represents the frequency and N the denominator (i.e. n= the number of respondents with a specific characteristic who answered correctly, N= the total number of persons who answered that question)

For peer review only

For peer review only - http://bmjopen.bmj.com/site/about/guidelines.xhtml

Appendix 4b. **Changes in respondent perception of enabling environments after campaign implementation according to respondent and facility characteristics**

Respondent characteristic	*Confident of making right decisions*						*Resources available to make right decisions*						*Supported by facility in making right decisions*					
	Pre-campaign (N=1,155)		*Post-campaign (N=1,033)*		*OR [95% CI]¶*	*% change*	*Pre-campaign (N=1,155)*		*Post-campaign (N=1,033)*		*OR [95% CI]¶*	*% change*	*Pre-campaign (N=1,155)*		*Post-campaign (N=1,033)*		*OR [95% CI]¶*	*% change*
	n	*%*	*n*	*%*		*%*	*n*	*%*	*n*	*%*		*%*	*N*	*%*	*n*	*%*		*%*
Overall	*897*	*78*	*861*	*83*	*1.44* [1.01-2.06]*	*7.3*	*909*	*79*	*814*	*79*	*1.01 [0.68-1.49]*	*0.1*	*921*	*80*	*840*	*81*	*1.11 [0.80-1.54]*	*2.0*
Qualification	*1,151*		*1,025*				*1,151*		*1,025*				*1,151*		*1,025*			
Nurse/midwife	355	81	371	81	1.05 [0.56-1.96]	0.8	356	81	336	74	0.66 [0.34-1.28]	-8.9	367	83	347	76	0.63* [0.41-0.98]	-8.8
Physician	440	78	395	87	1.78* [1.23-2.57]	10.4	445	79	387	85	1.46* [1.17-1.82]	7.0	448	80	393	86	1.57* [1.12-2.22]	7.9
Resident	100	67	87	77	1.67 [0.97-2.88]	15.5	105	70	86	76	1.37 [0.77-2.42]	8.7	104	69	95	84	2.33* [1.24-4.38]	21.3
Years of experience	*1,107*		*970*				*1,107*		*970*				*1,107*		*970*			
<10	409	76	389	82	1.44 [0.99-2.10]	8.1	416	77	379	80	1.17 [0.80-1.73]	3.6	424	78	390	82	1.25 [0.86-1.83]	4.5
10-20	284	81	264	83	1.08 [0.73-1.59]	1.4	277	79	239	75	0.77 [0.52-1.13]	-5.9	280	80	248	78	0.85 [0.59-1.23]	-3.4
>20	179	82	160	92	2.43* [1.13-5.21]	11.5	187	86	153	88	1.17 [0.72-1.91]	2.0	188	87	158	91	1.52 [0.93-2.49]	4.8
Region	*1,155*		*1,033*				*1,155*		*1,033*				*1,155*		*1,033*			
Africa	189	84	198	88	1.31 [0.76-2.26]	3.8	158	71	157	69	0.95 [0.73-1.24]	-1.5	176	79	173	77	0.89 [0.63-1.26]	-2.6
Asia	128	74	145	85	2.04* [1.17-3.55]	15.3	134	77	150	88	2.18* [1.72-2.76]	13.9	137	79	151	89	2.09* [1.59-2.74]	12.2
Eastern Mediterranean	131	77	115	70	0.70* [0.64-0.77]	-9.0	109	64	84	51	0.59* [0.49-0.71]	-20.1	108	63	97	59	0.83 [0.61-1.14]	-6.9
Europe‡	97	71	77	79	1.59 [0.89-2.82]	12.1	112	82	83	86	1.32 [0.80-2.20]	4.7	112	82	88	91	2.18* [1.54-3.09]	11.0
Latin America	352	78	326	87	1.85* [1.14-3.01]	11.1	396	88	340	91	1.32 [0.68-2.57]	3.0	388	86	331	88	1.20 [0.59-2.44]	2.4

For peer review only

For peer review only - http://bmjopen.bmj.com/site/about/guidelines.xhtml

Country implemented an expanded version of campaign	*1,155*		*1,033*				*1,155*		*1,033*				*1,155*		*1,033*			
Yes	531	75	398	80	1.28 [0.76-2.15]	5.7	554	79	375	75	0.82 [0.45-1.48]	-4.6	567	80	391	78	0.87 [0.53-1.43]	-2.8
No	366	81	463	87	1.52* [1.00-2.29]	6.8	355	79	439	82	1.25 [0.83-1.89]	4.4	354	79	449	84	1.45* [1.03-2.05]	7.1
Respondent worked in a level III facility	*1,155*		*1,033*				*1,155*		*1,033*				*1,155*		*1,033*			
Yes	608	77	508	83	1.51* [1.10-2.05]	8.4	646	82	516	85	1.24 [0.85-1.79]	3.6	640	81	518	85	1.33 [0.95-1.87]	5.0
No	289	79	353	83	1.31 [0.77-2.22]	5.1	263	72	298	70	0.92 [0.62-1.36]	-2.5	281	77	322	76	0.94 [0.71-1.25]	-1.4

‡Includes countries in Central Asia (Kazakhstan, Kyrgyzstan, and Tajikistan)

OR: odds ratio, OR calculated clustering at the country level; CI: confidence interval; Percentage change= [(% in post - % in pre)/% in pre]x100

¶Reference group: pre-campaign

Where n represents the frequency and N the denominator (i.e. n= the number of respondents with a specific characteristic who answered correctly, N= the total number of persons who answered that question)

For peer review only

For peer review only - http://bmjopen.bmj.com/site/about/guidelines.xhtml

STROBE Statement - 'STOP SEPSIS!': An evaluation of the WHO global maternal sepsis study awareness campaign (Brizuela et al)

	Item No	Recommendation	Page in manuscript
**Title and abstract**	1	(*a*) Indicate the study's design with a commonly used term in the title or the abstract	1
		(*b*) Provide in the abstract an informative and balanced summary of what was done and what was found	3
**Introduction**			
Background/rationale	2	Explain the scientific background and rationale for the investigation being reported	5-6
Objectives	3	State specific objectives, including any prespecified hypotheses	5-6
**Methods**			
Study design	4	Present key elements of study design early in the paper	6
Setting	5	Describe the setting, locations, and relevant dates, including periods of recruitment, exposure, follow-up, and data collection	6-8
Participants	6	(*a*) *Cohort study*—Give the eligibility criteria, and the sources and methods of selection of participants. Describe methods of follow-up<br>*Case-control study*—Give the eligibility criteria, and the sources and methods of case ascertainment and control selection. Give the rationale for the choice of cases and controls<br>*Cross-sectional study*—Give the eligibility criteria, and the sources and methods of selection of participants	9
Variables	7	Clearly define all outcomes, exposures, predictors, potential confounders, and effect modifiers. Give diagnostic criteria, if applicable	8-11
Data sources/ measurement	8*	For each variable of interest, give sources of data and details of methods of assessment (measurement). Describe comparability of assessment methods if there is more than one group	8-10
Bias	9	Describe any efforts to address potential sources of bias	9
Study size	10	Explain how the study size was arrived at	9
Quantitative variables	11	Explain how quantitative variables were handled in the analyses. If applicable, describe which groupings were chosen and why	9-11
Statistical methods	12	(*a*) Describe all statistical methods, including those used to control for confounding	8-10
		(*b*) Describe any methods used to examine	8-10

For peer review only - http://bmjopen.bmj.com/site/about/guidelines.xhtml

		subgroups and interactions	
		(*c*) Explain how missing data were addressed	Tables 1, 2, 3, and 4
		(*d*) *Cohort study*—If applicable, explain how loss to follow-up was addressed<br>*Case-control study*—If applicable, explain how matching of cases and controls was addressed<br>*Cross-sectional study*—If applicable, describe analytical methods taking account of sampling strategy	Not applicable
		(*e*) Describe any sensitivity analyses	10
**Results**			
Participants	13*	(a) Report numbers of individuals at each stage of study—eg numbers potentially eligible, examined for eligibility, confirmed eligible, included in the study, completing follow-up, and analysed	Tables 1, 2, 3 and 4
		(b) Give reasons for non-participation at each stage	Not applicable
		(c) Consider use of a flow diagram	Not applicable
Descriptive data	14*	(a) Give characteristics of study participants (eg demographic, clinical, social) and information on exposures and potential confounders	9; table 1, figure1
		(b) Indicate number of participants with missing data for each variable of interest	Tables 1, 2, 3, and 4
		(c) *Cohort study*—Summarise follow-up time (eg, average and total amount)	Not applicable
Outcome data	15*	*Changes in awareness*—Report numbers of outcome events or summary measures	14-15, tables 2, 3, and 4
		Campaign recognition and exposure—Report numbers of outcome events or summary measures	13-14; figures 2 and 3
Main results	16	(*a*) Give unadjusted estimates and, if applicable, confounder-adjusted estimates and their precision (eg, 95% confidence interval). Make clear which confounders were adjusted for and why they were included	16; tables 3 and 4
Other analyses	17	Report other analyses done—eg analyses of subgroups and interactions, and sensitivity analyses	10, 15-16; appendices 2 & 3
**Discussion**			
Key results	18	Summarise key results with reference to study objectives	19
Limitations	19	Discuss limitations of the study, taking into account sources of potential bias or imprecision. Discuss both direction and magnitude of any potential bias	21-22
Interpretation	20	Give a cautious overall interpretation of results considering objectives, limitations, multiplicity of analyses, results from similar studies, and other relevant evidence	19-22

For peer review only

For peer review only - http://bmjopen.bmj.com/site/about/guidelines.xhtml

Generalisability	21	Discuss the generalisability (external validity) of the study results	22
**Other information**			
Funding	22	Give the source of funding and the role of the funders for the present study and, if applicable, for the original study on which the present article is based	11 and 24

For peer review only

BMJ Open

Early evaluation of the 'STOP SEPSIS!' WHO Global Maternal Sepsis Awareness Campaign implemented for healthcare providers in 46 low- middle- and high-income countries

Journal:	*BMJ Open*
Manuscript ID	bmjopen-2019-036338.R1
Article Type:	Original research
Date Submitted by the Author:	01-Apr-2020
Complete List of Authors:	Brizuela, Vanessa; WHO, UNDP/UNFPA/UNICEF/WHO/World Bank Special Programme of Research, Development and Research Training in Human Reproduction (HRP), Department of Reproductive Health and Research<br>Bonet, Mercedes; World Health Organization, UNDP/UNFPA/UNICEF/WHO/World Bank Special Programme of Research, Development and Research Training in Human Reproduction (HRP), Department of Reproductive Health and Research<br>Trigo Romero, Carla Lionela; University of Sao Paulo Faculty of Medicine of Ribeirao Preto, Department of Social Medicine<br>Abalos, E; Centro Rosarino de Estudios Perinatales<br>Baguiya, Adama; Research Institute of Health Sciences<br>Fawole, Adeniran O.; University of Ibadan College of Medicine<br>Knight, Marian; University of Oxford, National Perinatal Epidemiology Unit<br>Lumbiganon, Pisake; Khon Kaen University, Ob & Gyn<br>Minkauskienė, Meilė; Lithuanian University of Health Sciences, Department of Obstetrics and Gynaecology<br>Nabhan, Ashraf; Ain Shams University, Department of Obstetrics & Gynaecology<br>Osman, Nafissa; Hospital Central de Maputo<br>Qureshi , Zahida ; University of Nairobi Department of Obstetrics and Gynecology,<br>Souza, Joao Paulo; University of Sao Paulo Faculty of Medicine of Ribeirao Preto, Department of Social Medicine
**Primary Subject Heading**:	Global health
Secondary Subject Heading:	Global health, Obstetrics and gynaecology, Public health
Keywords:	Maternal medicine < OBSTETRICS, PERINATOLOGY, PUBLIC HEALTH

SCHOLARONE™ Manuscripts

BMJ

I, the Submitting Author has the right to grant and does grant on behalf of all authors of the Work (as defined in the below author licence), an exclusive licence and/or a non-exclusive licence for contributions from authors who are: i) UK Crown employees; ii) where BMJ has agreed a CC-BY licence shall apply, and/or iii) in accordance with the terms applicable for US Federal Government officers or employees acting as part of their official duties; on a worldwide, perpetual, irrevocable, royalty-free basis to BMJ Publishing Group Ltd ("BMJ") its licensees and where the relevant Journal is co-owned by BMJ to the co-owners of the Journal, to publish the Work in this journal and any other BMJ products and to exploit all rights, as set out in our licence.

The Submitting Author accepts and understands that any supply made under these terms is made by BMJ to the Submitting Author unless you are acting as an employee on behalf of your employer or a postgraduate student of an affiliated institution which is paying any applicable article publishing charge ("APC") for Open Access articles. Where the Submitting Author wishes to make the Work available on an Open Access basis (and intends to pay the relevant APC), the terms of reuse of such Open Access shall be governed by a Creative Commons licence – details of these licences and which Creative Commons licence will apply to this Work are set out in our licence referred to above.

Other than as permitted in any relevant BMJ Author's Self Archiving Policies, I confirm this Work has not been accepted for publication elsewhere, is not being considered for publication elsewhere and does not duplicate material already published. I confirm all authors consent to publication of this Work and authorise the granting of this licence.

For peer review only

Early evaluation of the 'STOP SEPSIS!' WHO Global Maternal Sepsis Awareness Campaign implemented for healthcare providers in 46 low- middle- and high-income countries

Authors and affiliations:

Vanessa Brizuela, UNDP/UNFPA/UNICEF/WHO/World Bank Special Programme of Research, Development and Research Training in Human Reproduction (HRP), Department of Reproductive Health and Research, World Health Organization, Avenue Appia 20, 1211 Geneva, Switzerland. Email: brizuelav@who.int. Telephone: +41 22 791 37 40.

Mercedes Bonet, UNDP/UNFPA/UNICEF/WHO/World Bank Special Programme of Research, Development and Research Training in Human Reproduction (HRP), Department of Reproductive Health and Research, World Health Organization, Geneva, Switzerland.

Carla Lionela Trigo Romero, Department of Social Medicine. Ribeirão Preto Medical School, Ribeirão Preto, Brazil.

Edgardo Abalos, Centro Rosarino de Estudios Perinatales (CREP), Rosario, Argentina.

Adama Baguiya, Research Institute for Health Sciences, Ouagadougou, Burkina Faso.

Adeniran Olubukola Fawole†, College of Medicine, University of Ibadan, Ibadan, Nigeria.

Marian Knight, National Perinatal Epidemiology Unit, Nuffield Department of Population Health, University of Oxford, United Kingdom.

Pisake Lumbiganon, Department of Obstetrics and Gynaecology, Faculty of Medicine, Khon Kaen University, Khon Kaen, Thailand.

For peer review only - http://bmjopen.bmj.com/site/about/guidelines.xhtml

Meilė Minkauskienė, Department of Obstetrics and Gynaecology, Lithuanian University of Health Sciences, Kaunas, Lithuania.

Ashraf Nabhan, Department of Obstetrics & Gynaecology, Ain Shams University, Cairo, Egypt.

Nafissa Bique Osman, Faculty of Medicine, Eduardo Mondlane University, Maputo, Mozambique.

Zahida Qureshi, Department of Obstetrics and Gynaecology, University of Nairobi, Nairobi, Kenya.

João Paulo Souza, Department of Social Medicine. Ribeirão Preto Medical School, Ribeirão Preto, Brazil.

On behalf of the WHO GLOSS Research Group

†Deceased January 2019

Correspondence to: Vanessa Brizuela. Email: brizuelav@who.int.

Key words: maternal sepsis, maternal infections, awareness campaign, evaluation, multi-country study

Word count: 3,722

For peer review only

Abstract (268 words)

Objective: To evaluate changes in awareness of maternal sepsis among healthcare providers resulting from the WHO global maternal sepsis study (GLOSS) awareness campaign.

Design: Independent sample pre-post intervention through online and paper-based surveys available for over 30 days before campaign rollout (pre) and after study data collection (post). Descriptive statistics were used for campaign recognition and exposure, and odds ratio and percentage change were calculated for differences in awareness, adjusting for confounders using multivariate logistic regression.

Setting and participants: Healthcare providers from 398 participating facilities in 37 low-, middle-, and high-income countries.

Intervention: An awareness campaign to accompany GLOSS launched three weeks prior to data collection and lasting the entire study period (28 November 2017 to 15 January 2018) and beyond.

Main outcome measures: Campaign recognition and exposure, and changes in awareness.

Results: A total of 2,188 surveys were analysed: 1,155 at baseline and 1,033 at post-intervention. Most survey respondents found the campaign materials helpful (94%), that they helped increase awareness (90%), and that they helped motivate to act differently (88%). There were significant changes with regards to: not having heard of maternal sepsis (-63.4% change, pre-post OR 0.35, 95% CI 0.18-0.68) and perception of confidence in making the right decisions with regards to maternal sepsis identification and management (7.3% change, pre-post OR 1.44, 95% CI 1.01-2.06).

Conclusions: Awareness raising campaigns can contribute to an increase in having heard of maternal sepsis and an increase in provider perception of confidence in making correct decisions. Offering the

For peer review only - http://bmjopen.bmj.com/site/about/guidelines.xhtml

information to make accurate and timely decisions while promoting environments that enable self-confidence and support could improve maternal sepsis identification and management.

Strengths and limitations of this study

- This study presents the results of an evaluation of a global awareness campaign which accompanied a research study implemented in 46 countries.
- This evaluation was a cost-effective, feasible way in which to assess campaign effectiveness among a varied and global population of healthcare providers.
- Our pre-post design using anonymous surveys with no control group does not allow to discern the impact of the campaign alone or matching pre- and post- campaign responses.
- Campaign fidelity was only assessed through healthcare provider self-report at post-campaign surveys.
- This evaluation was restricted to the duration of the study follow-up period limiting understanding of long-term impact.

For peer review only

For peer review only - http://bmjopen.bmj.com/site/about/guidelines.xhtml

INTRODUCTION

The global health community has recently drawn attention to the importance of sepsis and its toll on global mortality and morbidity.[1–3] In 2017, the World Health Assembly approved a resolution on sepsis to improve the prevention, diagnosis, and management of sepsis.[4] With updates in 2017 and 2018, the Surviving Sepsis Campaign has been developing guidelines for management and recommended bundles of care for sepsis among adult populations, not specific to pregnant or recently pregnant women, since 2002.[5–7]

Infections and sepsis remain major causes of death and disability among women during pregnancy, childbirth, postpartum, and post-abortion.[8,9] To respond to this, the Global Maternal Sepsis Initiative was launched in 2016.[4,10] Building on the 2016 SEPSIS-3 definition,[11] the World Health Organization (WHO) led the development of a definition for maternal sepsis as "a life-threatening condition defined as organ dysfunction resulting from infection during pregnancy, childbirth, postpartum, and post-abortion."[12] And in 2017, WHO led the Global Maternal Sepsis Study and Awareness Campaign (GLOSS) to assess the burden of maternal infections and sepsis, to validate identification criteria for possible severe maternal infection and maternal sepsis, and to raise awareness on maternal sepsis among healthcare providers working in study participating facilities.[12]

These initiatives and calls to action all share a recommendation to increase awareness of sepsis among healthcare providers, policy-makers, and the public, in pursuit of reducing the global burden of sepsis.

Awareness raising has mostly been attempted through campaigns. These have been implemented to increase knowledge, improve attitudes, or change behaviours around different health issues.[13–15] Specific to sepsis, the UK Sepsis Trust heads a campaign on sepsis since 2012 and the Global Sepsis

For peer review only

59 Alliance leads efforts aimed at raising sepsis awareness since 2010.[16,17] However, neither of these two large campaigns have been specific to maternal sepsis and to our knowledge neither has been thoroughly evaluated to assess for impact in increasing awareness.

This evaluation looked at recognition and exposure to the GLOSS campaign materials and changes in provider awareness of maternal sepsis after campaign implementation. The latter included changes in knowledge on maternal sepsis and perception of enabling environments for identification and management of maternal sepsis.

METHODS

The GLOSS campaign was designed to accompany the Global Maternal Sepsis Study with the goal of raising awareness on maternal sepsis among healthcare providers working in participating facilities. Details regarding study protocol, including selection of countries and facilities was published elsewhere.[12] In short, GLOSS was a facility-based, one-week inception cohort study which enrolled pregnant or recently pregnant women with suspected or confirmed infection at 713 healthcare facilities in pre-specified geographical areas located in 52 low-, middle-, and high-income countries.[12]

The STOP SEPSIS! awareness campaign

The campaign launch was planned for before study implementation continuing throughout data collection and beyond. It was designed using existing frameworks for public information campaigns, social marketing, health communication, and behaviour change.[13,18–20] The development of the campaign included an overarching communication strategy using a multi-component approach delivering a simple and consistent message through visually-attractive media.[21]

For peer review only - http://bmjopen.bmj.com/site/about/guidelines.xhtml

The campaign had a soft launch with an online congress on 12 September 2017 and the full campaign rollout began on 06 November 2017, which included a website, printed materials, social media messaging, press releases. While global coordination of the campaign was undertaken by WHO, implementation of the campaign was the remit of GLOSS country coordinators. **Box 1** describes the different actions and components that were necessary for the design and development of the *STOP SEPSIS! awareness campaign*.

Box 1. Actions and components for the *STOP SEPSIS! awareness campaign*

- *Select a campaign lead.* A campaign lead was selected to coordinate and assist with the development of the campaign strategy and execution, and evaluation plan at a global level. This person ensured execution of each of the steps, supported the communication company and the study country coordinators in the participating countries who interacted with the providers working in the participating facilities.
- *Agree on a budget to fund the campaign*. Funds were necessary to cover the costs of the campaign lead, the communication company, and support to countries for printing of materials. The cost of this campaign was USD 200,000.
- *Seek the assistance of health and media communication experts*. A communication company with expertise in global health was contracted to lead the design and development of the GLOSS campaign concept and look.
- *Decide on the minimum set of materials and activities to be developed and implemented*. With input from people in the field who would be targeted through the campaign, the decision to have posters, infographics, press release and other presentation templates, social media messaging, and a website was agreed upon. In addition, a global congress was conceived in collaboration with partners from the Global Sepsis Alliance.
- *Develop campaign messaging, image, and logo*. A main message, tagline, and logo were designed with assistance from the communication company, content experts in maternal sepsis, and country/regional coordinators for GLOSS.
- *Develop an evaluation plan*. Given the breadth and geographical extent of the campaign's target population an online survey was used to collect providers' knowledge, attitudes, practices at baseline and post-campaign, including additional measures of campaign recognition and exposure at post-campaign. Paper-based surveys were used on demand.
- *Support the printing and upkeep of materials*. The campaign lead coordinated translation of all materials into five UN official languages and three additional languages as per GLOSS country coordinators' request. Participating countries were provided with funds needed to print the posters and infographics. Campaign lead was also in charge of regular upkeep of the dedicated website which includes timely news stories.
- *Implement the campaign*. This included:
    - *WSC Spotlight Congress*. A free, online congress focusing specifically on maternal and neonatal sepsis offered in collaboration with the Global Sepsis Alliance (https://wscspotlight.org/). The 25 presentations given over four sessions were later made available as YouTube videos and podcasts for free, with subtitles in multiple languages.

For peer review only - http://bmjopen.bmj.com/site/about/guidelines.xhtml

- ○ *Website*. A dedicated website used both as a repository of campaign materials for free download and to disseminate news about the study (http://srhr.org/sepsis).
- ○ *Print materials*. Posters with information about the study and infographics on maternal sepsis prevention, and identification and management to be displayed in different areas where women with suspected or confirmed infection could be found (e.g. labour ward, patient waiting area).
- ○ *Press releases*. Templates for announcing the objectives of the study and the campaign; countries/facilities were encouraged to engage local media for this purpose.
- ○ *Social media*. Campaign messaging disseminated and multiplied using social media through HRP's Twitter platform (@HRPresearch).

- *Expand the effect of the campaign*. Countries were encouraged to take ownership over the campaign and develop additional materials and organise activities prior to the start of study data collection.

Evaluation of the STOP SEPSIS! awareness campaign

We used an independent sample pre-post intervention design through online and paper-based surveys. Details regarding the definition used for awareness for this campaign, survey formulation and dissemination, including analysis of baseline data have been published elsewhere.[22] Briefly, a pre-campaign 32-question survey was developed to gather baseline information on healthcare providers' awareness of maternal sepsis through self-reported knowledge on maternal sepsis and perception of their work environments as enabling for the identification and management of maternal sepsis. Knowledge was assessed through questions relating to whether respondents had heard of maternal sepsis, correct identification of criteria that define maternal sepsis (infection plus organ dysfunction), and identification of correct initial management of maternal sepsis and infections (antibiotics and fluids) when maternal sepsis was suspected in the case vignette presented in the survey. Perception of enabling environments was assessed through self-reported confidence in making right decisions, reported availability of resources for correct identification and management, and feeling of support from their work environments in dealing with maternal sepsis, using a five-point Likert-scale. The same survey was administered at post-campaign to assess changes in knowledge and perception of their environments; 14 additional questions were included in the post-

For peer review only - http://bmjopen.bmj.com/site/about/guidelines.xhtml

survey which considered respondents' recognition of and exposure to the campaign, such as knowledge about the study and the campaign, message recall, engagement with social media for the campaign, and whether the campaign materials prompted changes in behaviour. See **Appendix 1** for a copy of the surveys.

Eligible respondents were healthcare providers working in GLOSS participating facilities in countries that received financial support for campaign implementation (N=46); we excluded all surveys from respondents that did not explicitly state that they were providers caring for women with infections in healthcare facilities (e.g., hospital administrators, physical therapists, or community health workers, or if the field was left blank) and from countries with less than two responses at either pre- or post-campaign (N=9). See **Figure 1** for a map of all the countries eligible for this evaluation. The surveys were distributed using a snowballing technique and were available in eight languages: Arabic, English, French, Italian, Portuguese, Russian, Spanish, and Vietnamese. The surveys were available for over 30 days (pre-campaign between 29 September and 05 November 2017, post-campaign between 31 January and 11 March 2018). Weekly reminders were sent through the online tool and via email to non-respondents. Targeted outreach was undertaken in countries with fewer than two responses. The campaign was active between 06 November 2017 and 15 January 2018; however, countries were encouraged to continue to use the materials beyond GLOSS study implementation. Ethical approval for GLOSS and the awareness campaign was obtained from WHO's Ethics Review Committee (protocol ID A65787) and from local and facility ethics committees as necessary.

Data analysis

We used descriptive analysis to provide frequencies and percentages for the characteristics of the sample, knowledge and perceived enabling environments, and for all the questions relating to campaign recognition and exposure. The latter was assessed through post-campaign surveys only

For peer review only - http://bmjopen.bmj.com/site/about/guidelines.xhtml

and complemented with self-reported accounts by GLOSS country coordinators. Text-based responses were codified into numerical values according to common emerging themes. All Likert-scale answers were dichotomized assigning a 1 to the two most favourable responses (i.e., they felt *very* or *somewhat* confident about making the right decision) and 0 to the combination of remaining options (*neutral*, *not very confident* or *not confident at all*). While previously we assessed dichotomization using 1 to the single most favourable response (i.e., respondent felt *very confident*) and a 0 to the combination of remaining options (*somewhat confident*, *neutral*, *not very confident* or *not confident at all*)[22] we decided to include a more flexible definition of confidence, perception of availability of resources, and feeling of support to allow for a more robust denominator that would enable comparisons. See **Appendix 2** for results of the overall analysis using this second dichotomization not used in this evaluation.

To assess impact of the campaign we conducted several analyses. First, we calculated percentage change ([(% in post - % in pre)/% in pre]x100) and estimated odds ratios (ORs) to determine differences in respondent knowledge and perception of enabling environments after campaign implementation relative to baseline measure for the total sample and by respondent characteristics. Due to the methodology used for survey dissemination and anonymity of surveys, this was not a matched sample, paired response pre-post analysis. Sensitivity analyses were conducted restricting the population to facilities from which we received at least one survey response and at least two responses, and to countries with more than 30 responses per country at pre- and at post-campaign survey.

Second, we used multivariate logistic regression models to explore the association between respondents' and facilities' characteristics at pre- and post-campaign and change in components of awareness after campaign implementation. Based on analysis of baseline data and our assumptions on characteristics that would be associated with levels of awareness,[22] we included the following

For peer review only - http://bmjopen.bmj.com/site/about/guidelines.xhtml

variables in the model: whether respondent was a physician, years of work experience, region where the respondent worked, whether the country had implemented an *expanded* version of the campaign, and whether the facility was a level III facility. Countries were considered to have implemented an expanded version of the campaign if they had not only printed and displayed all posters and infographics, prepared and disseminated press releases, but if they had also organized other activities or developed other materials for the campaign. Since less than 20% of respondents participated in the World Sepsis Congress Spotlight we did not include this variable in our models. We looked at effect modification by examining interactions between the time of the survey (pre- or post-) and each of the characteristics included in the model.

We used Pearson's χ^2 test to compare proportions and Wald's test to assess for significant differences in the models including interaction terms. Logistic regression analysis was used to estimate odds ratios between pre- and post-, crude and adjusted, clustering at the geographical area level. Statistical significance is reported at $p<0.05$. Stata (version 14.2, College Station, TX) was used for the analyses.

Patient and public involvement

This research was done without patient or public involvement. While the development of the campaign was done with input from study regional and country coordinators, respondents to the surveys were not invited to comment on the study design or to contribute to the writing of this manuscript given their anonymity.

Role of the funding source

The funders of the study had no role in study design, data collection, data analysis, data interpretation, or writing of this original article.

RESULTS

For peer review only

For peer review only - http://bmjopen.bmj.com/site/about/guidelines.xhtml

A total of 2,188 surveys met our inclusion criteria. Of these, 1,155 from 192 facilities were received at baseline and 1,033 from 196 facilities at post-campaign. There were no significant differences in sociodemographic characteristics between respondents at pre- and post-campaign surveys, except for a higher proportion of respondents working in a public facility at post-campaign and the higher proportion of respondents from countries where an expanded version of the campaign was implemented (**Table 1**). Responses came from the same overall countries at pre- and post-campaign. Because of the technique used for survey dissemination and because we did not know the total population of potentially exposed healthcare providers working in GLOSS participating facilities (provider turnover, rotation, and replacements is high), we were unable to calculate a response rate. However, since the campaign was implemented equally at the geographical area level, if providers remained within the study area they would have been exposed to the campaign. Results from the sensitivity analyses showed that overall findings in the sub-groups considered were consistent with the results from the complete sample (**Appendix 3**); for this reason, we used the entire sample for all subsequent analyses.

For peer review only

Table 1. Characteristics of respondents and the facilities in which they work at pre- and post-campaign survey (N=2,188)

Respondent characteristics	Pre-campaign (N=1,155)		Post-campaign (N= 1,033)	
	N	%	N	%
Age (in years)	*1,147*		*1,020*	
<31	354	31	301	30
31-40	389	34	407	40
>40	404	35	312	31
Sex	*1,153*		*1,022*	
Male	287	25	223	22
Female	866	75	799	78
Qualification	*1,151*		*1,025*	
Nurse/auxiliary nurse/midwife	440	38	456	44
Physician	561	49	456	44
Resident	150	13	113	11
Years of work experience	*1,107*		*970*	
<10	541	49	476	49
10-20	349	32	320	33

For peer review only - http://bmjopen.bmj.com/site/about/guidelines.xhtml

>20		217	20		174	18
Region	*1,155*			*1,033*		
Africa		224	19		226	22
Asia		173	15		170	16
Eastern Mediterranean		171	15		165	16
Europe‡		137	12		97	9
Latin America		450	39		375	36
Level of the facility in which respondent works	*1,153*			*1,033*		
I		127	11		166	16
II		236	20		258	25
III		790	69		609	59
*Respondent worked in a public facility**	*1,154*			*1,033*		
Yes		937	81		928	90
No		217	19		105	10
*Country implemented an expanded version of campaign**	*1,155*			*1,033*		
Yes		705	39		533	52
No		450	61		500	48

‡*Includes countries in Central Asia (Kazakhstan, Kyrgyzstan, and Tajikistan), in line with WHO regions*

**p<0.05*

We first present the results relating to campaign recognition and exposure and then results relating to changes in knowledge and perception of respondents' work environments.

Campaign recognition and exposure

Campaign recognition and exposure were high among most of the post-campaign survey respondents. Seventy-six percent of respondents stated they noticed the materials in their facilities; among those, 94% reported finding the materials helpful, 90% that the materials helped increase awareness on maternal sepsis and 88% that the materials motivated them to do something differently. Only 8% of respondents had used Twitter to amplify the message of the campaign (**Figure 2**). Among respondents that stated that the information provided in the materials motivated them to do something differently than before, 83% stated that it motivated them to suspect maternal sepsis and

For peer review only - http://bmjopen.bmj.com/site/about/guidelines.xhtml

For peer review only

77% to act fast. (**Figure 3**). Among respondents stating that the materials had not motivated them to do anything differently, 45% said it was because they already knew about maternal sepsis identification and management while 12% stated they had not seen the campaign materials.

Country coordinators shared anecdotal experiences of increased awareness in their facilities and the implementation of changes in practice and policies because of the study and the campaign. These accounts speak of a broader engagement with maternal sepsis identification and management. See **Box 2** for some examples.

Box 2. Accounts from the field*

Implementation of the campaign changed the way the city's providers acted. First, it helped in bridging the gap between academics and providers, which, in turn, helped motivate the entire staff around the study. The campaign helped us all feel more committed with the study. And, most importantly, it helped shed light on a problem (maternal sepsis) that we hadn't made public before. (Cali, Colombia)

In (our) facility there was already a protocol for sepsis early recognition, but the campaign, as well as the study made it come alive again. Sepsis was on everyone's eyes and mouths. The teams were very permeable to knowledge and eager to recognize and treat sepsis immediately. (Campinas, Brazil)

Participation in the campaign allowed me to see that we can find cases of maternal sepsis in the most diverse locations in a facility. And that invariably the most complex cases were those resulting from a condition that was neglected or treated incorrectly/untimely. (Maputo, Mozambique)

Despite having some protocols in place, during the campaign and study we realized that these were not sufficient to detect women with infection. This campaign was very important and helped us find a lot of cases that might have been missed otherwise (...) We are planning on improving reporting mechanisms of any suspected cases and supportive supervision and surveillance as a result of this study. (Ulaanbaatar, Mongolia)

As a result of our participation in GLOSS, we actually committed as a Program in our 2017 Maternal Death Review Forum to eliminate maternal sepsis as a cause of maternal death. (Manila, Philippines)

(Since implementing the GLOSS awareness campaign at a national level, we noticed that) we have prioritized the identification and suspicion of maternal and neonatal sepsis in all level I facilities, in specialized hospital care, and in the public health agenda. (Mexico City, Mexico)

* These reports first appeared in a blog post on the Merck for Mothers website in April 2018: https://www.msdformothers.com/blog/assessing-addressing-maternal-sepsis.html and in a news story on WHO/HRP's website in September 2018: https://www.who.int/reproductivehealth/maternal-sepsis-mexico/en/

For peer review only

For peer review only - http://bmjopen.bmj.com/site/about/guidelines.xhtml

Knowledge on maternal sepsis and perception of enabling environments

At pre-campaign survey, 92% of respondents (1,049/1,144) had heard of maternal sepsis. However, only 16% (109/673) of respondents were able to correctly identify the definition criteria of maternal sepsis and 45% (114/251) identified the correct management for maternal sepsis. In addition, at pre-campaign, most survey respondents stated that their work environments were enabling for maternal sepsis identification and management: 78% (897/1,155) stated that they felt confident of making right decisions, 79% (909/1,155) that they perceived resources were available, and 80% (921/1,155) that they felt supported by their facilities. See **Table 2** for overall results.

After campaign implementation there was a significant decrease in respondents who stated not having heard of maternal sepsis (-63.4% change; OR 0.35, 95% CI 0.18-0.68). There was also a significant increase in perceived confidence in making right decisions with regards to maternal sepsis identification and management (7.3% change; OR 1.44, 95% CI 1.01-2.06), although this level was quite high at pre-campaign (78%). There was a slight increase in respondents' ability to identify the correct management when maternal sepsis was suspected after the implementation of the campaign, but this was not statistically significant (30.8% change; OR 1.76, 95% CI 0.73-4.21). See **Appendix 4a and 4b** for these results according to respondent and facility characteristics.

For peer review only

Table 2. Respondent knowledge on maternal sepsis and perception of enabling environments for maternal sepsis identification and management at pre- and post-campaign and changes after campaign implementation (N=2,188)

	Pre-campaign n/N (%)	Post-campaign n/N (%)	Pre-post cOR‡ [95% CI]¶	Percentage change %
Knowledge on maternal sepsis				
Had not heard of maternal sepsis[(A)]	95/1,144 (8.3)	31/1,021 (3.0)	0.35* [0.18-0.68]	-63.4
Correctly identified the two criteria to define maternal sepsis[(B)]	109/673 (16.2)	74/647 (11. 4)	0.67 [0.43-1.17]	-29.4
Correctly identified management of sepsis when maternal sepsis was suspected[(C)]	114/251 (45.4)	142/239 (59. 4)	1.76 [0.73-4.21]	30.8

For peer review only - http://bmjopen.bmj.com/site/about/guidelines.xhtml

Perception of enabling environment for maternal sepsis identification and management				
Confident of making right decisions	897/1,155 (77.7)	861/1,033 (83.4)	1.44* [1.01-2.06]	7.3
Resources available to make right decisions	909/1,155 (78.7)	814/1,033 (78.8)	1.01 [0.68-1.49]	0.1
Supported by facility in making right decisions	921/1,155 (79.7)	840/1,033 (81. 3)	1.11 [0.80-1.54]	2.0

cOR: crude odds ratio; CI: confidence interval
Percentage change: [(% in post - % in pre)/% in pre]x100
‡Refers to odds ratio between pre- and post-campaign; OR calculated clustering at the geographical area level
¶reference group: pre-campaign; **p<0.05*

(A)Responded NO to the question "have you ever heard of the term maternal sepsis?"
(B) Answered INFECTION and ORGAN DYSFUNCTION to the question: "what two criteria best describe maternal sepsis?"
(C) Answered FLUIDS and ANTIBIOTICS to the question: "what would be the first two things a woman should receive," when the respondent answered INFECTION/SEPSIS to the question: "what would you first think could be causing her to feel this way?"

After controlling for respondent and facility characteristics, being a physician, having less than 10 years of experience, and working in a level III facility were associated with decreased odds of not having heard of maternal sepsis at pre-campaign (**Table 3**). Respondents from facilities that had implemented an expanded version of the campaign were more likely to have heard of maternal sepsis and identify the correct management of maternal sepsis at post-campaign. Respondents with less than 10 years of experience were more likely to have heard of maternal sepsis at pre-campaign, but there were no differences across providers with different years of experience after the campaign.

Physicians were more likely to respond that they felt confident in making the right decisions at post-campaign, while being a physician and having more than 20 years of experience had a significant interaction with time of the survey with regards to perception of availability of resources and support from their facilities. At pre- and post-campaign, respondents with 20 years or more of experience were more likely to perceive availability of resources for making right decisions and to feel supported by their facilities and these differences between groups were significant after the campaign (**Table**

For peer review only - http://bmjopen.bmj.com/site/about/guidelines.xhtml

4). No differences in the perception of enabling environments were seen among respondents from facilities that had implemented an expanded version of the campaign.

For peer review only

For peer review only - http://bmjopen.bmj.com/site/about/guidelines.xhtml

Table 3. Knowledge on maternal sepsis adjusted for respondents' characteristics (N=2,188)

	Had not heard about maternal sepsis					Correctly identified the two criteria to define maternal sepsis					Correctly identified management of sepsis when maternal sepsis was suspected				
	Pre-campaign		Post-campaign		Wald's test	Pre-campaign		Post-campaign		Wald's test	Pre-campaign		Post-campaign		Wald's test
Respondent characteristics	aOR	95% CI	aOR	95% CI	*p*	aOR	95% CI	aOR	95% CI	*p*	aOR	95% CI	aOR	95% CI	*p*
Physician	0.29*	0.10-0.85	0.58	0.17-1.93	0.108	1.78	0.80-3.95	3.85*	1.54-9.60	0.175	2.07*	1.24-3.45	2.81*	1.08-7.30	0.583
Years of work experience															
<10	0.50*	0.26-0.96	1.57	0.77-3.18	0.035	1.08	0.60-1.96	0.86	0.55-1.34	0.352	0.86	0.53-1.40	0.61	0.30-1.23	0.775
10-20	1 (ref)		1 (ref)			1 (ref)		1 (ref)			1 (ref)		1 (ref)		
>20	1.21	0.61-2.38	0.73	0.19-2.87		1.17	0.59-2.30	1.50*	1.02-2.21		1.59	0.60-4.22	0.9	0.30-2.71	
Country implemented an expanded version of campaign	1.54	0.46-5.12	0.21*	0.06-0.78	0.004	1.18	0.53-2.65	0.86	0.31-2.34	0.281	2.78*	1.01-7.59	8.02*	2.03-31.73	0.437
Respondent worked in a level III facility	0.45*	0.21-0.96	1.79	0.59-5.42	0.006	1.63	0.77-3.45	2.80*	1.32-5.94	0.314	2.10	0.85-5.16	1.14	0.43-3.02	0.406

‡Includes countries in Central Asia (Kazakhstan, Kyrgyzstan, and Tajikistan), in line with WHO regions

Countries were considered to have implemented an expanded version of the campaign if they had not only printed and displayed all posters and infographics, prepared and disseminated press releases, but if they had also organized other activities or developed other materials for the campaign.

Adjusting for whether respondent was a physician, years of work experience, region, whether the country implemented an expanded version of the campaign, and whether respondent worked in a level III facility, clustering at the geographical area level

*aOR: adjusted odds ratio; CI: confidence interval; Wald's test used to assess for differences in the models including interaction terms; *p<0.05*

For peer review only

For peer review only - http://bmjopen.bmj.com/site/about/guidelines.xhtml

Table 4. Perception of enabling environments for maternal sepsis identification and management adjusted for respondents' characteristics (N=2,188)

	Confident of making right decisions					Resources available to make right decisions					Supported by facility in making right decision				
	Pre-campaign		Post-campaign		Wald's test	Pre-campaign		Post-campaign		Wald's test	Pre-campaign		Post-campaign		Wald's test
Respondent characteristics	aOR	95% CI	aOR	95% CI	*p*	aOR	95% CI	aOR	95% CI	*p*	aOR	95% CI	aOR	95% CI	*p*
Physician	1.02	0.64-1.64	1.69*	1.19-2.40	0.246	0.81	0.55-1.20	1.39	0.99-1.95	0.021	0.78	0.55-1.09	1.26	0.83-1.92	0.017
Years of work experience															
<10	0.74	0.51-1.09	0.88	0.63-1.22	0.025	0.63*	0.41-0.97	0.82	0.53-1.27	0.014	0.70	0.46-1.06	0.95	0.69-1.30	0.038
10-20	1 (ref)		1 (ref)			1 (ref)		1 (ref)			1 (ref)		1 (ref)		
>20	1.20	0.76-1.91	2.54*	1.38-4.64		1.66*	1.08-2.54	2.48*	1.49-4.13		1.60*	1.07-2.40	2.78*	1.84-4.20	
Country implemented an expanded version of campaign	0.65	0.36-1.16	0.91	0.56-1.48	0.605	1.00	0.52-1.94	1.57	0.85-2.88	0.241	1.32	0.86-2.03	1.37	0.90-2.07	0.092
Respondent worked in a level III facility	0.90	0.55-1.47	0.64*	0.41-1.00	0.581	1.39	0.86-2.23	1.27	0.79-2.04	0.255	0.88	0.53-1.46	1.18	0.67-2.07	0.116

‡Includes countries in Central Asia (Kazakhstan, Kyrgyzstan, and Tajikistan), in line with WHO regions

Countries were considered to have implemented an expanded version of the campaign if they had not only printed and displayed all posters and infographics, prepared and disseminated press releases, but if they had also organized other activities or developed other materials for the campaign.

Adjusting for whether respondent was a physician, years of work experience, region, whether the country implemented an expanded version of the campaign, and whether respondent worked in a level III facility, clustering at the geographical area level

*aOR: adjusted odds ratio; CI: confidence interval; Wald's test used to assess for differences in the models including interaction terms; *p<0.05*

241

For peer review only - http://bmjopen.bmj.com/site/about/guidelines.xhtml

DISCUSSION

To the best of our knowledge, this is the first study to assess impact of an awareness campaign aimed at healthcare providers and implemented at a global level where pre-campaign and post-campaign data were collected in addition to measures relating to campaign recognition and exposure. Most healthcare providers stated that the campaign helped increase awareness of maternal sepsis and motivated them to do something differently, particularly to suspect maternal sepsis and act faster. Reports from the field also support this finding that exposure to the campaign increased sensitization to maternal infections and sepsis. Moreover, most survey respondents had heard of maternal sepsis even before campaign implementation; after the campaign this increased significantly. Although most respondents perceived their enabling environments in a positive way before campaign implementation, there was an increase in respondent confidence to make the right decisions regarding maternal sepsis identification and management after campaign implementation.

The *STOP SEPSIS! awareness campaign* implementation was effective with regards to respondents' recognition of and exposure to the campaign; other campaign evaluations have used these measures to positively assess short-term impact of campaigns.[23–25] Furthermore, consistent and repeat exposure to campaign messaging have shown to increase awareness;[13] while exposure was only measured over the course of this evaluation period corresponding to the intended implementation period of the campaign, the fact that most respondents stated the campaign raised awareness is a promising trend in the right direction.

Overall knowledge about maternal sepsis increased from pre- to post-campaign implementation among respondents to our survey with regards to having heard about maternal sepsis. Our finding that overall knowledge increased is supported by existing literature that suggests that campaigns can increase knowledge on a specific topic among healthcare providers[14,26,27] as well as among the general population.[23,28,29] The fact that there was a slight increase in identifying the correct

For peer review only - http://bmjopen.bmj.com/site/about/guidelines.xhtml

management of maternal sepsis is important. Research has shown that knowing what is needed to manage maternal sepsis correctly and early management of maternal sepsis are critical to implementing any changes in providers' behaviour and improving maternal health outcomes.[6,30,31] The low number of providers able to identify the two criteria defining maternal sepsis might be more a reflection of the lack of consensus on this condition prior to 2017, rather than a shortcoming of the campaign.[32] The GLOSS awareness campaign was associated with reducing differences among groups of healthcare providers depending on their qualifications or years of experience. This speaks to the importance of including healthcare providers with different qualifications and years of experience in awareness-raising efforts.

We found there were overall increases in respondent confidence in making right decisions about maternal sepsis identification and management, but no significant changes with regards to overall respondent perception of availability of necessary resources and feeling supported by their facilities. Evidence shows that confidence can not only affect clinical performance;[33] but also that high levels of confidence among healthcare providers can have a positive impact on patients' perception of experience of care.[34] However, the change in perception of availability of resources and support limited to physicians and more experienced providers raises a broader question on actions that facilities need to take to empower all healthcare workers in feeling that they have the necessary resources and feel supported to provide quality care. This is especially important if we consider that a more restrictive definition of enabling environments results in much lower overall levels of perceived confidence, perception of availability of resources, and feeling of support. Perceived lack of availability of resources may also be a product of increased awareness of what is necessary to address maternal sepsis. These findings are a call to hospital administrators and policy-makers to foster enabling environments and secure availability and access to life-saving resources.

For peer review only - http://bmjopen.bmj.com/site/about/guidelines.xhtml

289 Sepsis awareness is gaining traction on global agendas;[4,10,16] this is supported by evidence from two studies looking at internet searches on sepsis,[35,36] meaning increases resulting from this campaign could be responding to natural trends or other factors. It is also possible that awareness was raised by having participated in the research study and not necessarily because of the campaign; disentangling the effect of the campaign from that of the implementation of the research study was impossible. Understanding whether any of these changes are sustained over time would provide us with further information on the lasting effects of the campaign.

296 Literature shows that while a campaign can help in raising awareness, it is insufficient in allowing for changes in behaviour.[13,19] While behaviour change is important in impacting population-level health, it is one of many components needed to make significant improvements; evidence from this study, similar to others, highlight the need for health systems improvements such as availability of critical resources and support to improve maternal outcomes.[37] Assessing the impact that increased awareness resulting from a campaign has on behaviour change would provide us with supporting evidence that campaigns can help in improving health outcomes.

303 This study has some limitations. First, we used a pre-post design methodology with no control group which does not allow to discern the impact of the campaign alone. Second, the method used to disseminate the survey and the fact that surveys were anonymous made it impossible to match responses at pre- and post-campaign. Surveys were anonymous to encourage providers to respond and remove potential response bias. However, it is to note that characteristics of participants at pre- and post-campaign were similar. Third, because implementation of the campaign was left up to country coordinators, campaign fidelity was only assessed through healthcare provider self-report at post-campaign surveys. Fourth, this evaluation was restricted to the duration of the study follow-up period, hence providing insight into early findings only and limiting our knowledge of lasting impact of the campaign, which was beyond the goal of this activity. However, our findings suggest

For peer review only - http://bmjopen.bmj.com/site/about/guidelines.xhtml

that campaigns can have at least short-term effects on provider's knowledge and confidence. The positive perception of the campaign materials is encouraging. And fifth, since baseline data was collected after the soft launch of the campaign, the effect of the campaign may have been minimized because awareness had already been increased through exposure to the online congress as well as other global activities on sepsis conducted by other groups. However, we know that less than 20% of respondents participated in the congress.

Our findings have implications for both practice and research. On the one hand, there appear to be benefits to coupling large multi-country studies with awareness campaigns. A campaign targeting healthcare providers can promote their engagement with research studies being conducted, potentially improving study outcomes. There is also evidence that including an awareness campaign creates an environment prime to implementing changes to clinical practice as per research study protocol. On the other hand, there is a clear need for additional research to identify lasting effects of awareness campaigns, especially as global initiatives focus on increasing awareness on maternal health issues.

A campaign designed to raise awareness among healthcare providers working in facilities participating in a global research study was associated with an increase in having heard of maternal sepsis, as well as increased provider perception of confidence in making correct decisions. Offering healthcare providers with the information to make accurate and timely decisions while promoting environments that enable self-confidence and support could improve maternal sepsis identification and management, which can ultimately have an impact on maternal health outcomes.

FIGURES

Figure 1. Countries eligible for the GLOSS '*STOP SEPSIS!*' awareness campaign evaluation (N=46)

For peer review only

For peer review only - http://bmjopen.bmj.com/site/about/guidelines.xhtml

Figure 2. Measures of campaign exposure in percentages (N=1,033)

Figure 3. Responses when answering YES to the question "Did the information provided in the materials motivate you to do something differently than before?" (N=668). (Respondents were able to check as many response options as needed)

APPENDICES

Appendix 1. Global Maternal Sepsis Study – Pre- and Post- Campaign Surveys

Appendix 2. Respondent perception of enabling environments for maternal sepsis identification and management at pre- and post-campaign and changes after campaign implementation, limiting to most favourable responses (N=2,188)

Appendix 3. Changes in respondent knowledge and perception of enabling environments after campaign implementation among different sub-groups

Appendix 4. Changes in respondent knowledge and perception of enabling environments after campaign implementation according to respondent and facility characteristics

Acknowledgments: The authors wish to thank Khalid Yunis for his contributions to the development of the campaign, Soe Soe Thwin and Cristina Cuesta for their assistance in the planning and review of statistical analyses, and all the respondents to the online and paper-based survey. The authors would also like to acknowledge the contribution and lifelong achievements of co-author Bukola Fawole, who passed away prior to the publication of this article.

For peer review only

For peer review only - http://bmjopen.bmj.com/site/about/guidelines.xhtml

Contributors: The evaluation plan and first draft of this original article was conceived by VB. MB provided substantial contributions to the data planning and analysis and first versions of this manuscript. The idea for the inclusion of an awareness campaign was first conceived by JPS. CLTR, JPS, and EA provided additional feedback on first draft and data analysis. VB, MB, JPS, EA, AB, AOF, MK, PL, MM, AN, NBO, ZQ were instrumental to campaign design and implementation in their regions/countries and provided input to data collection tools used for this evaluation. All authors read and approved the final version. The WHO GLOSS Research Group contributed to different aspects of the study, from protocol development to acquisition of data and coordination of study in their countries. VB, MB, and CLTR had full access to all the data and VB has final responsibility for the decision to submit for publication.

Funding: This work was supported by the UNDP/UNFPA/UNICEF/WHO/World Bank Special Programme of Research, Development and Research Training in Human Reproduction (HRP), Department of Sexual and Reproductive Health and Research, World Health Organization, Geneva, Switzerland (project A65787), Merck Sharp & Dohme Corp., a wholly owned subsidiary of Merck and Co., Inc. (Kenilworth, NJ USA), through its Merck for Mothers program, and the United States Agency for International Development (USAID) Grant number GHA-G-00-09-00003. The views of the funding bodies have not influenced the content of this manuscript.

Competing interests: All authors have completed the ICMJE uniform disclosure form at www.icmje.org/coi_disclosure.pdf and declare: support from WHO/SHR, Merck for Mothers, and USAID for the submitted work; VB, MB, and JPS were employed by WHO at the time of the study; no other relationships or activities that could appear to have influenced the submitted work.

For peer review only - http://bmjopen.bmj.com/site/about/guidelines.xhtml

Ethical approval: Ethical approval for the entire study, including the awareness campaign, was obtained from WHO's Ethics Review Committee in Geneva, Switzerland (protocol ID A65787) for a multi-site, multi-country project. A legend was included in the survey stating that response to the survey implied consent to participate; respondents could recuse themselves at any point during the survey, including skipping and not answering certain questions.

Data sharing: All enquiries regarding the data and analyses can be made to the corresponding author.

Transparency: The lead author (VB) affirms that the manuscript is an honest, accurate, and transparent account of the study being reported; that no important aspects of the study have been omitted; and that any discrepancies from the study as planned have been explained.

Disclosure: This article represents the views of the named authors only and does not represent the views of the World Health Organization

World Health Organization Global Maternal Sepsis Study Research Group (in alphabetical order)

AFGHANISTAN: Mohammad Iqbal Aman, Bashir Noormal. ARGENTINA: Virginia Díaz, Marisa Espinoza, Julia Pasquale. BELGIUM: Charlotte Leroy, Kristien Roelens, Griet Vandenberghe. BENIN: M. Christian Urlyss Agossou, Sourou Goufodji Keke, Christiane Tshabu Aguemon. BOLIVIA: Patricia Soledad Apaza Peralta, Víctor Conde Altamirano, Rosalinda Hernández Muñoz. BRAZIL: José Guilherme Cecatti, Carolina C Ribeiro do Valle. BURKINA FASO: Vincent Batiene, Kadari Cisse, Henri Gautier Ouedraogo. CAMBODIA: Cheang Kannitha, Lam Phirun, Tung Rathavy. CAMEROON: Elie Simo, Pierre-Marie Tebeu, Emah Irene Yakana. COLOMBIA: Javier Carvajal, María Fernanda Escobar,

For peer review only

For peer review only - http://bmjopen.bmj.com/site/about/guidelines.xhtml

Paula Fernández. DENMARK: Lotte Berdiin Colmorn, Jens Langhoff-Roos. ECUADOR: Wilson Mereci, Paola Vélez. EGYPT: Yasser Salah Eldin, Alaa Sultan. ETHIOPIA: Abdulfetah Abdulkadir Abdosh, Alula M. Teklu, Dawit Worku Kassa. GHANA: Richard Adanu, Philip Govule, Charles Noora Lwanga. GUATEMALA: William Enrique Arriaga Romero, María Guadalupe Flores Aceituno. HONDURAS: Carolina Bustillo, Rigoberto Castro, Bredy Lara. INDIA: Vijay Kumar, Vanita Suri, Sonia Trikha. ITALY: Irene Cetin, Serena Donati, Carlo Personeni. KAZAKHSTAN: Guldana Baimussanova, Saule Kabylova, Balgyn Sagyndykova. KENYA: George Gwako, Alfred Osoti, Zahida Qureshi. KYRGYZSTAN: Raisa Asylbasheva, Aigul Boobekova, Damira Seksenbaeva. LEBANON: Faysal El Kak, Saad Eddine Itani, Sabina Abou Malham. LITHUANIA: Meilė Minkauskienė, Diana Ramašauskaitė. MALAWI: Owen Chikhwaza, Luis Gadama, Eddie Malunga. MALI: Haoua Dembele, Hamadoun Sangho, Fanta Eliane Zerbo. MEXICO: Filiberto Dávila Serapio, Nazarea Herrera Maldonado, Juan Ismael Islas Castañeda. REPUBLIC OF MOLDOVA: Tatiana Cauaus, Ala Curteanu, Victor Petrov. MONGOLIA: Yadamsuren Buyanjargal, Seded Khishgee, Bat-Erdene Lkhagvasuren. MOROCCO: Bouchra Assarag, Amina Essolbi, Rachid Moulki. MOZAMBIQUE: Zara Jaze, Arlete Mariano, Nafissa Bique Osman. MYANMAR: Hla Mya Thway Einda, Thae Maung Maung, Khaing Nwe Tin. NEPAL: Tara Gurung, Amir Babu Shrestha, Sangeeta Shrestha. NETHERLANDS: Kitty Bloemenkamp, Marcus J. Rijken, Thomas Van Den Akker. NICARAGUA: María Esther Estrada, Néstor J. Pavón Gómez. NIGERIA: Olubukola Adesina, Chris Aimakhu, Bukola Fawole. PAKISTAN: Rizwana Chaudhri, Saima Hamid, M. Adnan Khan. PERU: María del Pilar Huatuco Hernández, Nelly M. Zavaleta Pimentel. PHILIPPINES: Maria Lu Andal, Carolina Paula Martin, Zenaida Dy Recidoro. ROMANIA: Mihaela Budianu, Lucian Pușcașiu. SENEGAL: Léopold Diouf, Dembo Guirassy, Philippe Marc Moreira. SLOVAKIA: Miroslav Borovsky, Ladislav Kovac, Alexandra Kristufkova. SOUTH AFRICA: Sylvia Cebekhulu, Laura Cornelissen, Priya Soma-Pillay. SPAIN: Vicenç Cararach, Marta López, María José Vidal Benedé. SRI LANKA: Hemali Jayakody, Kapila Jayaratne, Dhammica Rowel. SUDAN: Mohamed Elsheikh, Wisal Nabag, Sara Omer. TAJIKISTAN: Victoria Tsoy, Urunbish Uzakova, Dilrabo Yunusova. THAILAND: Thitiporn

For peer review only

429 Siriwachirachai, Thumwadee Tangsiriwatthana. UNITED KINGDOM: Catherine Dunlop, Marian
430 Knight, David Lissauer. URUGUAY: Aquilino M. Pérez, Jhon Roman, Gerardo Vitureira. VIET NAM:
431 Dinh Anh Tuan, Luong Ngoc Truong, Nghiem Thi Xuan Hanh. ZIMBABWE: Mugove Madziyire, Thulani
432 Magwali, Stephen Munjanja. Regional Coordinators: Edgardo Abalos, Adama Baguiya, Mónica
433 Chamillard, Bukola Fawole, Marian Knight, Seni Kouanda, Pisake Lumbiganon, Ashraf Nabhan, Ruta
434 Nadisauskiene. Technical Advisory Group: Linda Bartlett, Fernando Bellissimo-Rodrigues, Shevin T.
435 Jacob, Sadia Shakoor, Khalid Yunis. Data management and analysis: Liana Campodónico, Cristina
436 Cuesta, Hugo Gamerro, Daniel Giordano. WHO coordinating Unit: Fernando Althabe, Mercedes Bonet,
437 Vanessa Brizuela, A. Metin Gülmezoglu, João Paulo Souza.

For peer review only

For peer review only - http://bmjopen.bmj.com/site/about/guidelines.xhtml

References

1 Reinhart K, Daniels R, Kissoon N, *et al.* Recognizing Sepsis as a Global Health Priority — A WHO Resolution. *N Engl J Med* 2017;**377**:414–7. doi:10.1056/NEJMp1707170

2 Rudd KE, Kissoon N, Limmathurotsakul D, *et al.* The global burden of sepsis: barriers and potential solutions. *Crit Care* 2018;**22**. doi:10.1186/s13054-018-2157-z

3 Jawad I, Lukšić I, Rafnsson SB. Assessing available information on the burden of sepsis: global estimates of incidence, prevalence and mortality. *J Glob Health* 2012;**2**. doi:10.7189/jogh.02.010404

4 WHO. Improving the prevention, diagnosis and management of sepsis. World Health Assembly: 2017. http://apps.who.int/gb/ebwha/pdf_files/WHA70/A70_R7-en.pdf?ua=1

5 Surviving Sepsis Campaign | History. http://www.survivingsepsis.org/About-SSC/Pages/History.aspx (accessed 20 Aug 2017).

6 Rhodes A, Evans LE, Alhazzani W, *et al.* Surviving Sepsis Campaign: International Guidelines for Management of Sepsis and Septic Shock: 2016. *Intensive Care Med* 2017;**43**:304–77. doi:10.1007/s00134-017-4683-6

7 Levy MM, Evans LE, Rhodes A. The Surviving Sepsis Campaign Bundle: 2018 update. *Intensive Care Med* 2018;**44**:925–8. doi:10.1007/s00134-018-5085-0

8 Say L, Chou D, Gemmill A, *et al.* Global causes of maternal death: a WHO systematic analysis. *Lancet Glob Health* 2014;**2**:e323–e333.

9 Kassebaum NJ, Barber RM, Bhutta ZA, *et al.* Global, regional, and national levels of maternal mortality, 1990–2015: a systematic analysis for the Global Burden of Disease Study 2015. *The Lancet* 2016;**388**:1775–812. doi:10.1016/S0140-6736(16)31470-2

10 The Global Maternal and Neonatal Sepsis Initiative: a call for collaboration and action by 2030. *Lancet Glob Health* 2017;**0**. doi:10.1016/S2214-109X(17)30020-7

11 Singer M, Deutschman CS, Seymour CW, *et al.* The Third International Consensus Definitions for Sepsis and Septic Shock (Sepsis-3). *JAMA* 2016;**315**:801. doi:10.1001/jama.2016.0287

12 Bonet M, Souza JP, Abalos E, *et al.* The global maternal sepsis study and awareness campaign (GLOSS): study protocol. *Reprod Health* 2018;**15**:16. doi:10.1186/s12978-017-0437-8

13 Randolph W, Viswanath K. Lessons Learned from Public Health Mass Media Campaigns: Marketing Health in a Crowded Media World. *Annu Rev Public Health* 2004;**25**:419–37.

For peer review only

For peer review only - http://bmjopen.bmj.com/site/about/guidelines.xhtml

14 Pittet D, Hugonnet S, Harbarth S, *et al.* Effectiveness of a hospital-wide programme to improve compliance with hand hygiene. *The Lancet* 2000;**356**:1307–12. doi:10.1016/S0140-6736(00)02814-2

15 Glanz K, Rimer BK, Lewis, editors. *Health Behavior and Health Education: Theory, Research, and Practice*. 3rd ed. San Francisco: : Jossey-Bass 2002.

16 Global Sepsis Alliance. https://www.global-sepsis-alliance.org/ (accessed 27 Feb 2019).

17 UK Sepsis Trust. Sepsis Trust. https://sepsistrust.org/ (accessed 27 Feb 2019).

18 Weiss JA, Tschirhart M. Public Information Campaigns as Policy Instruments. *J Policy Anal Manage* 1994;**13**:82. doi:10.2307/3325092

19 Wakefield MA, Loken B, Hornik RC. Use of mass media campaigns to change health behaviour. *The Lancet* 2010;**376**:1261–71. doi:10.1016/S0140-6736(10)60809-4

20 *Making Health Communication Programs Work*. National Cancer Institute 2004. https://www.cancer.gov/publications/health-communication/pink-book.pdf (accessed 11 Jan 2017).

21 O'Connor M, McGowan K, Jolivet RR. An awareness-raising framework for global health networks: lessons learned from a qualitative case study in respectful maternity care. *Reprod Health* 2019;**16**. doi:10.1186/s12978-018-0662-9

22 Brizuela V, Bonet M, Souza JP, *et al.* Factors influencing awareness of healthcare providers on maternal sepsis: a mixed-methods approach. *BMC Public Health* 2019;**19**:683. doi:10.1186/s12889-019-6920-0

23 Kaufman MR, Harman JJ, Smelyanskaya M, *et al.* "Love me, parents!": impact evaluation of a national social and behavioral change communication campaign on maternal health outcomes in Tanzania. *BMC Pregnancy Childbirth* 2017;**17**:305. doi:10.1186/s12884-017-1470-x

24 Evans WD, Uhrig J, Davis K, *et al.* Efficacy methods to evaluate health communication and marketing campaigns. *J Health Commun* 2009;**14**:315–30. doi:10.1080/10810730902872234

25 Bazzo S, Black D, Mitchell K, *et al.* 'Too Young To Drink'. An international communication campaign to raise public awareness of fetal alcohol spectrum disorders. *Public Health* 2017;**142**:111–5. doi:10.1016/j.puhe.2016.08.001

26 Al-Dorzi HM, Cherfan A, Al-Harbi S, *et al.* Knowledge of thromboprophylaxis guidelines pre- and post-didactic lectures during a venous thromboembolism awareness day at a tertiary-care hospital. *Ann Thorac Med* 2013;**8**:165–9. doi:10.4103/1817-1737.114298

For peer review only

For peer review only - http://bmjopen.bmj.com/site/about/guidelines.xhtml

27 Pattinson RC, Bergh A-M, Makin J, *et al.* Obstetrics knowledge and skills training as a catalyst for change. *S Afr Med J* 2018;**108**:748. doi:10.7196/SAMJ.2018.v108i9.13073

28 Vallone D, Cantrell J, Bennett M, *et al.* Evidence of the Impact of the truth FinishIt Campaign. *Nicotine Tob Res* 2018;**20**:543–51. doi:10.1093/ntr/ntx119

29 Alaqeel A, Kamalmaz H, Abou Al-Shaar H, *et al.* Evaluating the initial impact of the Riyadh Epilepsy Awareness Campaign. *Epilepsy Behav* 2015;**52**:251–5. doi:10.1016/j.yebeh.2015.09.010

30 Robson W, Beavis S, Spittle N. An audit of ward nurses' knowledge of sepsis. *Nurs Crit Care* 2007;**12**:86–92. doi:10.1111/j.1478-5153.2007.00210.x

31 Gaieski D, Mikkelsen M, Band R, *et al.* Impact of time to antibiotics on survival in patients with severe sepsis or septic shock in whom early goal-directed therapy was initiated in the emergency department*. *Crit Care Med* 2010;**38**:1045–53. doi:10.1097/CCM.0b013e3181cc4824

32 World Health Organization. Statement on maternal sepsis. Published Online First: 2017.http://apps.who.int/iris/handle/10665/254608 (accessed 31 Mar 2017).

33 Cheng ER, Nelson CC, Leung-Strle P, *et al.* Nutrition provider confidence in the Massachusetts Childhood Obesity Research Demonstration (MA-CORD) study. *Prev Med Rep* 2019;**13**:289–92. doi:10.1016/j.pmedr.2019.01.010

34 Owens KM, Keller S. Exploring workforce confidence and patient experiences: A quantitative analysis. *Patient Exp J* 2018;**5**:10.

35 Jabaley CS, Blum JM, Groff RF, *et al.* Global trends in the awareness of sepsis: insights from search engine data between 2012 and 2017. *Crit Care Lond Engl* 2018;**22**:7. doi:10.1186/s13054-017-1914-8

36 Savelkoel J, Claushuis TAM, van Engelen TSR, *et al.* Global impact of World Sepsis Day on digital awareness of sepsis: an evaluation using Google Trends. *Crit Care* 2018;**22**. doi:10.1186/s13054-018-1981-5

37 van Den Broek, Nynke, Ameh C, Madaj B, *et al.* Effects of emergency obstetric care training on maternal and perinatal outcomes: a stepped wedge cluster randomised trial in South Africa. *BMJ Glob Health* Published Online First: 2019. doi:10.1136/ bmjgh-2019-001670

For peer review only

For peer review only - http://bmjopen.bmj.com/site/about/guidelines.xhtml

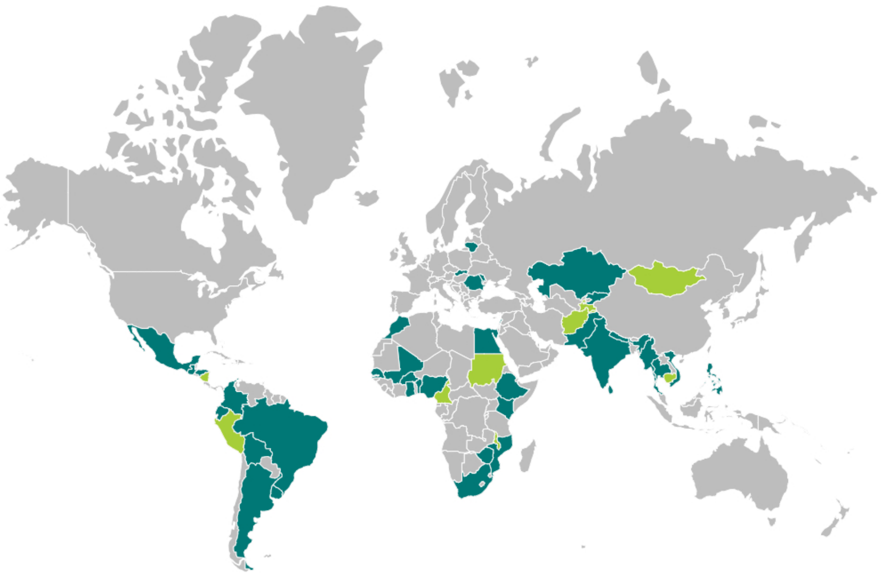

Figure 1. Countries eligible for the GLOSS ‘STOP SEPSIS!’ awareness campaign evaluation (N=46)
teal: countries included in the GLOSS STOP SEPSIS! awareness campaign evaluation (N=37);
green: countries eligible for the evaluation but from which 1 or less responses received for the evaluation (N=9).

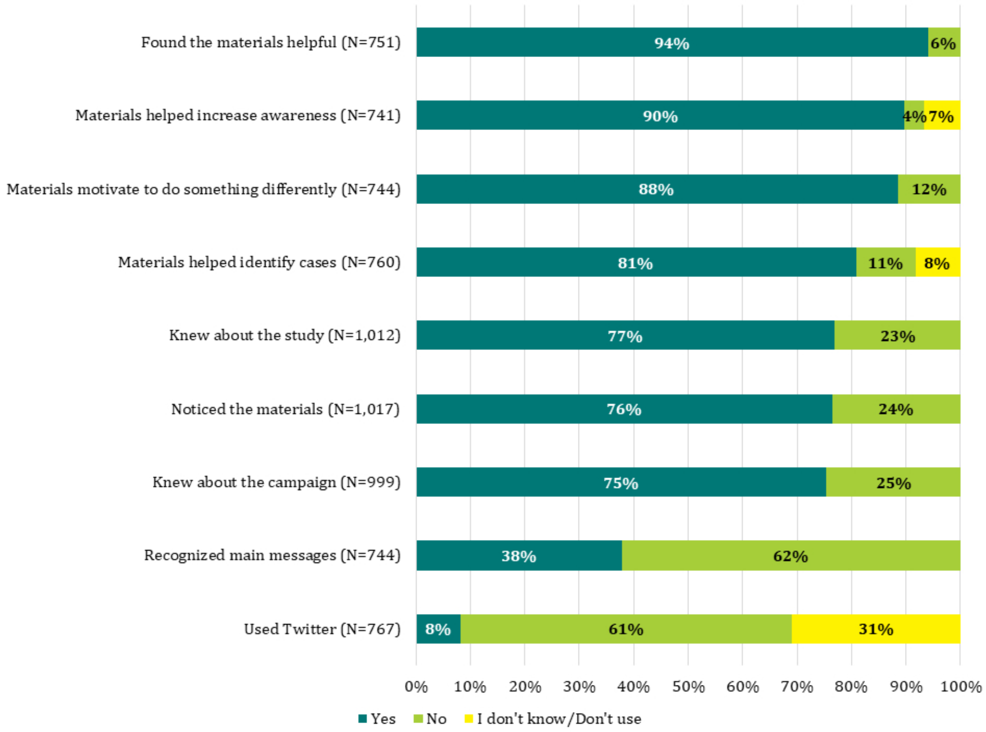

Figure 2. Measures of campaign exposure in percentages (N=1,033)

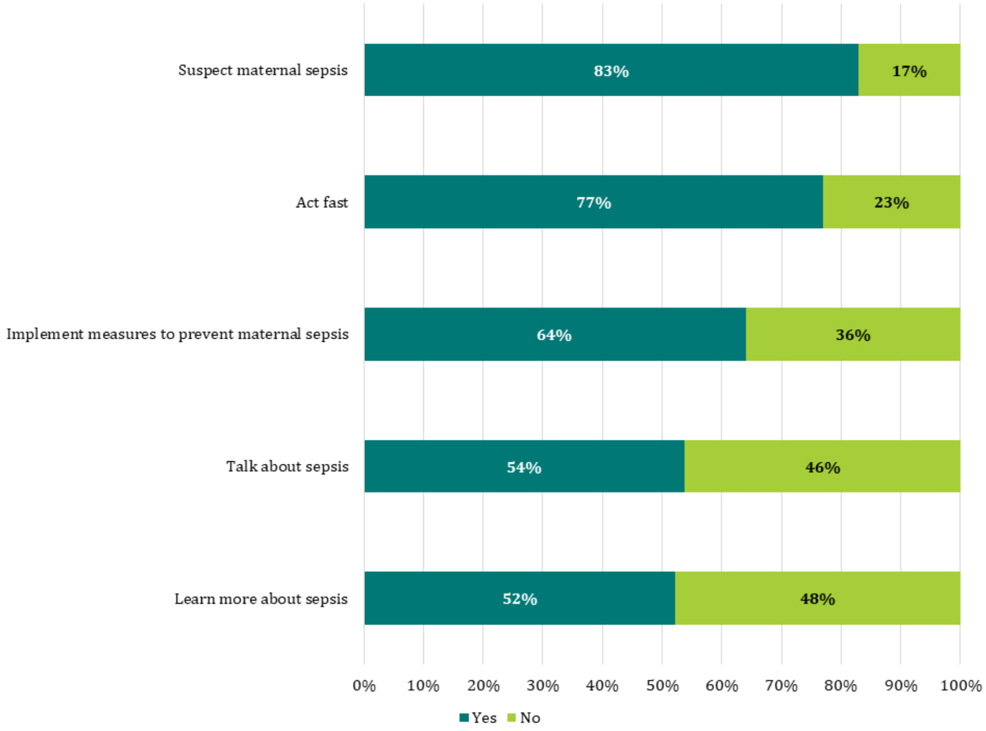

Figure 3. Responses when answering YES to the question "Did the information provided in the materials motivate you to do something differently than before?" (N=668). (Respondents were able to check as many response options as needed)

Appendix 1. Global Maternal Sepsis Study – Pre- and Post-Campaign Surveys

GLOSS Pre-Campaign Survey

This online survey is part of the activities set forth for a global study on maternal morbidity and mortality. This study is being conducted in approximately 50 countries across the globe, including your own, and it is coordinated by the World Health Organization and the healthcare facility where you work.

As part of this study, we want to learn more from healthcare providers about how you identify and manage women with complications during pregnancy, childbirth, postpartum, or post- abortion. The survey includes a number of questions on your knowledge, attitudes, and practices around maternal and neonatal health. This is not a test; this is an opportunity to let us know your thoughts and experience on the topic as a healthcare provider in one of the hospitals participating in the study.

This survey is voluntary, and your answers will be kept confidential, and you can choose whether to leave some questions unanswered. General information about you, your position, and geographical location will be collected to help us categorize respondents only but will not be used to identify you in particular. You are free to provide this information at the end of the survey.

After the study, and only if you agree, a second online survey will be sent to you via email. For this reason, we will ask you to provide an email address so that we can ensure delivery of the second survey. You will be free to decide to participate in this second survey too. Results of these surveys will be published in a peer-reviewed journal without attributing responses to any specific person or institution.

The completion of this survey implies your consent to participate.

If you have any question about the survey, please contact Ms Vanessa Brizuela (brizuelav@who.int)

For peer review only - http://bmjopen.bmj.com/site/about/guidelines.xhtml

I. Knowledge and attitudes

The following questions will ask that you respond according to your current role, competences, and skills depending on your training and background. That is, according to these, you may be the person triaging, prescribing, diagnosing, treating. Bear this in mind when responding.

1. What are the main conditions causing death and disability among women during pregnancy and/or childbirth in your hospital? Check all that apply [abortion-related complications, chronic/pre-existing disease, embolism, haemorrhage, infection/sepsis, pre-eclampsia/eclampsia, other: please specify]
2. Case vignettes:

   Case A: A 25-year-old 32- week pregnant woman comes to your facility brought by a family member saying she is feeling unwell. Her companion reports that she seems a bit disoriented and feverish. Without any further diagnostic testing or triaging:

   a. What would you first think could be causing her to feel this way? Choose from the following list [abortion-related complications, embolism, haemorrhage, infection/sepsis, pre-eclampsia/eclampsia, other]
   b. What would be the first two things this woman should receive? [antibiotics, blood transfusion, body fluid culture, fetal monitoring, fluids, haematology/biochemistry laboratory, other antimicrobials (i.e. antimalarials, ART), other laboratory test, other medication, oxygen, physical exam, urine output measurement, other]
3. Case B: A recently pregnant woman comes to your facility complaining that she has abdominal pain and shortness of breath. Without any further diagnostic testing or triaging:
   a. What would you first think could be causing her to feel this way? Choose from the following list [abortion-related complications, embolism, haemorrhage, infection/sepsis, pre-eclampsia/eclampsia, other]
   b. What would be the first two things this woman should receive? [antibiotics, blood transfusion, body fluid culture, fetal monitoring, fluids, haematology/biochemistry laboratory, other antimicrobials (i.e. antimalarials, ART), other laboratory test, other medication, oxygen, physical exam, urine output measurement, other]
4. How confident do you feel that you are capable of making the right decision in a case like the one above? [very confident, somewhat confident, neutral, not too confident, not confident at all]
5. How would you qualify the availability of resources in the facility where you work to help you make the right decisions? [always available, somewhat available, neutral, not always available, not available at all].
6. How supported do you feel by the facility in which you work to make the right decision in a case like the one above? [very supported, somewhat supported, neutral, not very supported, unsupported].
7. How well does this statement describe your facility: “The facility where I work doesn’t let me handle cases like the one described above.” [very well, somewhat well, indifferent, somewhat incorrectly, completely incorrectly]

For peer review only - http://bmjopen.bmj.com/site/about/guidelines.xhtml

8. Of the following, which do you think are the greatest barriers in making a right, and timely decision in your facility? Check up to two options. [I'm afraid of making a mistake, I've never seen cases like these, my supervisor doesn't let me make them, not sure I know the correct signs, we don't have a way to triage/treat/manage cases like these in my hospital, other]
9. Does the hospital you work in have protocols in place for dealing with cases like the one described above? [yes/no/don't know]

DO NOT ALLOW GOING BACK AFTER THIS QUESTION

10. Have you ever heard of the term maternal sepsis? [yes/no]
11. If yes, how did you hear about this? Check all that apply [pre-service training, in-service training, public health campaign, colleagues, media (TV/radio/newspaper), other: please specify].
12. What two criteria best describe maternal sepsis? Check two options [abnormal white cell count, altered mental status, elevated heart rate, excessively rapid respiration, fever, infection, low blood pressure, organ dysfunction, other]
13. What supplies/commodities are essential to effectively identify sepsis among women during pregnancy, childbirth, postpartum or post-abortion? Check all that apply [blood culture, blood pressure apparatus, diagnostic imaging, laboratory (haematology/biochemistry), rapid test for infectious disease, serum lactate measurement, thermometer, urine output measurement, other]
14. What supplies/commodities are essential to effectively manage sepsis in women during pregnancy, childbirth, postpartum or post-abortion? Check all that apply [antibiotics, blood transfusions, fluids, intensive care/high-dependency unit, other antimicrobials (e.g. antimalarials, ART), oxygen, urine output measurement, other]

II. Context

15. How many women are affected by maternal sepsis in your facility every year? Give your best estimate (a whole number), given your experience in the facility [Text box]
16. How many neonates are affected by neonatal sepsis in the first week of life in your facility? Give your best estimate (a whole number), given your experience in the facility. [Text box]
17. How many deliveries occur every year, on average, in your facility? Give your best estimate (a whole number). [Text box]
18. Have you ever received specific training in how to manage women who present with signs of infection while pregnant, during childbirth, postpartum or post-abortion? [yes/no/can't remember]

III. Personal information

Remember! These data are collected for categorization purposes only. Your information is confidential and you will not be identified in any future publications on this study.

19. Age range
20. Gender: [male, female, other]

For peer review only - http://bmjopen.bmj.com/site/about/guidelines.xhtml

21. Qualification: [nurse, midwife, physician/medical doctor, resident/physician in training, community health worker, social worker, other: please specify].
22. Years of work experience in current setting: [years|months]
23. Total years of work experience (since completing your training): [number entry/2-digit max]
24. Place of work: [list of countries]
25. Location (of current or main place of work): [urban/rural]
26. Name of facility & address [Text box]
27. Facility type (of current or main place of work): [Clinic, Health centre, Maternity hospital, Regional/Provincial hospital, District hospital, Other hospital, other]
28. Facility management (of current or main place of work): [private, public, social insurance, NGO, other]
29. Did you participate in this year's World Sepsis Congress Spotlight on Maternal and Neonatal Sepsis (held on 12 September 2017)? [yes/no]

IV. Future contacts

30. The global maternal study and awareness campaign would like to contact you at a future date for a follow-up on this survey. If you agree to being contacted again, please provide us with your email address.

Your contact details will be securely stored by the WHO staff person working on the study for one year. You can contact us to modify or suppress your information at any time. To do so, please contact Ms Vanessa Brizuela (brizuelav@who.int).

- ☐ I agree.
- ☐ I do not agree

Please provide us with your email address [text box]

Thank you very much for participating in this survey! Your responses are extremely valuable to us in our efforts to improve the health of women and newborns.

For peer review only - http://bmjopen.bmj.com/site/about/guidelines.xhtml

GLOSS Post-Campaign Survey

This online survey is part of the activities set forth for a global study on maternal morbidity and mortality. This study is being conducted in approximately 50 countries across the globe, including your own, and it is coordinated by the World Health Organization and the healthcare facility where you work. As part of this study, we want to learn more from healthcare providers about how you identify and manage women with certain conditions during pregnancy, childbirth or postpartum or post- abortion.

A few months ago, you received a similar survey from us. At that point we asked that you provide us with an email address so that we could send you a follow-up questionnaire. We will ask again that you respond to a number of questions on your knowledge, attitudes, and practices around maternal and neonatal health. This is not a test; this is an opportunity to let us know your thoughts and experience on the topic as a healthcare provider in one of the hospitals participating in the study.

This survey is voluntary, and your answers will be kept confidential, and you can choose whether to leave some questions unanswered. General information about you, your position, and geographical location will be collected to help us categorize respondents only but will not be used to identify you in particular. You are free to provide this information at the end of the survey. Results of these surveys will be published in a peer-reviewed journal without attributing responses to any specific person or institution.

The completion of this survey implies your consent to participate.

If you have any question about the survey, please contact Ms Vanessa Brizuela (brizuelav@who.int)

For peer review only

For peer review only - http://bmjopen.bmj.com/site/about/guidelines.xhtml

I. General knowledge and attitudes

The following questions will ask that you respond according to your current role, competences, and skills depending on your training and background. According to these, you may be the person triaging, prescribing, diagnosing, treating. Bear this in mind when responding.

1. What are the main conditions causing death and disability among women during pregnancy and/or childbirth in your hospital? Check all that apply [abortion-related complications, chronic/pre-existing disease, embolism, haemorrhage, infection/sepsis, pre-eclampsia/eclampsia, other: please specify]
2. Case vignettes:

   Case A: A 25-year-old 32- week pregnant woman comes to your facility brought by a family member saying she is feeling unwell. Her companion reports that she seems a bit disoriented and feverish. Without any further diagnostic testing or triaging:

   a. What would you first think could be causing her to feel this way? Choose from the following list [abortion-related complications, embolism, haemorrhage, infection/sepsis, pre-eclampsia/eclampsia, other]
   b. What would be the first two things this woman should receive? [antibiotics, blood transfusion, body fluid culture, fetal monitoring, fluids, haematology/biochemistry laboratory, other antimicrobials (i.e. antimalarials), other laboratory test, other medication, oxygen, physical exam, urine output measurement, other]
3. Case B: A recently pregnant woman comes to your facility complaining that she has abdominal pain and shortness of breath. Without any further diagnostic testing or triaging:
   a. What would you first think could be causing her to feel this way? Choose from the following list [abortion-related complications, embolism, haemorrhage, infection/sepsis, pre-eclampsia/eclampsia, other]
   b. What would be the first two things this woman should receive? [antibiotics, blood transfusion, body fluid culture, fetal monitoring, fluids, haematology/biochemistry laboratory, other antimicrobials (i.e. antimalarials, ART), other laboratory test, other medication, oxygen, physical exam, urine output measurement, other]
4. How confident do you feel that you are capable of making the right decision in a case like the one above? [very confident, somewhat confident, indifferent/neutral, not too confident, not confident at all] n
5. How would you qualify the availability of resources in the facility where you work to help you make the right decisions? [always available, somewhat available, indifferent/neutral, not always available, not available at all].
6. How supported do you feel by the facility in which you work to make the right decision in a case like the one above? [very supported, somewhat supported, neutral, not very supported, unsupported].
7. How well does this statement describe your facility: "The facility where I work doesn't let me handle cases like the one described above." [very well, somewhat well, indifferent/neutral, somewhat incorrectly, completely incorrectly]

For peer review only - http://bmjopen.bmj.com/site/about/guidelines.xhtml

8. Of the following, which do you think are the greatest barriers in making a right, and timely decision in your facility? Check one option (the one most accurate to your situation). [not sure I know the correct signs, I'm afraid of making a mistake, my supervisor doesn't let me make them, we don't have a way to triage/treat/manage cases like these in my hospital, I've never seen cases like these, There are no barriers to making a right and timely decision in my facility, other]
9. Does the hospital you work in have protocols in place for dealing with cases like the one described above? [yes/no/don't know]

DO NOT ALLOW GOING BACK AFTER THIS QUESTION

10. Have you ever heard of the term maternal sepsis? [yes/no]
11. If yes, how did you hear about this? Check all that apply [pre-service training, in-service training, colleagues, public health campaign, media (TV/radio/newspaper), other: please specify].
12. What two criteria best describe maternal sepsis? Check two options [abnormal white cell count, altered mental status, elevated heart rate, excessively rapid respiration, fever, infection, low blood pressure, organ dysfunction, other]
13. What supplies/commodities are essential to effectively identify sepsis among women during pregnancy, childbirth, postpartum or post-abortion? Check all that apply [blood culture, blood pressure apparatus, diagnostic imaging, laboratory (haematology/biochemistry), rapid test for infectious disease, serum lactate measurement, thermometer, urine output measurement, other]
14. What supplies/commodities are essential to effectively manage sepsis in women during pregnancy, childbirth, postpartum or post-abortion? Check all that apply [antibiotics, blood transfusions, fluids, intensive care/high-dependency unit, other antimicrobials (e.g. antimalarials, ART), oxygen, urine output measurement, other]

II. Context

15. How many women are affected by maternal sepsis in your facility every year? Give your best estimate (a whole number), given your experience in the facility [Text box]
16. How many neonates are affected by neonatal sepsis in the first week of life in your facility? Give your best estimate (a whole number), given your experience in the facility. [Text box]
17. How many deliveries occur every year, on average, in your facility? Give your best estimate (a whole number). [Text box]
18. Have you ever received specific training in how to manage women who present with signs of infection while pregnant, during childbirth, postpartum or post-abortion? [yes/no/can't remember]

III. Campaign

19. Did you notice any materials in your facility that related to maternal and neonatal sepsis? [yes/no] [if no go to question 27]

For peer review only - http://bmjopen.bmj.com/site/about/guidelines.xhtml

20. If yes, where did you see them? Check all that apply [labour ward, antenatal ward, postnatal ward, emergency room/department, ICU/high dependency room, patient waiting area, other: please specify]
21. If yes, what materials did you see? Check all that apply [informational posters about the study, graphic materials with information about maternal sepsis, campaign website, social media, press releases, none of the above].
    a. Did you ever visit the website? [yes/no/N/A]
    b. Did you ever tweet/re-tweet a message about the campaign? [yes/no/I don't use Twitter]
22. What were the main messages of these materials? Check all that apply ["stop sepsis", "think sepsis", "right care, right now", "sepsis is life-threatening but when caught early and treated promptly it can be stopped", "just ask, could it be sepsis?", "a world free of sepsis", "together, we can stop maternal sepsis", "stop sepsis save lives", "surviving sepsis", none of the above]
23. Did you find the materials and messaging helpful? [yes/no]
    a. If yes, why? Check all that apply [they provided information about maternal sepsis, they invited me to act, they mentioned things I did not know, they helped explain the study to me, they were visually appealing, they were in a language I could understand, other]
    b. If no, why not? Check all that apply [they didn't provide any new information, I didn't know what to do, the message was confusing, they were difficult to understand, they were in a language I don't speak/read, they had too much text, they had too many images, the colours didn't work in our hospital, they were unappealing, other]
24. Did the materials help you identify cases of women with infection or sepsis? [yes/no]
25. Did the information provided in the materials motivate you to do something differently than before? [yes/no]
    a. If yes, in what way? Check all that apply [to suspect maternal sepsis among women presenting with specific signs, to act fast when I think a woman could have sepsis, to implement health measures to prevent sepsis, to talk about sepsis with others, to learn more about sepsis, other: please specify]
    b. If no, why not? Please explain [text box]
26. In your opinion, did the materials help to increase awareness about maternal sepsis in your facility? [yes/no]
27. Did you know that your facility participated in a global maternal sepsis study? [yes/no]
28. Did you know that your facility participated in an awareness campaign for the global maternal sepsis study? [yes/no]

IV. Personal information

Remember! These data are collected for categorization purposes only. Your information is confidential and you will not be identified in any future publications on this study.

29. Age range [18-25; 26-30; 31-40; 41-50; 51-60; 60+]
30. Gender: [male, female, other]
31. Qualification: [community health worker, midwife, nurse, physician/medical doctor, resident/physician in training, social worker, student, physical therapist, auxiliary nurse, other: please specify].

For peer review only - http://bmjopen.bmj.com/site/about/guidelines.xhtml

32. Years of work experience in current setting: [years | months]
33. Total years of work experience (since completing your training): [2-digit max]
34. Place of work: [list of countries]
35. Location (of current or main place of work): [urban/rural]
36. Name of facility and address [text entry]
37. Facility type (of current or main place of work): [Clinic, Health centre, Maternity hospital, Regional/Provincial hospital, District hospital, Other hospital, other]
38. Facility management (of current or main place of work): [private, public, social insurance, NGO, other]
39. Did you participate in the World Sepsis Congress Spotlight on Maternal and Neonatal Sepsis (held on 12 September 2017)? [yes/no]

V. Future contacts

We might be interested in contacting you in the future with more information about this study or to get further information about your responses to this survey. Please provide us with your email address below if you agree with this.

- ☐ I agree.
- ☐ I do not agree

Your contact details will be securely stored by the WHO staff person working on the Global Maternal and Neonatal Initiative for one year. You can contact us to modify or suppress your information at any time. To do so, please contact Ms Vanessa Brizuela (brizuelav@who.int)

40. Please type your email address below [text entry]

Thank you very much for participating in this survey! Your responses are extremely valuable to us in our efforts to improve the health of women and babies.

Please visit http://srhr.org/sepsis for more information about the study, the campaign, and maternal sepsis.

For peer review only - http://bmjopen.bmj.com/site/about/guidelines.xhtml

Appendix 2. Respondent perception of enabling environments for maternal sepsis identification and management at pre- and post-campaign and changes after campaign implementation, limiting to most favourable responses (N=2,188)

	Pre-campaign n/N (%)	Post-campaign n/N (%)	Pre-post cOR‡ [95% CI]¶	Percentage change %
Perception of enabling environment for maternal sepsis identification and management				
Very confident of making right decisions	390/1,155 (33.8)	395/1,033 (38.2)	1.21* [1.05-1.41]	13.2
Resources ***always*** available to make right decisions	443/1,155 (38.4)	386/1,033 (37.4)	0.96 [0.68-1.35]	-2.6
Very supported by facility in making right decisions	503/1,155 (43.6)	438/1,033 (42.4)	0.95 [0.67-1.37]	-2.6

cOR: crude odds ratio; CI: confidence interval; OR calculated clustering at the country level
Percentage change: [(% in post - % in pre)/% in pre]x100
‡Refers to odds ratio between pre- and post-campaign; ¶reference group: pre-campaign; **p<0.05*

Responses were dichotomized as follows: a 1 was assigned to the most favourable response (i.e., they felt very confident *about being capable of making the right decision) and a 0 to a combination of all the remaining options (i.e.,* somewhat confident, neutral, not very confident *or* not confident at all*)*

For peer review only - http://bmjopen.bmj.com/site/about/guidelines.xhtml

Appendix 3. Changes in respondent knowledge and perception of enabling environments after campaign implementation among different sub-groups: [1] sample restricted to ≥1 response per facility at pre- and post-campaign; [2] sample restricted to ≥2 responses per facility at pre- and post-campaign; [3] sample restricted to countries with >30 responses at pre- or post-campaign

	**Full sample (N=2,188)**		Restricted sample ≥1 response per facility (N=1,872)		Restricted sample ≥2 responses per facility (N=1,645)		Restricted sample >30 responses per country (N=1,680)	
	**Pre-post OR‡ [95% CI]¶**	**% change**	Pre-post OR‡ [95% CI] ¶	% change	Pre-post OR‡ [95% CI] ¶	% change	Pre-post OR‡ [95% CI] ¶	% change
Knowledge on maternal sepsis								
Have not heard of maternal sepsis	**0.35* [0.18-0.68]**	**-63.4**	0.36* [0.17-0.77]	-61.82	0.45* [0.22-0.93]	-53.58	0.34* [0.15-0.79]	-63.29
Correctly identified the two criteria to define maternal sepsis	**0.67 [0.43-1.03]**	**-29.4**	0.63 [0.39-1.01]	-33.19	0.57* [0.34-0.93]	-38.76	0.57* [0.33-0.98]	-39.46
Correctly identified management of sepsis when maternal sepsis was suspected	**1.76 [0.73-4.21]**	**30.8**	1.58 [0.58-4.30]	24.05	1.53 [0.51-4.62]	21.70	1.70 [0.51-5.72]	24.89
Perception of enabling environment for maternal sepsis identification and management								
Confident of making right decisions	**1.44* [1.01-2.06]**	**7.3**	1.36 [0.97-1.90]	6.39	1.33 [0.94-1.88]	6.34	1.45 [0.95-2.22]	7.99
Resources available to make right decisions	**1.01 [0.68-1.49]**	**0.1**	1.12 [0.76-1.66]	2.32	1.12 [0.72-1.74]	2.23	0.92 [0.57-1.50]	-1.62
Supported by facility in making right decisions	**1.11 [0.80-1.54]**	**2.0**	1.21 [0.84-1.73]	3.44	1.19 [0.81-1.76]	3.45	0.99 [0.69-1.44]	-0.11

OR: odds ratio; CI: confidence interval; percentage change= [(% in post - % in pre)/% in pre]x100

‡Refers to odds ratio between pre- and post-campaign, OR calculated clustering at the country level; ¶reference group pre-campaign; **p<0.05*

For peer review only - http://bmjopen.bmj.com/site/about/guidelines.xhtml

Appendix 4a. Changes in respondent knowledge after campaign implementation according to respondent and facility characteristics (N= 2,188)

	Had not heard about maternal sepsis						Correctly identified the two criteria to define maternal sepsis						Correctly identified management of sepsis when maternal sepsis was suspected					
Respondent characteristics	*Pre-campaign (N=1,144)*		*Post-campaign (N=1,021)*		*OR [95% CI]¶*	*% change*	*Pre-campaign (N=673)*		*Post-campaign (N= 647)*		*OR [95% CI]¶*	*% change*	*Pre-campaign (N=251)*		*Post-campaign (N=239)*		*OR [95% CI]¶*	*% change*
	n	%	N	%		%	n	%	n	%		%	n	%	n	%		%
Overall	*95*	*8*	*31*	*3*	*0.35* [0.23-0.52]*	*-63.4*	*109*	*16*	*74*	*11*	*0.67 [0.43-1.03]*	*-29.4*	*114*	*45*	*142*	*59*	*1.76 [0.73-4.21]*	*30.8*
Qualification	*1,141*		*1,013*				*670*		*645*				*250*		*235*			
Nurse/midwife	74	17	16	4	0.18* [0.10-0.33]	-79.2	14	6	8	3	0.48 [0.20-1.14]	-50.3	24	28	41	46	2.13 [0.53-8.47]	61.3
Physician	16	3	10	2	0.77 [0.26-2.31]	-22.6	72	21	55	19	0.87 [0.55-1.38]	-10.3	55	50	75	67	2.06* [1.09-3.91]	35.1
Resident	4	3	4	4	1.36 [0.13-14.31]	34.3	23	27	11	15	0.48 [0.11-2.11]	-44.4	34	63	25	76	1.84 [0.49-6.90]	20.3
Years of experience	*1,098*		*963*				*642*		*607*				*242*		*229*			
<10	20	4	14	3	0.79 [0.28-2.24]	-19.9	58	18	32	11	0.54 [0.28-1.02]	-41.3	67	48	72	58	1.50 [0.40-5.58]	21.2
10-20	36	10	8	3	0.22* [0.10-0.50]	-75.9	28	15	24	12	0.76 [0.44-1.33]	-21.0	31	47	44	66	2.16* [1.04-4.49]	39.8
>20	26	12	4	2	0.17* [0.08-0.38]	-81.0	22	16	16	16	0.95 [0.53-1.69]	-4.4	13	37	21	57	2.22 [0.72-6.87]	52.8
Region	*1,144*		*1,021*				*673*		*647*				*251*		*239*			
Africa	14	6	4	2	0.27* [0.15-0.49]	-71.6	18	15	19	15	1.04 [0.46-2.31]	3.1	30	53	46	69	1.97 [0.95-4.08]	30.5
Asia	11	7	9	5	0.83 [0.25-2.76]	-15.7	7	5	7	6	1.06 [0.42-2.65]	5.7	4	14	8	23	1.85 [0.37-9.21]	65.8
Eastern Mediterranean	58	44	13	8	0.16* [0.09-0.31]	-81.7	2	3	8	9	3.19 [1.04-9.78]	200.0	2	12	10	45	6.25 [0.88-44.14]	286.5
Europe‡	7	5	2	2	0.39 [0.07-2.23]	-59.7	27	38	12	27	0.63* [0.41-0.96]	-27.3	5	12	4	27	1.60 [0.77-3.33]	126.8
Latin America	5	1	3	1	0.72 [0.07-7.77]	-27.9	55	20	28	11	0.49 [0.22-1.08]	-45.8	73	12	74	74	1.87 [1.17-20.42]	529.3

For peer review only

For peer review only - http://bmjopen.bmj.com/site/about/guidelines.xhtml

Country implemented an expanded version of campaign	*1,144*		*1,021*				*673*		647				251		239			
Yes	74	11	13	3	0.23* [0.16-0.32]	-75.2	75	19	37	12	0.58 [0.32-1.05]	-37	92	53	105	76	2.77 [0.88-8.69]	42
No	21	5	18	3	0.71 [0.35-1.45]	-27.6	34	12	37	11	0.89 [0.52-1.53]	-10	22	28	37	38	1.50 [0.51-4.40]	32
Respondent worked in a level III facility	*1,144*		*1,021*				*673*		*647*				*251*		*239*			
Yes	23	3	16	3	0.91 [0.38-2.18]	-8.5	87	18	58	15	0.80 [0.45-1.41]	-17.1	104	51	108	65	1.77 [0.51-6.10]	27.0
No	72	20	15	4	0.15* [0.08-0.28]	-82.3	22	11	16	6	0.51 [0.25-1.03]	-46.3	10	21	34	47	3.31* [1.54-7.12]	123.6

‡Includes countries in Central Asia (Kazakhstan, Kyrgyzstan, and Tajikistan)

OR: odds ratio, OR calculated clustering at the country level; CI: confidence interval; Percentage change= [(% in post - % in pre)/% in pre]x100

¶Reference group: pre-campaign

Where n represents the frequency and N the denominator (i.e. n= the number of respondents with a specific characteristic who answered correctly, N= the total number of persons who answered that question)

For peer review only - http://bmjopen.bmj.com/site/about/guidelines.xhtml

Appendix 4b. Changes in respondent perception of enabling environments after campaign implementation according to respondent and facility characteristics

Respondent characteristic	*Confident of making right decisions*						*Resources available to make right decisions*						*Supported by facility in making right decisions*					
	Pre-campaign (N=1,155)		*Post-campaign (N=1,033)*		*OR [95% CI]¶*	*% change*	*Pre-campaign (N=1,155)*		*Post-campaign (N=1,033)*		*OR [95% CI]¶*	*% change*	*Pre-campaign (N=1,155)*		*Post-campaign (N=1,033)*		*OR [95% CI]¶*	*% change*
	n	*%*	*n*	*%*		*%*	*n*	*%*	*n*	*%*		*%*	*N*	*%*	*n*	*%*		*%*
Overall	*897*	*78*	*861*	*83*	*1.44* [1.01-2.06]*	*7.3*	*909*	*79*	*814*	*79*	*1.01 [0.68-1.49]*	*0.1*	*921*	*80*	*840*	*81*	*1.11 [0.80-1.54]*	*2.0*
Qualification	*1,151*		*1,025*				*1,151*		*1,025*				*1,151*		*1,025*			
Nurse/midwife	355	81	371	81	1.05 [0.56-1.96]	0.8	356	81	336	74	0.66 [0.34-1.28]	-8.9	367	83	347	76	0.63* [0.41-0.98]	-8.8
Physician	440	78	395	87	1.78* [1.23-2.57]	10.4	445	79	387	85	1.46* [1.17-1.82]	7.0	448	80	393	86	1.57* [1.12-2.22]	7.9
Resident	100	67	87	77	1.67 [0.97-2.88]	15.5	105	70	86	76	1.37 [0.77-2.42]	8.7	104	69	95	84	2.33* [1.24-4.38]	21.3
Years of experience	*1,107*		*970*				*1,107*		*970*				*1,107*		*970*			
<10	409	76	389	82	1.44 [0.99-2.10]	8.1	416	77	379	80	1.17 [0.80-1.73]	3.6	424	78	390	82	1.25 [0.86-1.83]	4.5
10-20	284	81	264	83	1.08 [0.73-1.59]	1.4	277	79	239	75	0.77 [0.52-1.13]	-5.9	280	80	248	78	0.85 [0.59-1.23]	-3.4
>20	179	82	160	92	2.43* [1.13-5.21]	11.5	187	86	153	88	1.17 [0.72-1.91]	2.0	188	87	158	91	1.52 [0.93-2.49]	4.8
Region	*1,155*		*1,033*				*1,155*		*1,033*				*1,155*		*1,033*			
Africa	189	84	198	88	1.31 [0.76-2.26]	3.8	158	71	157	69	0.95 [0.73-1.24]	-1.5	176	79	173	77	0.89 [0.63-1.26]	-2.6
Asia	128	74	145	85	2.04* [1.17-3.55]	15.3	134	77	150	88	2.18* [1.72-2.76]	13.9	137	79	151	89	2.09* [1.59-2.74]	12.2
Eastern Mediterranean	131	77	115	70	0.70* [0.64-0.77]	-9.0	109	64	84	51	0.59* [0.49-0.71]	-20.1	108	63	97	59	0.83 [0.61-1.14]	-6.9
Europe‡	97	71	77	79	1.59 [0.89-2.82]	12.1	112	82	83	86	1.32 [0.80-2.20]	4.7	112	82	88	91	2.18* [1.54-3.09]	11.0
Latin America	352	78	326	87	1.85* [1.14-3.01]	11.1	396	88	340	91	1.32 [0.68-2.57]	3.0	388	86	331	88	1.20 [0.59-2.44]	2.4

For peer review only

For peer review only - http://bmjopen.bmj.com/site/about/guidelines.xhtml

Country implemented an expanded version of campaign	*1,155*		*1,033*				*1,155*		*1,033*				*1,155*		*1,033*			
Yes	531	75	398	80	1.28 [0.76-2.15]	5.7	554	79	375	75	0.82 [0.45-1.48]	-4.6	567	80	391	78	0.87 [0.53-1.43]	-2.8
No	366	81	463	87	1.52* [1.00-2.29]	6.8	355	79	439	82	1.25 [0.83-1.89]	4.4	354	79	449	84	1.45* [1.03-2.05]	7.1
Respondent worked in a level III facility	*1,155*		*1,033*				*1,155*		*1,033*				*1,155*		*1,033*			
Yes	608	77	508	83	1.51* [1.10-2.05]	8.4	646	82	516	85	1.24 [0.85-1.79]	3.6	640	81	518	85	1.33 [0.95-1.87]	5.0
No	289	79	353	83	1.31 [0.77-2.22]	5.1	263	72	298	70	0.92 [0.62-1.36]	-2.5	281	77	322	76	0.94 [0.71-1.25]	-1.4

‡Includes countries in Central Asia (Kazakhstan, Kyrgyzstan, and Tajikistan)

OR: odds ratio, OR calculated clustering at the country level; CI: confidence interval; Percentage change= [(% in post - % in pre)/% in pre]x100

¶Reference group: pre-campaign

Where n represents the frequency and N the denominator (i.e. n= the number of respondents with a specific characteristic who answered correctly, N= the total number of persons who answered that question)

For peer review only

For peer review only - http://bmjopen.bmj.com/site/about/guidelines.xhtml
